# Synthesis and Activity of Triazole-Adenosine Analogs as Protein Arginine Methyltransferase 5 Inhibitors

**DOI:** 10.3390/molecules27123779

**Published:** 2022-06-11

**Authors:** Tyler Brown, Mengtong Cao, Y. George Zheng

**Affiliations:** Department of Pharmaceutical and Biomedical Sciences, College of Pharmacy, University of Georgia, Athens, GA 30602, USA; tyler.brown27@uga.edu (T.B.); mc73551@uga.edu (M.C.)

**Keywords:** epigenetics, arginine methylation, PRMT5, click chemistry, inhibitor, SAM analog

## Abstract

Protein arginine methyltransferase 5 (PRMT5) is an attractive molecular target in anticancer drug discovery due to its extensive involvement in transcriptional control, RNA processing, and other cellular pathways that are causally related to tumor initiation and progression. In recent years, various compounds have been screened or designed to target either the substrate- or cofactor-binding site of PRMT5. To expand the diversity of chemotypes for inhibitory binding to PRMT5 and other AdoMet-dependent methyltransferases, in this work, we designed a series of triazole-containing adenosine analogs aimed at targeting the cofactor-binding site of PRMT5. Triazole rings have commonly been utilized in drug discovery due to their ease of synthesis and functionalization as bioisosteres of amide bonds. Herein, we utilized the electronic properties of the triazole ring as a novel way to specifically target the cofactor-binding site of PRMT5. A total of about 30 compounds were synthesized using the modular alkyne-azide cycloaddition reaction. Biochemical tests showed that these compounds exhibited inhibitory activity of PRMT5 at varying degrees and several showed single micromolar potency, with clear selectivity for PRMT5 over PRMT1. Docking-based structural analysis showed that the triazole ring plays a key role in binding to the characteristic residue Phe327 in the active pocket of PRMT5, explaining the compounds’ selectivity for this type-II enzyme. Overall, this work provides new structure–activity relationship information on the design of AdoMet analogs for selective inhibition of PRMT5. Further structural optimization work will further improve the potency of the top leads.

## 1. Introduction

Protein arginine methylation is a widespread post-translational modification (PTM) that occurs across eukaryotic organisms [1,2]. This biochemical reaction is mediated by the family of protein N-arginine methyltransferases (PRMTs) [3]. These enzymes catalyze the transfer of a methyl group from *S*-adenosyl-l-methionine (SAM) onto the terminal nitrogens of the guanidino group of the arginine side chain, producing *S*-adenosyl-l-homocysteine (SAH) as a byproduct. The nine members of the human PRMT family are classified into three types based on their end-methylation products. All members can catalyze the first methylation step, which forms N^G^-monomethylarginine (MMA), and additionally type I and type II PRMTs can further catalyze a second methylation reaction. Type I PRMTs (PRMT1, -2, -3, -4, -6, -8) add a second methyl group onto the same nitrogen as the first to form N^G^, N^G^-dimethylarginine (ADMA). Type II PRMTs (PRMT5, -9) add a second methyl group onto the other omega nitrogen to form N^G^, N’^G^-dimethylarginine (SDMA). PRMT7 is a type III enzyme and can only catalyze the formation of MMA [4]. Gene knock-out experiments showed that PRMT1 is the major type I enzyme, accounting for over 50% of the production of ADMA in cells, while PRMT5 is the major type II enzyme, accounting for over 90% of the production of SDMA [5,6,7]. Through protein methylation, PRMTs are able to regulate a variety of cellular processes, with the most substantial being the regulation of gene transcription via the modification of nucleosomal histones and the regulation of RNA processing via modification of major RNA-binding proteins [8,9,10].

Like all the other PRMTs, PRMT5 contains a Rossmann fold domain for SAM binding and a β-barrel that participates in substrate recognition and binding. In addition to these characteristic structural domains, PRMT5 also contains an N-terminal TIM barrel domain that binds to the WD40 repeat protein MEP50, which stabilizes PRMT5 and dramatically improves its catalytic activity [11]. PRMT5 has a diverse set of substrates spanning nucleosomal histones [2,11], p53 [12], EGFR [13], and RUVBL1 [14]. It also plays a role in RNA splicing, where it methylates the spliceosome, which can have significant downstream implications in RNA transcripts [15,16]. For example, inhibition of PRMT5 leads to an increase in aberrant splicing events in various cancer cell lines and leads to skipping of exon 4 in MDM4, which results in an inability to inhibit p53 and thus cause cell death in those cell lines with wild-type p53 [16]. With an intimate involvement in transcriptional and post-transcriptional regulation, it is not surprising that aberrant expression of PRMT5 is associated with a variety of cancers, including small cell lung cancer, breast cancer, ovarian cancer, and acute lymphoblastic lymphoma [8,16,17,18].

**Figure 1:**
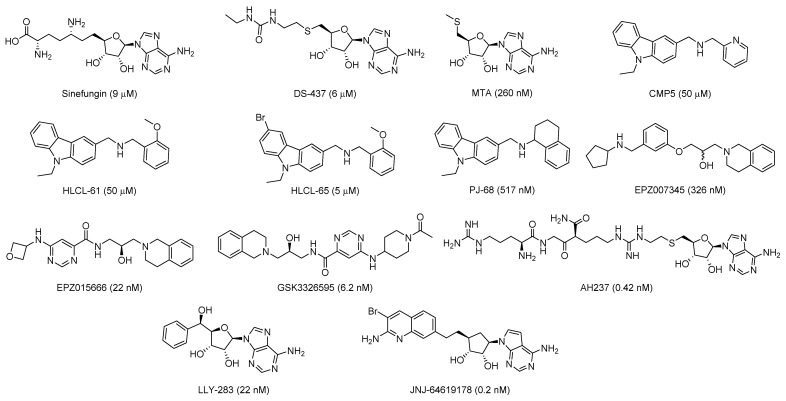
Representative structures of the reported inhibitors of PRMT5. Both JNJ-64619178 (a SAM-competitive inhibitor) and GSK3326595 (a substrate-competitive inhibitor) are in phase I clinical trials. The third clinical trial molecule: PF-06939999, does not have a structure reported.

The intense involvement in cancer pathology has led to an increasing recognition of PRMT5 being a novel holy grail target for the development of chemotherapeutics. PRMT inhibition has been a topic of intense investigation in recent years [19,20,21,22]. The first class of inhibitors identified were SAM analogs with pan-MTase inhibitory activity, SAH [23] and sinefungin (Figure 1), with the latter being isolated from *S. griseolus* in 1979 [24]. Great effort was made towards the development of more specific inhibitors for PRMTs and other protein methyltransferases. One of the first selective inhibitors was DS-437: a dual PRMT5/PRMT7 inhibitor with an IC_50_ of 6 μM [25]. While it is a close analog of SAM, surprisingly, DS-437 was inactive towards 29 other human methyltransferases. Another selective inhibitor that was discovered was S-methylthioadenosine (MTA). MTA is a component of the methionine salvage pathway in cells and is a substrate of methylthioadenosine phosphorylase (MTAP). It has been shown that deletion of MTAP in cancer cells is common due to its proximity to CDKN2A, a commonly deleted gene in cancer [26]. Upon deletion of MTAP, MTA accumulates in the cell and results in the selective inhibition of PRMT5 [26]. While MTA is a great tool molecule for PRMT5 inhibition, it suffers drawbacks in cells as it requires cells to be MTAP^-/-^; otherwise, it is rapidly metabolized.

The next series of inhibitors were N-alkyl-9H-carbazoles, which were found to be both substrate and cofactor competitive. The first of these inhibitors was CMP5, which had a modest potency of 50 μM [27]. CMP5 was further optimized to yield both HLCL-61 [28] and HLCL-65 [29], with increased potencies compared to their parent compound. Another inhibitor PJ-68 was also developed with an even further increased potency and showed a significant increase in survival in a murine model of BCR-ABL-driven CML and inhibited the in vivo self-renewal of CML leukemia stem cells [30].

Tetrahydroisoquinoline-based compounds have also been developed as PRMT5 inhibitors. The first compound developed was EPZ007345, which had a potency of 326 nM; however, it had poor in vitro clearance [31]. Further optimization led to the development of EPZ015666, which showed both improved potency and clearance [31]. A third inhibitor of this class: GSK3326595 (Figure 1), was subsequently developed and has since entered into clinical trials [16]. Recently, Shen et al. designed the first-in-class PRMT5 degraders by covalently linking the analogs of GSK3326595 with a von Hippel–Lindau (VHL) E3 ligase-targeting ligand [32]. Another recent inhibitor identified was AH437, which is a dual PRMT4/PRMT5 bisubstrate inhibitor with an IC_50_ of 0.42 nM [33].

In addition to GSK3326595, two other molecules have also entered clinical trials [34]. JNJ-64619178 is a unique inhibitor as it was one of the first SAM-competitive, selective PRMT5 inhibitors identified (the other being LLY-283) [35,36]. It was found to be a bisubstrate inhibitor with an IC_50_ of 0.15 nM and pseudo-irreversible inhibition kinetics. It is currently in clinical trials for various lung cancers. The other molecule: PF-0693999 (structure not disclosed), has recently entered clinical trials [34].

Despite the above development, the types of compounds for PRMT5 inhibition are still very limited. Chemical targeting of PRMT5 requires further endeavors in the medicinal chemistry field for improved potency, selectivity, efficacy, and other key pharmacologic attributes to serve the need of both basic biology and cancer drug discovery. Taking all of these issues into consideration, we set out to explore the new, diversifiable SAM analog structures for the inhibition of PRMT5 and other protein methyltransferases. In this work, we report a set of triazole-containing adenosine derivatives for PRMT5 inhibition [35,37].

## 2. Results and Discussion

### 2.1. Design of Triazole-Adenosine Analog Inhibitors of PRMT5

**Scheme 1:**
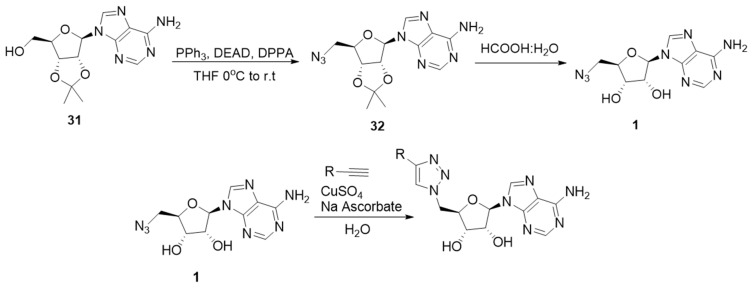
Synthesis of the scaffolding molecule **1** and modular synthesis of triazole-adenosine analogs.

Our design of the SAM analogs began with a structural investigation into the SAM-binding pocket of PRMT5. Within the Rossmann fold, PRMT5 harbors a unique residue: Phe327. This specific phenylalanine is found only in PRMT5 and is important for the product specificity of PRMT5, where mutation of F327 to methionine, its homologous residue in other PRMTs, results in the formation of both SDMA and ADMA [38]. Phe327 has been utilized as a drug-binding point to obtain specificity of inhibitors for PRMT5 over other SAM-binding enzymes through pi–cation interaction, as seen in the development of LLY-283 [35] and JNJ-64619178 [37]. In designing our potential inhibitors, we took this into this context and sought to develop an easily diversifiable set of inhibitors that contain triazole-adenosine as part of the pharmacophore (Scheme 1). The triazole moiety has a unique advantage of being easy to synthesize and diversify through the modular Cu(I)-catalyzed azide-alkyne click chemistry. Additionally, triazole-containing adenosine analogs have been previously designed as part of inhibitory screening of other enzymes such as α-2,3-sialyltransferase [39] and NTMT1 [40], and we believed that this pharmacophore was well tolerated and could be applied to our own screening with PRMTs.

**Figure 2:**
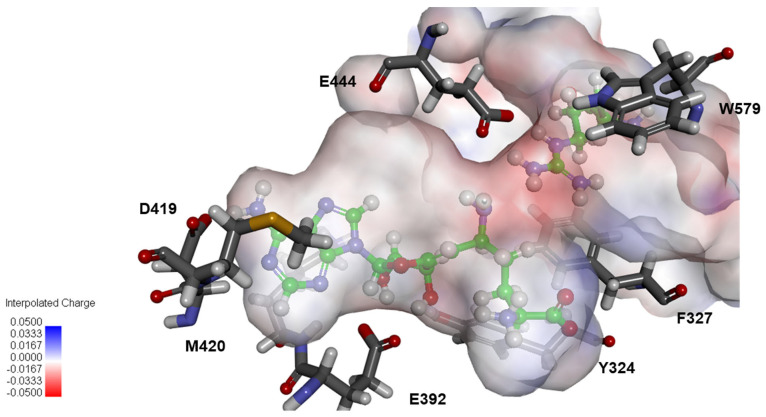
Crystal structure positions of dehydrosinefungin (below) and H4R3 (above). PDB ID: 4GQB.

**Figure 3:**
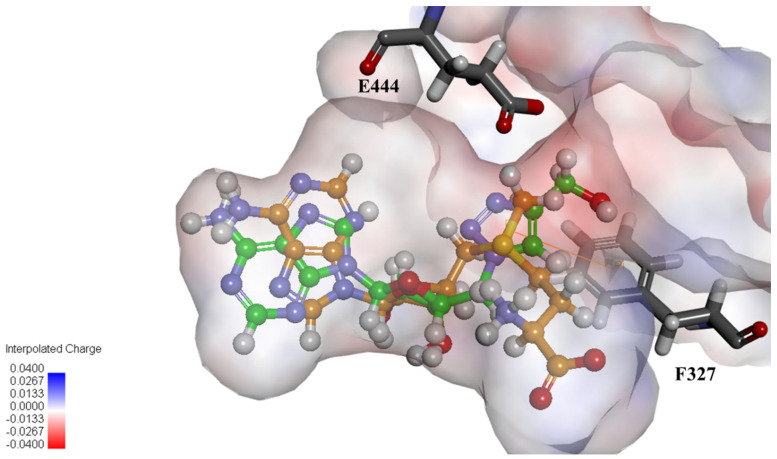
Overlay of docked SAM (orange) and **3** (green) showing the position of the triazole ring in relation to the sulfonium portion of SAM.

**Figure 4:**
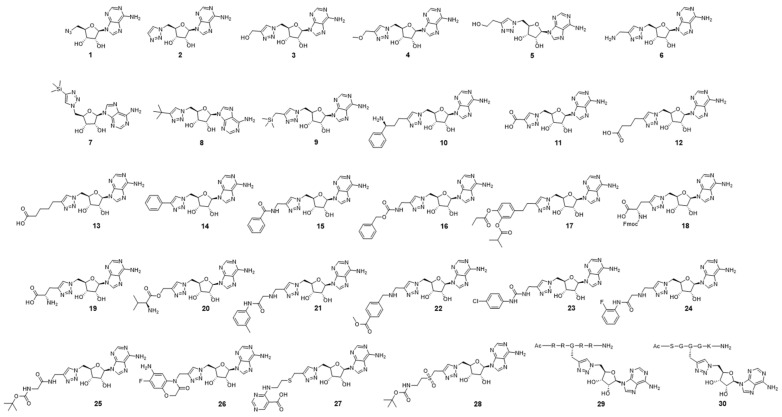
Synthesized triazole containing adenosine analogs.

We first conducted a virtual screening of triazole-adenosine analogs containing variable substituents on the 4-position of the triazole ring, with a total number of 1230 molecules as part of a library of terminal alkynes from Enamine. The protein structure was acquired from the PDB (PDB ID: 4GQB) and prepared in Discovery Studios^®^. A binding site was established in the prepared protein structure, which was bordered by key residues involved in both cofactor and substrate binding [11,38] and involved Lys393, Met420, and D419 in the purine-binding pocket; Tyr324 and Glu392 that bind to the 2′ and 3′-hydroxyls; and Phe327, Glu435, Glu444, and Trp579 in the arginine-binding pocket (Figure 2). The structures of these 1,4-substituted-triazole molecules were prepared in Discovery Studios^®^ and then docked into the prepared binding site of PRMT5 using the CDOCKER program [41]. After docking, ligand poses were analyzed, and we particularly focused on those poses that contained conformations of the scaffold portion of the molecule that aligned well with the crystal structure pose (Figure 3). The docking analysis showed that the positive region of the 1,4-triazole ring created by its strong dipole moment [42] is spatially aligned with the sulfonium portion of SAM (Figure 3). From this virtual screening, the top 13 molecules with the best docking scores were selected for synthesis and the biochemical test (**10**, **15**–**17**, and **20**–**28** in Figure 4). Additionally, 16 other triazole-adenosine analogs were also designed (Figure 4). In particular, we designed two potential bisubstrate inhibitors that have the triazole-adenosine moiety linked to a short peptide, based on the sequence of RRGRR for compound **29** and SGGGK for **30**.

### 2.2. Synthesis of the Triazole-Adenosine Inhibitors

A 5′-azido-deoxyadenosine (**1**) scaffold was synthesized in two steps according to Scheme 1 as previously reported [43]. 5′-azido-5′-deoxy-2′,3′-O-isopropylideneadenosine (**32**) was synthesized from a commercially available 2′,3′-O-isopropylideneadenosine (**31**) by a reaction under the Mitsunobu conditions with triphenylphosphine and diethyl azodicarboxylate (DEAD), and using diphenylphosphoryl azide as an azide donor in THF overnight. Purification was accomplished using silica gel chromatography (DCM:MeOH 20:1). Deprotection of the ketal group was accomplished in a 1:1 mixture of formic acid and water to yield scaffold **1** after purification using column chromatography. Diversification of **1** was performed using copper-catalyzed azide-alkyne cycloaddition (CuAAC) click chemistry [42] with various terminal alkynes and afforded the triazole compounds to be discussed in the following.

Initially, we synthesized compounds **2**–**9** from commercially available alkynes on the milligram scale as a proof-of-concept study. The synthesis first involved the preparation of a stock solution of 53 mg/mL **1** in water. A click cocktail was prepared using 12 mg of CuSO_4_ 5H_2_O and 48 mg of sodium ascorbate. To this, two equivalents of the appropriate alkyne were added, followed by the addition of 566 µL of the stock solution of **1**. These reactions completed over the course of six hours at room temperature and were subsequently purified by reverse-phase HPLC. The IC_50_ values for PRMT5 and PRMT1 were then determined (Table 1). After we observed good potencies from our first set of inhibitors, we attempted to synthesize compounds **10**–**28** by reacting compound **1** with different alkyne building blocks in a microwell-plate fashion, which can be directly subjected to biochemical assays. Each of the respective alkynes were dissolved in tBuOH or DMSO to generate 90 mM stocks. First, **1** was dissolved in water to generate a 27 mM stock. A stock click cocktail was created by dissolving CuSO_4_ and BTTAA in H_2_O to produce a solution with 9 mM stock concentrations of both. Sodium ascorbate was dissolved in H_2_O to produce a 24 mM stock. The CuSO_4_/BTTAA solution and sodium ascorbate solutions were then mixed in a 1:1 mixture to produce working concentrations of 4.5 mM for CuSO_4_ and BTTAA and 12 mM for sodium ascorbate. The alkyne, compound **1**, and the click chemistry cocktail were all mixed in a 1:1:1 volumetric ratio with a final volume of 99 μL. Controls received either solvent only, no alkyne, or no **1**. The plate was sealed and rotated on a plate shaker at 800 rpm to mix overnight. After reaction completion, the compounds were diluted using 2:1 H_2_O: tBuOH to a concentration 1 mM. This stock was further diluted to a working concentration of 50 μM using reaction buffer, which was lastly subjected to an inhibitory activity test against PRMT1 and -5 (see the Methods).

The above compounds that showed strong inhibition of PRMT5 (≥50% reduction in activity at 10 µM) in the single point well-plate assay were subsequently resynthesized in reaction vessels at the milligram scale. Of the compounds included in the well-plate screening, compounds **14**, **20**, **21**, **22**, and **24**, met this endpoint. In looking at these structures, additional compounds **11**–**13**, **15**, and **19** were also synthesized to better assess the structure–activity relationships observed from the screening. These compounds were all synthesized using the same general procedure as **2**–**9** from commercially available alkynes. For acidic alkynes **11**–**13**, the sodium salt was used, and in the case of **2** and **19**, the protected alkyne was used to generate the protected triazoles **7** and **18**, respectively.

Compounds **29** and **30** were synthesized using a solid-phase peptide synthesis protocol involving Fmoc-amino acids on Rink Amide resin. This synthesis was conducted on an AAPPTec FOCUS XC instrument. After completion of the sequence by the instrument, the resin was dried and subsequently capped using acetic anhydride to provide the N-terminal acetylated peptide. Following acetyl capping, the resin was subjected to CuAAC conditions as previously reported, with the click reaction step performed on the resin using **1** as the source of azide [44]. After the click reaction, the resin was subsequently dried, cleaved using a cleavage cocktail (see Section 4), precipitated in cold diethyl ether, centrifuged, and purified by reverse-phase HPLC.

### 2.3. Biochemical Evaluation of the Triazole-Adenosine Analogs

The methyltransferase activities of PRMT5 and PRMT1 in the absence and presence of the synthetic inhibitors were measured by using the scintillation proximity assay (SPA) protocol [45] (Table 1). In the initial test, the inhibitor concentration was set at 10 μM. H4(1–20)K20biotin [Ac-SGRGKGGKGLGKGGAKRHRK(biotin)-NH_2_] was used as the peptide substrate, at a final concentration of 0.5 μM. ^3^H-SAM was used as the methyl donor at 0.5 μM. SAH was used as a control inhibitor. Compounds that showed strong inhibition of PRMT5 in the single point well-plate assay and subsequently resynthesized at a milligram scale had their IC_50_ determined in a concentration-dependent activity assay under the same experimental condition. Analysis of the biochemical activity results against PRMT5 disclosed a few interesting structure–activity relationship (SAR) trends. Firstly, substitution of the 4-position of the triazole ring with a trimethylsilyl (TMS) group (**7**) gave a low micromolar potency of 8.3 ± 2.2 μM. Removal of the TMS group (**2**) resulted in a reduction in the potency (IC_50_ 27.0 ± 4.5 μM). The SPA test also revealed that compound **8** (R = tBu) had a similar potency to **7**, which was to be expected. Substitution with a polar functional group (-CH_2_OH, **3**) resulted in an increased potency, with IC_50_ of 4.4 ± 0.3 μM. Lengthening the substituent generally resulted in a decrease in the potency. Increasing the TMS group of **7** to –CH_2_TMS in **9** resulted in a big loss of activity (IC_50_ > 200 μM). Addition of a methylene group to **3** (i.e., **5**) or methylation of **3** to give **4** also reduced the potency. If the hydroxyl group of **3** was changed to an amine (**6**), the potency was improved; however, the selectivity was abolished. Generally, compounds that contained positive charges (**6**, **20**, **21**, **22**, **24**) near the triazole ring had further improved potencies. Compounds with negative charges varied in their potencies, with compounds that had the negative charge near the ring (**11** and **19**) having dramatically better potencies than those compounds where the charge was further from the ring (**12** and **13**). Surprisingly, bisubstrate-like compounds **29** and **30** had only modest potencies towards PRMT5, with IC_50_ of 53.7 and 45.6 μM, respectively, with only **29** showing selectivity towards PRMT5.

### 2.4. Structural Analysis for PRMT5 Inhibition

**Figure 5:**
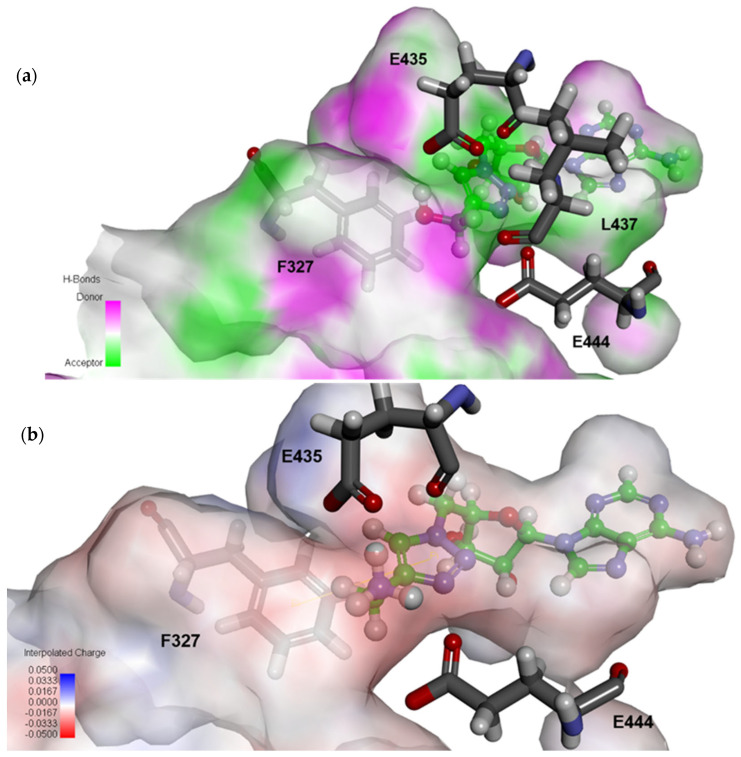
(**a**) The docked pose of **3** places the hydroxyl group in the arginine-binding pocket, where it is able to accomplish extensive hydrogen-bonding interactions. (**b**) Docked pose of **6** with the triazole portion occupying the substrate arginine-binding pocket places the positively charged substituent in a negatively charged pocket of the enzyme.

To better understand the observed structure–activity relationship (SAR) of these molecules, the previous docking analysis was further examined using Discovery Studios [46] (Figure 3). We were impressed to see that all of the compounds were predicted to form a pi–pi interaction with the target residue Phe327 with the incorporated triazole moiety. It was also revealed that substitution of the 4-position of the triazole ring generally resulted in a rotation of the triazole ring, placing the substituted group into the arginine-binding pocket. This assumed conformation of the inhibitors was important to a proper docking result. If the triazole substituent was placed into the methionine portion of the SAM-binding pocket, the docking data generally did not match the biological trends well. This rotation orientated the triazole ring such that hydrogen at the 5-position was pointed towards E444. The C5-H5 bond of the triazole aligned well with the partially positive end of the dipole moment of the triazole ring, which, when oriented in the binding pocket in this way, is capable of forming a non-classical dipolar interaction with E444 [42]. This interaction was present in the majority of triazole compounds studied. Those that did not form an interaction with E444 were **3**, **4**, **7**, and **9**, all of which had poor activities, with the exception of **3**. The observed changes in potencies can largely be attributed to how well the substituent fit into the arginine-binding pocket. More polar groups tended to do better than their less polar counterparts (i.e., **3** vs. **4**). With the hydrogen-bonding donor capability of **3**, it was able to form a stronger hydrogen bond with E444 at the hydroxyl group rather than at H5. When comparing compounds **3**, **4**, and **5**, the polar chain of **3** was able to form hydrogen bonds with E435 and E444. Increasing the length of the chain to give **5** reduced its ability to form these bonds and was only able to form bonds with E435, resulting in its loss of activity. Methylation of **3** to give **4** removed its hydrogen bond donor ability, and increased its hydrophobicity, so **4** was only able to form an H-bond to E435 (Figure 5a, Supplementary Figure S1). Bulky hydrophobic ligands tended to have reduced potencies based on their steric bulk, with bulkier groups being tolerable closer to the triazole ring (i.e., **7** vs. **9**). The aromatic group of **14** was more preferential as it also joined the triazole ring in forming a pi–pi interaction with Phe327. The loss of activity in **9** was found to be due to the bulky TMS group forming steric clashes further into the binding pocket, primarily with W579 (Supplementary Figure S2).

Comparing acidic to basic functional groups, the negative charges on acidic substituents were believed to cause poor potencies. In the case of **12** and **13**, this held true. It is also possible that the long aliphatic chains on these molecules also provided little to no interactions in the arginine-binding pocket. In the case of **11**, however, we were surprised to see that it had a potency comparable to **6**: its positive charge analog. This result is believed to be due to the ring rotating back to avoid negative interactions associated with placing the substituent in the arginine pocket such that this group is allowed to point into the methionine-binding pocket, so the acid group is able to lie in the pocket that would normally be occupied by the carboxylic acid of SAM. Lengthening the chain of **11** to give either **12** or **13** would make this rotation sterically disfavored, so the ring is forced to assume its usual conformation and have the substituent go through the arginine pocket. Of the basic substituents, the simplest substituent **6** had an activity comparable to **7**; however, it had a loss of selectivity (Table 1, Figure 5b, Supplementary Figure S3). It was shown to form extensive hydrogen bonds with both E435 and E444, similarly to the substrate arginine. Lengthening of this substituent to create **21**, **22**, and **24** also formed a similar hydrogen-bonding network, with the aromatic portions forming hydrophobic interactions with solvent-exposed residues in the binding pocket associated with the binding of substrate. For compound **19**, the positively charged amine gave similar charge interactions to **6**; however, the presence of a nearby negative charge appeared to abrogate the positive interactions, resulting in a decreased potency but with improved selectivity against PRMT1. Compound **20** was seen to be similar to **19** without the negative charge and had a greatly increased potency, providing sub-micromolar inhibition along with an approximately 49-fold selectivity for PRMT5 over PRMT1 (Supplementary Figure S4).

The bisubstrate-shaped compounds **29** and **30** were anticipated to be more potent inhibitors for PRMT5. With their surprisingly low potencies, docking analysis was also employed. Indeed, the inhibitors were shown to occupy both substrate- and cofactor-binding pockets in silico. What was observed as a potential reason for the low activity was that the linkage between the peptide portion and the triazole ring was only one methylene group. This portion was shown to occupy the arginine-binding pocket. We had previously observed that the triazole ring tends to occupy the space near F327, which is near the guanidinium portion of the substrate arginine, at least three methylene groups away from the peptide backbone. It was observed that the structure compensated for this by shifting the nucleoside portion somewhat out of its binding pocket, which resulted in reduced interactions. An additional in silico screening with compounds that had an increased linker length of two, three, and four methylene groups modeled an increase in the binding score, and a relaxation of the nucleoside portion back into its binding pocket, further supporting this hypothesis; however, additional biochemical testing would need to be conducted to confirm this trend and the behavior of **29** and **30** as proposed bisubstrate inhibitors. Their selectivity profiles were also of interest, with **30** being a non-selective inhibitor, and **29** showing great selectivity for PRMT5. This is potentially due to **30** being derived from a sequence of H4, a known substrate for both PRMT5 and PRMT1.

## 3. Materials and Methods

The organic reagents were purchased from Sigma Aldrich, TCI, and Alfa Aesar. Alkynes from the library screening were purchased from Enamine. Peptide synthesis reagents were purchased from ChemPep. An Agilent Polaris 5 C18 preparative column was used for HPLC of compounds. Detail methods for individual compound synthesis are described below. H4(1–20)K20biotin was synthesized using solid-phase peptide synthesis method.

### 3.1. Synthesis

*5′-azido-5′-deoxy-2′,3′-O-isopropylideneadenosine* (**32**)*.* In total, 1.49 g of triphenylphosphine (5.7 mmol) was dissolved in 5 mL of THF. The solution was placed on an ice bath and stirred for 10 min. In total, 3 mL of DEAD (40% in toluene) was added dropwise to the triphenylphosphine solution, and the resulting yellow solution was stirred for another 10 min. Then, 1 mL of DPPA (5.7 mmol) was added dropwise over 1 min to the solution, and the solution was removed from ice and allowed to stir at room temperature for 10 min. In total, 500 mg of 5′-deoxy-2′,3′-O-isopropylideneadenosine (1.63 mmol) was added portion-wise to the solution. The flask was then stirred overnight at room temperature. After completion, the solution was diluted with 2 mL of ethyl acetate and concentrated to afford a yellow oil. The oil was then purified through column chromatography (50–100% ethyl acetate in hexanes) to give a clear oil. Yield: 500 mg, 93%. ESI-HRMS calc for C_13_H_16_N_8_O_3_ [M+H]^+^: 333.1419 found 333.1398 ^1^H NMR (400 MHz, DMSO-*d*6) δ 8.35 (s, 1H), 8.19 (s, 1H), 7.38 (s, 2H), 6.22 (d, *J* = 2.6 Hz, 1H), 5.53 (dd, *J* = 6.2, 2.7 Hz, 1H), 5.01 (dd, *J* = 6.2, 3.0 Hz, 1H), 4.31 (ddd, *J* = 7.4, 4.8, 3.0 Hz, 1H), 3.67–3.50 (m, 2H), 1.55 (s, 3H), 1.34 (s, 3H). 13C NMR (101 MHz, DMSO) δ 156.64, 153.27, 149.23, 140.49, 119.62, 114.00, 89.54, 84.98, 83.35, 81.99, 51.96, 27.43, 25.63.

*5′-azido-5′-deoxyadenosine* (**1**)*.* In total, 500 mg of **32** (1.71 mmol) was dissolved in 9 mL of a 1:8 mixture of water and formic acid. This solution was stirred at room temperature overnight. The reaction progress was monitored via analytical HPLC (3–30% gradient CH_3_CN in H_2_O over 20 min, r.t. 15 min). The compound was purified via column chromatography (1–10% methanol in ethyl acetate). Yield: 281 mg, 64%. ESI-HRMS calc for C_10_H_12_N_8_O_3_ [M+H]^+^: 293.1106, found 293.1101 ^1^H NMR (400 MHz, DMSO-*d*_6_) δ 8.37 (s, 1H), 8.19 (s, 1H), 7.32 (s, 2H), 5.95 (d, *J* = 5.4 Hz, 1H), 5.61 (br s, 1H), 5.41 (br s, 1H), 4.77 (t, *J* = 5.3 Hz, 1H), 4.23 (t, *J* = 4.7 Hz, 1H), 4.08–4.00 (m, 1H), 3.74–3.52 (m, 2H). ^13^C NMR (101 MHz, DMSO) δ 156.55, 153.14, 149.86, 140.41, 119.67, 88.25, 83.42, 73.20, 71.39, 52.19.

### 3.2. General Click Reaction for Hit Validation of Select Compounds

In total, 42 mg of sodium ascorbate (0.21 mmol) and 12 mg of CuSO_4_·5H_2_O (0.05 mmol) were dissolved in 1 mL of water. In total, 566 µL of a 53 mg/mL solution of **1** (30 mg, 0.1 mmol) was added to the solution, followed by 2 equivalents of the appropriate alkyne. The reaction mixture was stirred at room temperature for 6 h. After reaction completion, the compounds were purified using reverse-phase HPLC (5–50% gradient CH_3_CN in H_2_O over 45 min) and lyophilized to give the final products as solids. For the hydrophobic alkynes used to synthesize **18**, **21**, and **24**, a solvent system of 2:1 DMSO:H_2_O was used. For the acidic alkynes used to synthesize **11**–**13**, the associated sodium salt was used.

*(2R,3S,4R,5R)-2-((1H-1,2,3-triazol-1-yl)methyl)-5-(6-amino-9H-purin-9-yl)tetrahydrofuran-3,4-diol* (**2**). In total, 22.5 mg (0.06 mmol) of **7** was dissolved in 1 mL of THF. In total, 115 µL of a 1M solution of TBAF in THF (0.12 mmol) was added to the solution, and it was stirred at room temperature overnight. The following day, the solution was evaporated, and the resulting residue was purified by reverse-phase HPLC and lyophilized to give a white powder. Yield: 15 mg, 82% ESI-HRMS calc for C_12_H_15_N_8_O_3_ [M+H]^+^: 319.1262 found 319.1252 ^1^H NMR (400 MHz, DMSO-*d*_6_) δ 8.46 (s, 1H), 8.36 (s, 1H), 8.02 (s, 1H), 7.68 (s, 1H), 5.95 (d, *J* = 5.4 Hz, 1H), 4.87–4.73 (m, 2H), 4.63 (t, *J* = 5.2 Hz, 1H), 4.34–4.21 (m, 2H).

*(2R,3R,4S,5R)-2-(6-amino-9H-purin-9-yl)-5-((4-(hydroxymethyl)-1H-1,2,3-triazol-1-yl)methyl)tetrahydrofuran-3,4-diol* (**3**). Synthesized using the general procedure detailed above. Yield: 17 mg, 48% ESI-HRMS calc for C_13_H_17_N_8_O_4_ [M+H]^+^: 349.1368 found 349.1362 ^1^H NMR (400 MHz, DMSO-*d*_6_) δ 8.53 (s, 1H), 8.39 (s, 1H), 7.85 (s, 1H), 5.96 (d, *J* = 5.3 Hz, 1H), 4.74 (d, *J* = 7.4 Hz, 2H), 4.60 (d, *J* = 5.3 Hz, 1H), 4.46 (s, 2H).

*(2R,3R,4S,5R)-2-(6-amino-9H-purin-9-yl)-5-((4-(methoxymethyl)-1H-1,2,3-triazol-1-yl)methyl)tetrahydrofuran-3,4-diol* (**4**). Synthesized using the general procedure detailed above. Yield 21 mg, 56% ESI-HRMS calc for C_14_H_19_N_8_O_4_ [M+H]^+^: 363.1524 found 363.1514 ^1^H NMR (400 MHz, Methanol-*d*_4_) δ 8.37 (s, 2H), 7.94 (s, 1H), 6.05 (s, 1H), 4.62 (s, 1H), 4.51 (s, 1H), 4.42 (s, 2H), 3.38 (s, 3H).

*(2R,3R,4S,5R)-2-(6-amino-9H-purin-9-yl)-5-((4-(2-hydroxyethyl)-1H-1,2,3-triazol-1-yl)methyl)tetrahydrofuran-3,4-diol* (**5**). Synthesized using the general procedure detailed above. Yield: 16 mg, 43% ESI-HRMS calc for C_14_H_19_N_8_O_4_ [M+H]^+^: 363.1524 found 363.1519 ^1^H NMR (400 MHz, DMSO-*d*_6_) δ 8.47 (d, *J* = 8.1 Hz, 1H), 8.39 (s, 1H), 7.77 (s, 1H), 7.70 (s, 1H), 5.90 (d, *J* = 5.1 Hz, 1H), 3.53 (t, *J* = 6.9 Hz, 1H), 2.98 (t, *J* = 6.6 Hz, 1H), 2.65 (t, *J* = 6.9 Hz, 1H).

*(2R,3R,4S,5R)-2-(6-amino-9H-purin-9-yl)-5-((4-(aminomethyl)-1H-1,2,3-triazol-1-yl)methyl)tetrahydrofuran-3,4-diol* (**6**). Synthesized using the general procedure detailed above. Yield: 21 mg, 59% ESI-HRMS calc for C_13_H_19_N_9_O_3_ [M+H]^+^: 348.1528 found 348.1524 ^1^H NMR (400 MHz, DMSO-*d*_6_) δ 8.54 (s, 1H), 8.38 (s, 1H), 8.06 (s, 1H), 6.01 (d, *J* = 5.4 Hz, 1H), 4.90 (d, *J* = 5.5 Hz, 2H), 4.77 (t, *J* = 4.9 Hz, 1H).

*(2R,3R,4S,5R)-2-(6-amino-9H-purin-9-yl)-5-((4-(trimethylsilyl)-1H-1,2,3-triazol-1-yl)methyl)tetrahydrofuran-3,4-diol* (**7**). Synthesized using the general procedure detailed above. Yield: 25 mg, 62% ESI-HRMS calc for C_15_H_23_N_8_O_3_Si [M+H]^+^: 391.1657 found 391.6566 ^1^H NMR (400 MHz, DMSO-*d*_6_) δ 8.06 (s, 1H), 5.94 (d, *J* = 5.0 Hz, 1H), 4.77 (d, *J* = 4.4 Hz, 3H), 4.52 (d, *J* = 5.1 Hz, 2H), 0.21 (d, *J* = 2.5 Hz, 9H).

*(2R,3R,4S,5R)-2-(6-amino-9H-purin-9-yl)-5-((4-(tert-butyl)-1H-1,2,3-triazol-1-yl)methyl)tetrahydrofuran-3,4-diol* (**8**). Synthesized using the general procedure detailed above. Yield: 23 mg. 60% ESI-HRMS calc for C_16_H_23_N_8_O_3_ [M+H]^+^: 375.1888 found 375.1876 ^1^H NMR (400 MHz, DMSO-*d*_6_) δ 8.60 (s, 1H), 5.98 (d, *J* = 5.3 Hz, 1H), 4.70 (t, *J* = 5.2 Hz, 1H), 4.20 (t, *J* = 4.7 Hz, 3H), 4.08 (dt, *J* = 7.4, 3.9 Hz, 3H), 3.74–3.52 (m, 2H).

*(2R,3R,4S,5R)-2-(6-amino-9H-purin-9-yl)-5-((4-((trimethylsilyl)methyl)-1H-1,2,3-triazol-1-yl)methyl)tetrahydrofuran-3,4-diol* (**9**). Synthesized using the general procedure detailed above. Yield: 26 mg, 63% ESI-HRMS calc for C_16_H_25_N_8_O_3_Si [M+H]^+^: 405.1814 found 405.1806 ^1^H NMR (400 MHz, Methanol-*d*_4_) δ 8.47 (br s, 2H), 7.75 (br s, 1H), 6.08 (s, 1H), 4.82 (s, 2H), 4.69 (s, 1H), 4.45 (s, 2H), 2.07 (br s, 2H), 0.00 (s, 9H).

*1-(((2R,3S,4R,5R)-5-(6-amino-9H-purin-9-yl)-3,4-dihydroxytetrahydrofuran-2-yl)methyl)-1H-1,2,3-triazole-4-carboxylic acid* (**11**). Synthesized using the general procedure detailed above. Yield: 15 mg, 40% ESI-HRMS calc for C_13_H_15_N_8_O_5_ [M+H]^+^: 363.1160 found 363.1161 ^1^H NMR (400 MHz, DMSO-*d*_6_) δ 8.59 (s, 1H), 8.38 (s, 1H), 5.97 (d, *J* = 5.3 Hz, 1H), 4.70 (t, *J* = 5.2 Hz, 1H), 4.19 (t, *J* = 4.7 Hz, 2H), 4.08 (dt, *J* = 7.4, 3.9 Hz, 2H), 3.69–3.57 (m, 3H). ^13^C NMR (101 MHz, DMSO) δ 154.44, 150.44, 141.39, 130.70, 88.31, 83.56, 73.40, 71.32, 52.16.

*4-(1-(((2R,3S,4R,5R)-5-(6-amino-9H-purin-9-yl)-3,4-dihydroxytetrahydrofuran-2-yl)methyl)-1H-1,2,3-triazol-4-yl)butanoic acid* (**12**). Synthesized using the general procedure detailed above. Yield: 20 mg, 48% ESI-HRMS calc for C_16_H_21_N_8_O_5_ [M+H]^+^: 405.1630 found 405.1629 ^1^H NMR (400 MHz, Methanol-*d*_4_) δ 6.07 (s, 1H), 4.65 (s, 1H), 4.44 (s, 2H), 2.68 (br s, 2H), 2.34 (s, 2H), 1.91 (s, 2H).

*5-(1-(((2R,3S,4R,5R)-5-(6-amino-9H-purin-9-yl)-3,4-dihydroxytetrahydrofuran-2-yl)methyl)-1H-1,2,3-triazol-4-yl)pentanoic acid* (**13**). Synthesized using the general procedure detailed above. Yield: 25 mg, 58% ESI-HRMS calc for C_17_H_23_N_8_O_5_ [M+H]^+^: 419.1786 found 419.1788 ^1^H NMR (400 MHz, Methanol-*d*_4_) δ 6.03 (s, 1H), 4.61 (s, 1H), 4.42 (s, 2H), 2.33 (d, *J* = 7.5 Hz, 3H), 2.19 (dd, *J* = 15.7, 8.5 Hz, 1H), 1.74–1.45 (m, 5H).

*(2R,3R,4S,5R)-2-(6-amino-9H-purin-9-yl)-5-((4-phenyl-1H-1,2,3-triazol-1-yl)methyl)tetrahydrofuran-3,4-diol* (**14**). Synthesized using the general procedure detailed above. Yield: 24 mg, 59% ESI-HRMS calc for C_18_H_19_N_8_O_3_ [M+H]^+^: 395.1575 found 395.1575 ^1^H NMR (400 MHz, Methanol-*d*_4_) δ 8.03 (s, 1H), 7.62 (d, *J* = 7.4 Hz, 2H), 7.49–7.32 (m, 3H), 6.05 (d, *J* = 3.0 Hz, 1H), 4.64 (dd, *J* = 23.7, 5.9 Hz, 2H), 4.46 (t, *J* = 4.8 Hz, 1H).^13^C NMR (101 MHz, DMSO) δ 146.77, 131.09, 129.34, 128.32, 125.59, 122.60, 88.42, 82.80, 73.25, 71.34, 51.96.

*N-((1-(((2R,3S,4R,5R)-5-(6-amino-9H-purin-9-yl)-3,4-dihydroxytetrahydrofuran-2-yl)methyl)-1H-1,2,3-triazol-4-yl)methyl)benzamide* (**15**). Synthesized using the general procedure detailed above. Yield: 27 mg, 58% ESI-HRMS calc for C_20_H_22_N_9_O_4_ [M+H]^+^: 452.1790 found 452.1769 ^1^H NMR (400 MHz, DMSO-d_6_) δ 8.99 (t, *J* = 5.8 Hz, 1H), 8.54 (s, 1H), 8.39 (s, 1H), 7.89 (s, 1H), 7.85–7.80 (m, 2H), 7.58–7.48 (m, 1H), 7.45 (t, *J* = 7.3 Hz, 2H), 5.95 (d, *J* = 5.3 Hz, 1H), 4.74 (dd, *J* = 7.7, 6.0 Hz, 2H), 4.62 (t, *J* = 5.2 Hz, 1H), 4.46 (d, *J* = 5.7 Hz, 2H), 4.30 (dt, *J* = 8.2, 4.4 Hz, 1H), 4.24 (t, *J* = 4.5 Hz, 1H).

*(S)-2-((((9H-fluoren-9-yl)methoxy)carbonyl)amino)-3-(1-(((2R,3S,4R,5R)-5-(6-amino-9H-purin-9-yl)-3,4-dihydroxytetrahydrofuran-2-yl)methyl)-1H-1,2,3-triazol-4-yl)propanoic acid* (**18**). Synthesized using the general procedure detailed above. Yield: 20 mg, 31% MALDI-MS calc for C_30_H_30_N_9_O_7_ [M+H]^+^: 628.23 found 628.5 ^1^H NMR (400 MHz, DMSO-d_6_) δ 7.88 (d, *J* = 7.4 Hz, 2H), 7.75 (s, 1H), 7.66 (dt, *J* = 6.9, 3.4 Hz, 2H), 7.41 (t, *J* = 7.4 Hz, 2H), 7.30 (td, *J* = 7.5, 2.5 Hz, 2H), 5.94 (d, *J* = 5.1 Hz, 1H), 4.72 (d, *J* = 5.6 Hz, 2H), 4.59 (d, *J* = 5.0 Hz, 1H), 4.23 (ddd, *J* = 18.4, 15.1, 5.8 Hz, 6H).

*(S)-2-amino-3-(1-(((2R,3S,4R,5R)-5-(6-amino-9H-purin-9-yl)-3,4-dihydroxytetrahydrofuran-2-yl)methyl)-1H-1,2,3-triazol-4-yl)propanoic acid* (**19**). In total, 15 mg (0.024 mmol) of **18** was dissolved in 2 mL of a 20% solution of piperidine in THF. The solution was stirred at room temperature for 90 min at which point the solution was concentrated and the resulting residue was purified by reverse-phase HPLC and lyophilized to give a white powder. Yield: 7 mg, 72% ESI-HRMS calc for C_15_H_20_N_9_O_5_ [M+H]^+^: 406.1582 found 406.1568 ^1^H NMR (400 MHz, DMSO-d_6_) δ 8.36 (s, 1H), 8.23 (s, 3H), 7.82 (s, 1H), 5.93 (d, *J* = 5.1 Hz, 1H), 4.82–4.74 (m, 2H), 4.68 (t, *J* = 4.8 Hz, 1H), 4.26 (dd, *J* = 14.2, 9.0 Hz, 3H).

*(1-(((2R,3S,4R,5R)-5-(6-amino-9H-purin-9-yl)-3,4-dihydroxytetrahydrofuran-2-yl)methyl)-1H-1,2,3-triazol-4-yl)methyl (tert-butoxycarbonyl)-L-valinate* (Boc-**20**). Synthesized using the general procedure detailed above. Yield: 25 mg, 44% ESI-HRMS calc for C_23_H_34_N_9_O_7_ [M+H]^+^: 548.2576 found 548.2587 ^1^H NMR (400 MHz, Methanol-d_4_) δ 6.03 (s, 1H), 4.59 (s, 1H), 4.40 (s, 2H), 4.03 (d, *J* = 5.3 Hz, 1H), 2.02 (d, *J* = 2.1 Hz, 4H), 1.50–1.22 (m, 12H), 0.83 (s, 7H).

*(1-(((2R,3S,4R,5R)-5-(6-amino-9H-purin-9-yl)-3,4-dihydroxytetrahydrofuran-2-yl)methyl)-1H-1,2,3-triazol-4-yl)methyl L-valinate* (**20**). In total, 12 mg of Boc-protected **20** was dissolved in 2 mL of a 9:1 TFA:MeOH solution and was stirred at room temperature for 4 h. The TFA was removed using rotary evaporation and the resulting residue was purified using reverse-phase HPLC and lyophilized to give a white powder. Yield: 8 mg, 82% ESI-HRMS calc for C_18_H_26_N_9_O_5_ [M+H]^+^: 448.2052 found 448.2045 ^1^H NMR (400 MHz, DMSO-*d*_6_) δ 8.46 (s, 1H), 8.33 (d, *J* = 4.9 Hz, 3H), 8.08 (s, 1H), 5.94 (d, *J* = 5.3 Hz, 1H), 5.31–5.21 (m, 2H), 4.81 (d, *J* = 5.6 Hz, 2H), 4.66 (t, *J* = 5.0 Hz, 1H), 4.28 (td, *J* = 8.2, 7.1, 3.2 Hz, 2H), 0.87 (dd, *J* = 9.9, 6.9 Hz, 6H).^13^C NMR (101 MHz, DMSO) δ 141.32, 126.29, 88.34, 82.91, 73.08, 71.45, 58.87, 52.03, 29.89, 18.45, 17.87.

*2-(((1-(((2R,3S,4R,5R)-5-(6-amino-9H-purin-9-yl)-3,4-dihydroxytetrahydrofuran-2-yl)methyl)-1H-1,2,3-triazol-4-yl)methyl)amino)-N-(m-tolyl)acetamide* (**21**). Synthesized using the general procedure detailed above. Yield: 16 mg, 31% ESI-HRMS calc for C_22_H_27_N_10_O_4_ [M+H]^+^: 495.2212 found 495.2205 ^1^H NMR (400 MHz, DMSO-d_6_) δ 10.36 (s, 1H), 8.36 (s, 1H), 8.21 (s, 1H), 8.06 (s, 1H), 7.36 (d, *J* = 6.9 Hz, 2H), 7.26–7.16 (m, 1H), 6.94 (d, *J* = 7.6 Hz, 1H), 5.94 (d, *J* = 5.4 Hz, 1H), 4.90–4.84 (m, 1H), 4.75 (t, *J* = 5.1 Hz, 1H), 4.29 (d, *J* = 3.5 Hz, 3H), 3.94 (s, 2H).

*Methyl 4-((((1-(((2R,3S,4R,5R)-5-(6-amino-9H-purin-9-yl)-3,4-dihydroxytetrahydrofuran-2-yl)methyl)-1H-1,2,3-triazol-4-yl)methyl)amino)methyl)benzoate* (**22**). Synthesized using the general procedure detailed above. Yield: 15 mg, 30% ESI-HRMS calc for C_22_H_26_N_9_O_5_ [M+H]^+^: 496.2052 found 496.2054 ^1^H NMR (400 MHz, DMSO-d6) δ 8.45–8.36 (m, 3H), 8.23 (s, 1H), 7.98 (s, 1H), 5.93 (d, *J* = 5.5 Hz, 1H), 4.96–4.80 (m, 2H), 4.74 (t, *J* = 5.1 Hz, 1H), 4.28 (dq, *J* = 7.0, 4.2 Hz, 2H), 4.07 (q, *J* = 5.8 Hz, 2H).

*2-(((1-(((2R,3S,4R,5R)-5-(6-amino-9H-purin-9-yl)-3,4-dihydroxytetrahydrofuran-2-yl)methyl)-1H-1,2,3-triazol-4-yl)methyl)amino)-N-(2-fluorophenyl)acetamide* (**24**). Synthesized using the general procedure detailed above. Yield: 19 mg, 37% ESI-HRMS calc for C_21_H_24_FN_10_O_4_ [M+H]^+^: 499.1961 found 499.1962 ^1^H NMR (400 MHz, DMSO-d6) δ 10.33 (s, 1H), 8.46 (s, 1H), 8.29 (s, 1H), 8.07 (s, 1H), 7.99–7.84 (m, 2H), 7.34–7.18 (m, 2H), 5.95 (d, *J* = 5.4 Hz, 1H), 4.86 (d, *J* = 5.2 Hz, 1H), 4.72 (t, *J* = 4.9 Hz, 1H), 4.34–4.23 (m, 3H), 4.03 (s, 2H).

### 3.3. Solid-Phase Peptide Synthesis of **29** and **30**

Synthesis of **29** and **30** was conducted using the FOCUS XC instrument from AAPPTec. Prior to synthesis, Fmoc-amino acids were dissolved in N-methyl-2-pyrrolidone to create 0.2 M stocks. Rink amide resin, Fmoc-amino acids, and other reagents were added to the appropriate reaction vials. Resin was swollen in 15 mL of DMF and mixed using nitrogen gas for 10 min. After draining of the solvent, deprotection was conducted using 8 mL of 20% piperidine in DMF. The deprotection reaction was mixed for 30 min, drained, and repeated with another 8 mL of 20% piperidine in DMF. The resin was drained and washed with 15 mL of DMF. Prior to coupling, 5 mL of 0.2 M HCTU in DMF, 5 mL of 22% N-methyl morpholine in DMF, and 5 mL of the appropriate amino acid were mixed and added to the reaction vessel. Coupling was conducted using nitrogen gas mixing for 1 h after which the solution was drained, and resin was washed with 15 mL of DMF. For **30**, this coupling step was repeated for each amino acid addition. Following coupling, the cycles of deprotection and coupling were conducted until the sequence was complete and the final N-terminus was deprotected. Acetyl capping was conducted by the addition of 4 mL of DMF and 1 mL of acetic anhydride. The resin was mixed mechanically for 5 min; then, 1.5 mL of DIPEA was added and the resin continued to be mixed for an additional 30 min. The resin was then washed using DMF and dried using DCM.

On-resin click reactions were conducted first by swelling the resin in 7 mL of DMSO for 10 min. Stock solutions of click reagents were made in separate vials by dissolving 7.17 mg of CuBr was in 1.5 mL of DMSO in the first vial, 14.6 mg of **1** in 1 mL of DMSO in the second vial, and 9.91 mg of sodium ascorbate in 0.45 mL of water in the third vial. After swelling, the resin was drained and the solutions of CuBr and **1** were added to the resin followed by 58.2 μL of 2,6-lutidine, 86.5 μL of DIPEA, and lastly the solution of sodium ascorbate. The reaction was allowed to mix overnight. The following morning, the resin was drained; washed with DMSO, water, and DCM; and dried. Cleavage was conducted using a cocktail containing 200 mg of phenol, 0.2 mL of water, 0.2 mL of thioanisole, 0.1 mL of 1,2-ethanedithiol, 40 μL of triisopropylsilane, and 3.46 mL of trifluoroacetic acid and mixing for 4 h. After cleavage, the peptide was precipitated in 8 mL of diethyl ether and centrifuged at 5000 rpm at 4 °C for 10 min. The supernatant was removed and pellet dried. The peptide was then purified using reverse-phase HPLC and lyophilized to obtain the product. MALDI-MS calc for compound **29**: C_41_H_70_N_26_O_9_ [M]^+^: 1070.6, found 1071.7. MALDI-MS calc for compound **30**: C_30_H_45_N_15_O_10_ [M]^+^: 775.3, found 776.2.

### 3.4. PRMT5 Expression and Purification

Human recombinant His-tag PRMT5 was co-expressed with His-tag MEP50 using the Bac-to-Bac baculovirus expression system (Invitrogen, Life Technologies, Carlsbad, CA, USA) as previously described [47]. Briefly, pFB-LIC-Bse-PRMT5 and pFB-LIC-Bse-MEP50 were heat shock transformed in the DH10Bac *E. coli* to obtain the recombinant bacmids, and the bacmids were extracted using the PureLink HiPure Plasmid Miniprep Kit (Invitrogen, Thermo Scientific, Carlsbad, CA, USA). The purity and concentration of the plasmid were measured by a Nanodrop. Sf9 cells were cultured in Sf-900 II SFM medium until the cell density reached 1.5 × 10^6^ cells/mL and cell viability was more than 95%. Cells were plated in a 6-well tissue culture plate at a density of 4 × 10^5^ cells/mL per well. In total, 2 µg of PRMT5 and MEP50 recombinant bacmid were transfected into each well of cells. After 5 h of transfection, the transfection medium was replaced by 2 mL of Sf-900 II SFM + 100 U P/S per well. The medium was collected and centrifuged at 1000 rpm for 5 min to obtain the supernatant P1 viral stock after 7 days of inoculation at 27.5 °C. To obtain a higher titer of viral stock, P1 viral stock was amplified in a 6-well tissue culture plate containing 2 × 10^6^ cells per well. In total, 0.1 MOI of P1 virus stock was added into each well. After 7 days of incubation at 27.5 °C, the cell medium was centrifuged at 1000 rpm for 5 min to obtain the P2 viral stock in the supernatant. The Baculovirus plaque assay was performed to determine the viral titer concentration; the target concentration range was 1 × 10^7^–1 × 10^8^ IFU/mL.

For the expression of PRMT5 and MEP50, Sf9 insect cells were cultured in suspension at 27.5 °C at a speed of 130 rpm in Expression Systems ESF 921 medium with 100 U P/S. In total, 200 mL of Sf9 cell culture was inoculated with PRMT5 and MEP50 P2 viral stocks when the cell density reached 1.5 × 10^6^ cells/mL. After incubation for 72 h at 27.5 °C, the cells were harvested and centrifuged. PRMT5 and MEP50 protein were collected in the cell pellet for further purification. Cell pellets were disrupted twice at 100 psi in 25 mL of cell lysis buffer (50 mM Tris pH 8, 300 mM NaCl, 10% glycerol, 0.1% Triton X-100, 1 mM PMSF). Before the Ni-NTA purification, 10 mL of Ni-NTA resin (EMD Millipore, Burlington, MA, USA) was loaded into a 50 mL column and equilibrated with equilibration buffer (50 mM Tris pH 8, 250 mM NaCl, 30 mM Imidazole, 10% glycerol, 1 mM TCEP, 1 mM PMSF). After 60 min of cell lysate centrifugation at 4 °C at 18,000 rpm, the cell lysate supernatant was incubated with the equilibrated nickel resin. The resin was washed with equilibration buffer and protein was eluted with elution buffer (50 mM Tris pH 8, 250 mM NaCl, 300 mM Imidazole, 10% glycerol, 1 mM TCEP, 1 mM PMSF). For the dialysis of protein, elution samples were loaded into a 10,000 MWCO SnakeSkin (Thermo Scientific, Waltham, MA, USA) dialysis bag in the storage buffer (50 mM Tris pH 8, 250 mM NaCl, 10% glycerol, 1 mM TCEP). Afterwards, proteins were concentrated using 10,000 MWCO Vivaspin 20 ultrafiltration devices (GE Healthcare, Chicago, IL, USA) by centrifugation at 6000 rcf, 4 °C. Bradford assay was carried out to determine the concentration of PRMT5/MEP50 protein. Purified protein was verified by running an SDS-PAGE.

### 3.5. Biological Activity Assays

Inhibitor was dissolved in water to create a 10 mM stock concentration. This was then diluted in buffer (50 mM HEPES pH 8, 10 mM NaCl, 0.5 mM EDTA, 0.5 mM DTT in H_2_O) to create a series of working concentrations centered around an anticipated IC_50_. [H]^3^-SAM and substrate were diluted together in buffer to working concentrations of 2.5 and 5 μM, respectively. To each well in a 96-well plate, 6 μL of the inhibitor solution was added, with negative and positive controls receiving buffer. In total, 6 μL of the [H]^3^-SAM/substrate solution was then added to each well. The enzyme was then diluted in buffer to a working concentration of 0.83 μM, and 18 μL was added to each well, with negative controls receiving 18 μL of buffer. The plate was covered, and the reaction was allowed to run for 30 min and was then quenched with the addition of 30 μL of 100% iPrOH followed by 60 μL of 50% iPrOH in H_2_O. In total, 10 μL of 20 mg/mL streptavidin-coated scintillant beads were added and the plate was read using a MicroBeta (Perkin Elmer, Waltham, MA, USA) plate reader. The CPM counts in the absence of an inhibitor for each data set were considered as 100% activity. In the absence of the enzyme and inhibitor, the CPM counts in each data set were defined as background (0%).

### 3.6. In Silico Docking of Ligands to PRMT5

CDOCKER from Discovery Studio (version 4.0) was used to predict the binding poses of the synthesized compounds **2**–**30** in the active site of PRMT5. The protein structure was obtained from the Protein Data Bank (PDB ID: 4GQB). Ligands were prepared using the Prepare Ligands module and ionization states were generated at pH 7.0 ± 1.5. Protein structure was prepared with Prepare Protein using the module within Discovery Studios and energy minimized using the CHARMM force field. Binding sites were determined using PDB records and analysis of the enzyme cavities forming a sphere centered at the active site with a radius of 11.6 Å. A series of conformations of the ligands were generated by using molecular dynamics with various random seeds. Each of the conformations was translated through the active site using flexible ligand docking. The binding affinities were predicted using the resulting CDOCKER score. Each ligand was screened for the formation of crucial interactions on the nucleoside portion of the ligand and optimum poses were saved.

## 4. Conclusions

In this study, we developed a modular, easily diversifiable set of chemical probes for selective targeting of PRMT5. We demonstrated that triazole-containing adenosine analogs have the capability of selectively targeting PRMT5 over the type-I enzyme PRMT1. Through the use of CuAAC click chemistry, our scaffold can be linked to a subset of molecules to create a wide array of potential inhibitors containing the targeting moiety. Our SAR analysis showed that the triazole ring is positioned well to form a pi–pi interaction with the conserved residue in PRMT5: Phe327, and allows for substituents at the C4 position to easily access the arginine-binding pocket. With these results, it is reasonable to conclude that this targeting scaffold can be potentially used to selectively target PRMT5 and open the door to potentially a new class of bisubstrate inhibitors for PRMT5. Future directions for this scaffold could involve a thorough investigation into the selective mechanisms behind the triazole ring and further diversification of the C4 and C5 positions to provide targeted bisubstrate inhibitors of PRMT5.

## Figures and Tables

**Table 1:** Activities of analogs measured in the single point SPA test at 10 μM against PRMT5 and PRMT1, with activities reported as the remaining activity of the enzyme in a percentage. IC_50_ (if determined) is listed in parentheses.

Name	Relative Activity of PRMT5 at 10 μM (IC_50_)	Relative Activity of PRMT1 at 10 μM (IC_50_)
**1**	48.0 ± 9.7% (3.0 ± 0.3 μM)	97.5 ± 7.6% (>1000 μM)
**2**	(27.0 ± 4.5 μM)	(>1000 μM)
**3**	(4.4 ± 0.3 μM)	(>1000 μM)
**4**	(143.9 ± 8.4 μM)	(>1000 μM)
**5**	(194.9 ± 26.2 μM)	(>1000 μM)
**6**	61.0 ± 8.0% (1.2 ± 0.2 µM)	96.3 ± 8.0% (7.83 ± 8.4 μM)
**7**	(8.3 ± 2.2 μM)	(>1000 μM)
**8**	(7.7 ± 3.9 μM)	(>1000 μM)
**9**	(>200 μM)	(>1000 μM)
**10**	71.0 ± 17.0%	102.6 ± 5.6%
**11**	74.0 ± 11.0%(3.15 ± 0.11 µM)	96.9 ± 2.2%
**12**	73 ± 12.0% (>400 µM)	99.3 ± 6.7%
**13**	57.0 ± 4.4% (>40 µM)	92.7 ± 10.0%
**14**	51.0 ± 5.7% (2.6 ± 0.1 µM)	90.1 ± 5.7%
**15**	94.2 ± 12.0% (>40 µM)	123.0 ± 4.4%
**16**	80.0 ± 9.5%	101.4 ± 10.0%
**17**	56.0 ± 10.0%	91.0 ± 11.0%
**18**	56.0 ± 6.4%	91.2 ± 4.7%
**19**	(18.9 ± 3.0 μM)	(>200 μM)
**20**	26.0 ± 0.45% (0.88 ± 0.09 µM)	111.0 ± 5.5% (43.5 ± 11.0 μM)
**21**	32.0 ± 3.3%	84 ± 55.0%
**22**	30.0 ± 1.7% (1.3 ± 0.2 µM)	94.2 ± 2.2% (129.6 ± 38.7 μM)
**23**	70.0 ± 5.6%	91.8 ± 16.0%
**24**	40.0 ± 3.6% (13.2 ± 3.0 µM)	93.0 ± 1.3%
**25**	72.0 ± 9.6%	99.1 ± 13.0%
**26**	83.0 ± 2.5%	100.1 ± 4.6%
**27**	40.0 ± 3.4%	50.0 ± 9.9%
**28**	76.0 ± 11.0%	77.0 ± 23.0%
**29**	(53.7 ± 6.5 μM)	(>400 μM)
**30**	(45.6 ± 8.7 μM)	(46.1 ± 18.0 μM)

## Data Availability

Not applicable.

## Supplementary Materials

The following supporting information can be downloaded at: https://www.mdpi.com/article/10.3390/molecules27123779/s1, Figure S1: Docked poses of 3 (top) and 4 (bottom) overlayed with sinefungin (green) and a substrate arginine (green). To the right is shown a 2D representation of the interactions formed by the representative compounds; Figure S2: Docked pose of 9 overlayed with sinefungin (green) and a substrate arginine (green). To the right is shown a 2D representation of the interactions formed by the representative compound; Figure S3: Docked pose of 6 overlayed with sinefungin (green) and a substrate arginine (green). To the right is shown a 2D representation of the interactions formed by the representative compound; Figure S4. Docked poses of 20 (top) and 19 (bottom) overlayed with sinefungin (green) and a substrate arginine (green). To the right is shown a 2D representation of the interactions formed by the representative compounds.

## Sample Availability

Samples of the tested compounds may be available from the authors upon request.
