# Supplementary material for: Synthesis and Activity of Triazole-Adenosine Analogs as Protein Arginine Methyltransferase 5 Inhibitors
Source: Molecules. 2022 Jun 11;27(12):3779. doi: 10.3390/molecules27123779 (PMC9228412; doi:10.3390/molecules27123779)
Supplement: Supplementary file 1 [file molecules-27-03779-s001.zip › Supplementary docking figures.pdf]

Synthesis and Activity of Triazole-Containing Adenosine Analogs as Protein Arginine Methyltransferase 5 Inhibitors

Tyler Brown, Mengtong Cao, Y. George Zheng

Department of Pharmaceutical and Biomedical Sciences, College of Pharmacy, The University of Georgia, Athens, Georgia 30602, United States.

Supplementary Information

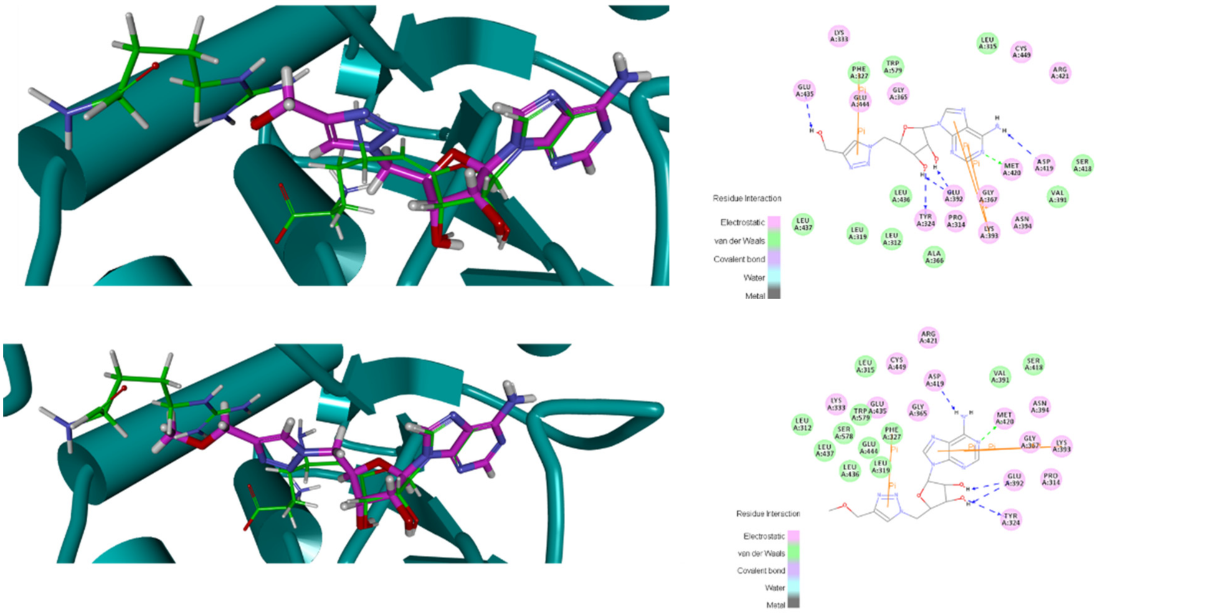

Figure S1: Docked poses of 3 (top) and 4 (bottom) overlayed with sinefungin (green) and a substrate arginine (green). To the right is shown a 2D representation of the interactions formed by the representative compounds.

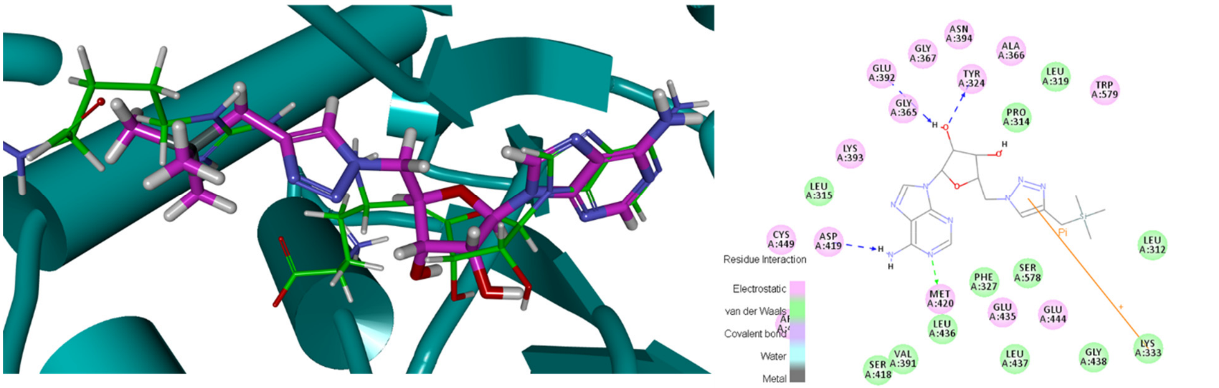

Figure S2: Docked pose of 9 overlayed with sinefungin (green) and a substrate arginine (green). To the right is shown a 2D representation of the interactions formed by the representative compound.

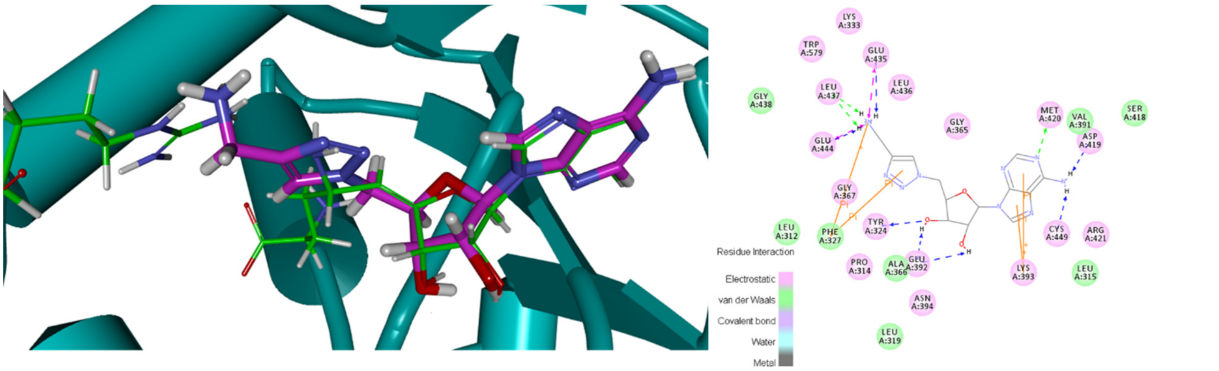

Figure S3: Docked pose of 6 overlayed with sinefungin (green) and a substrate arginine (green). To the right is shown a 2D representation of the interactions formed by the representative compound.

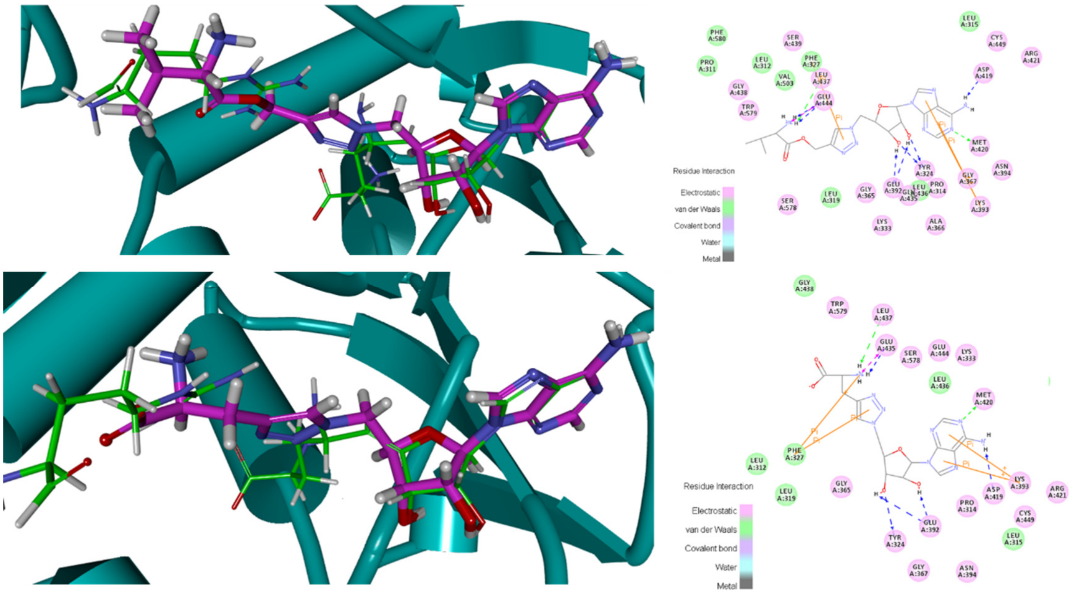

Figure S4. Docked poses of 20 (top) and 19 (bottom) overlayed with sinefungin (green) and a substrate arginine (green). To the right is shown a 2D representation of the interactions formed by the representative compounds.
