# Supplementary material for: Synthesis and Activity of Triazole-Adenosine Analogs as Protein Arginine Methyltransferase 5 Inhibitors
Source: Molecules. 2022 Jun 11;27(12):3779. doi: 10.3390/molecules27123779 (PMC9228412; doi:10.3390/molecules27123779)
Supplement: Supplementary file 1 [file molecules-27-03779-s001.zip › Supplementary Spectral Data.pptx]

## Slide 1
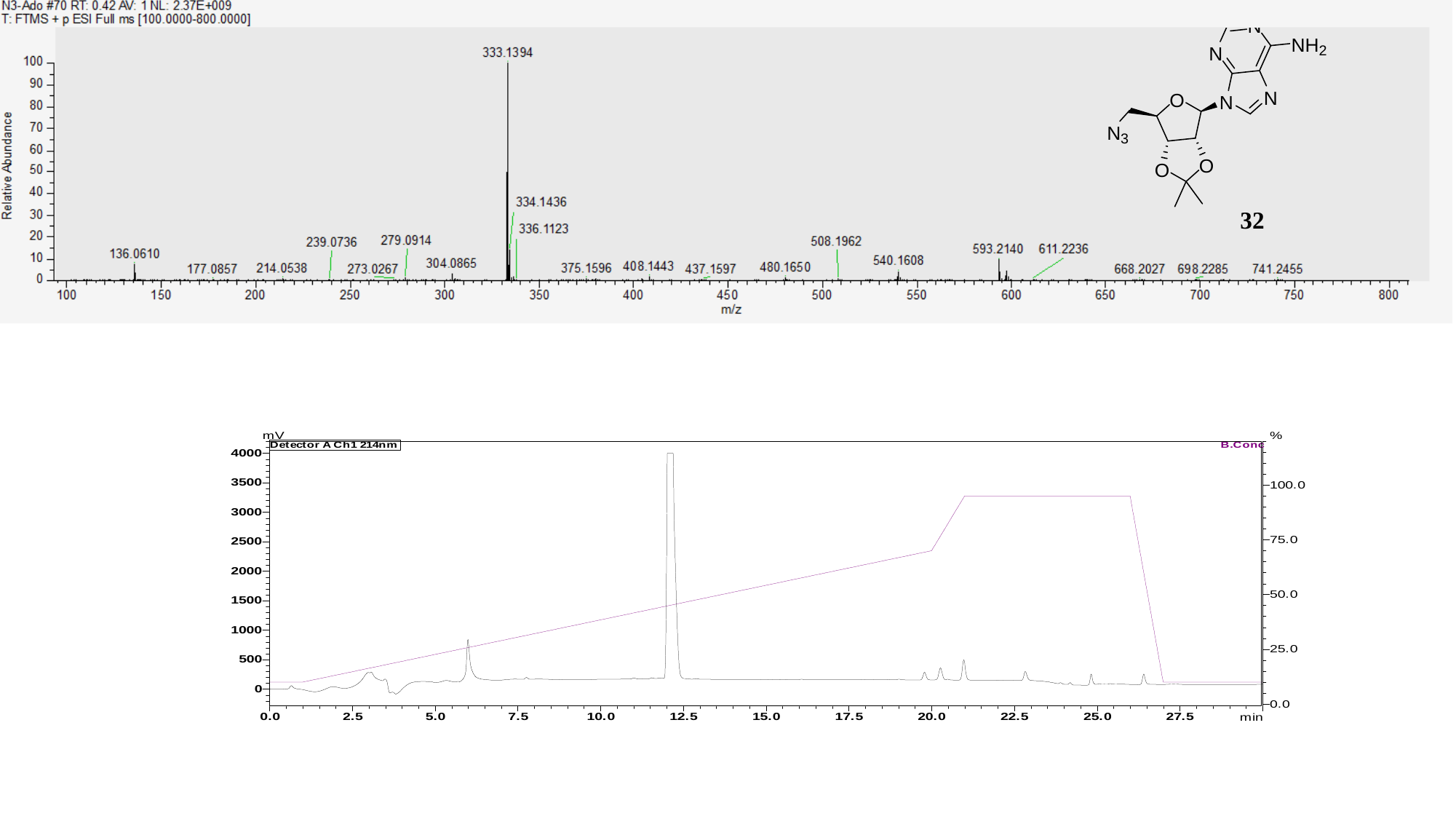

32

## Slide 2
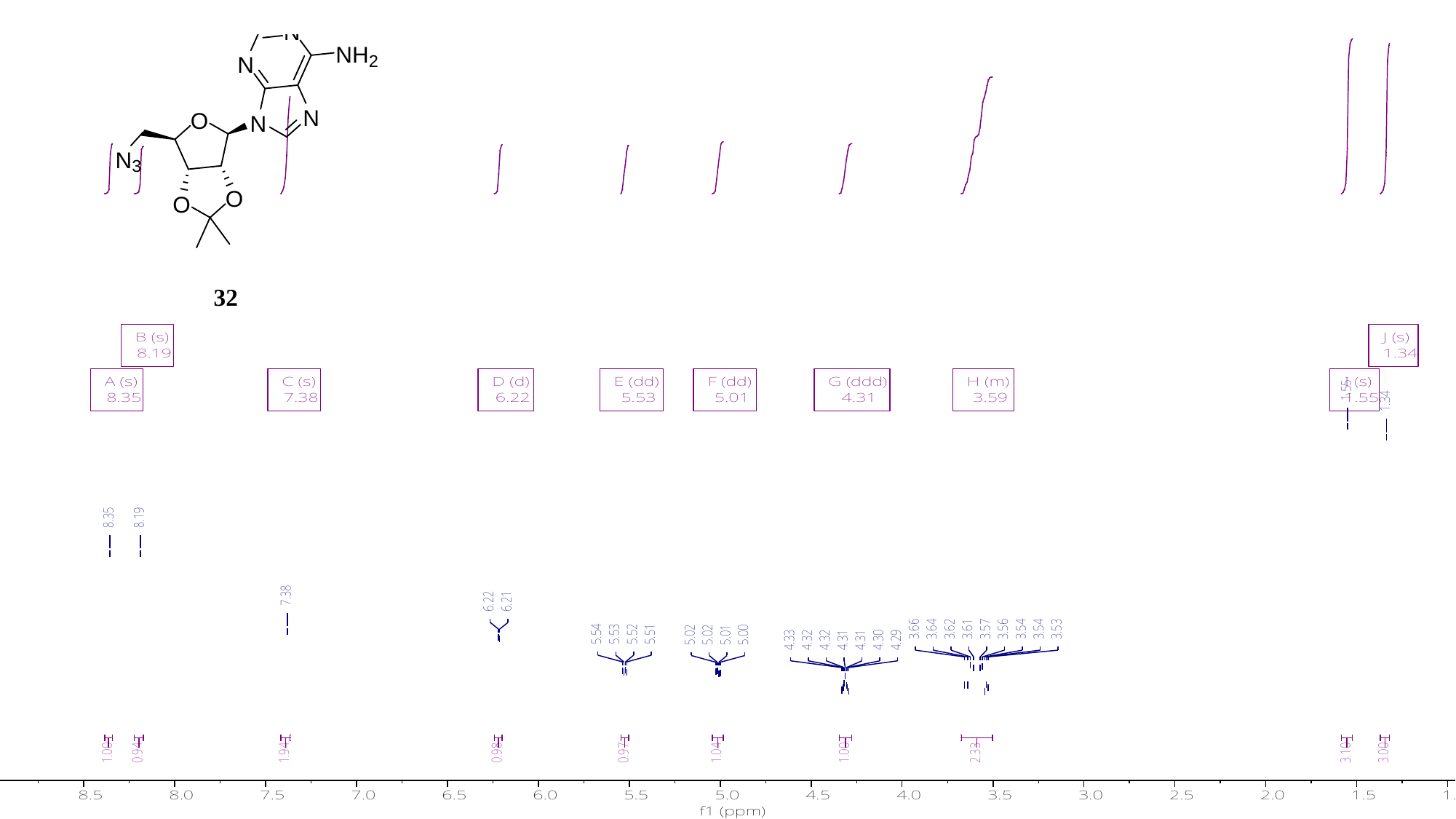

32

## Slide 3
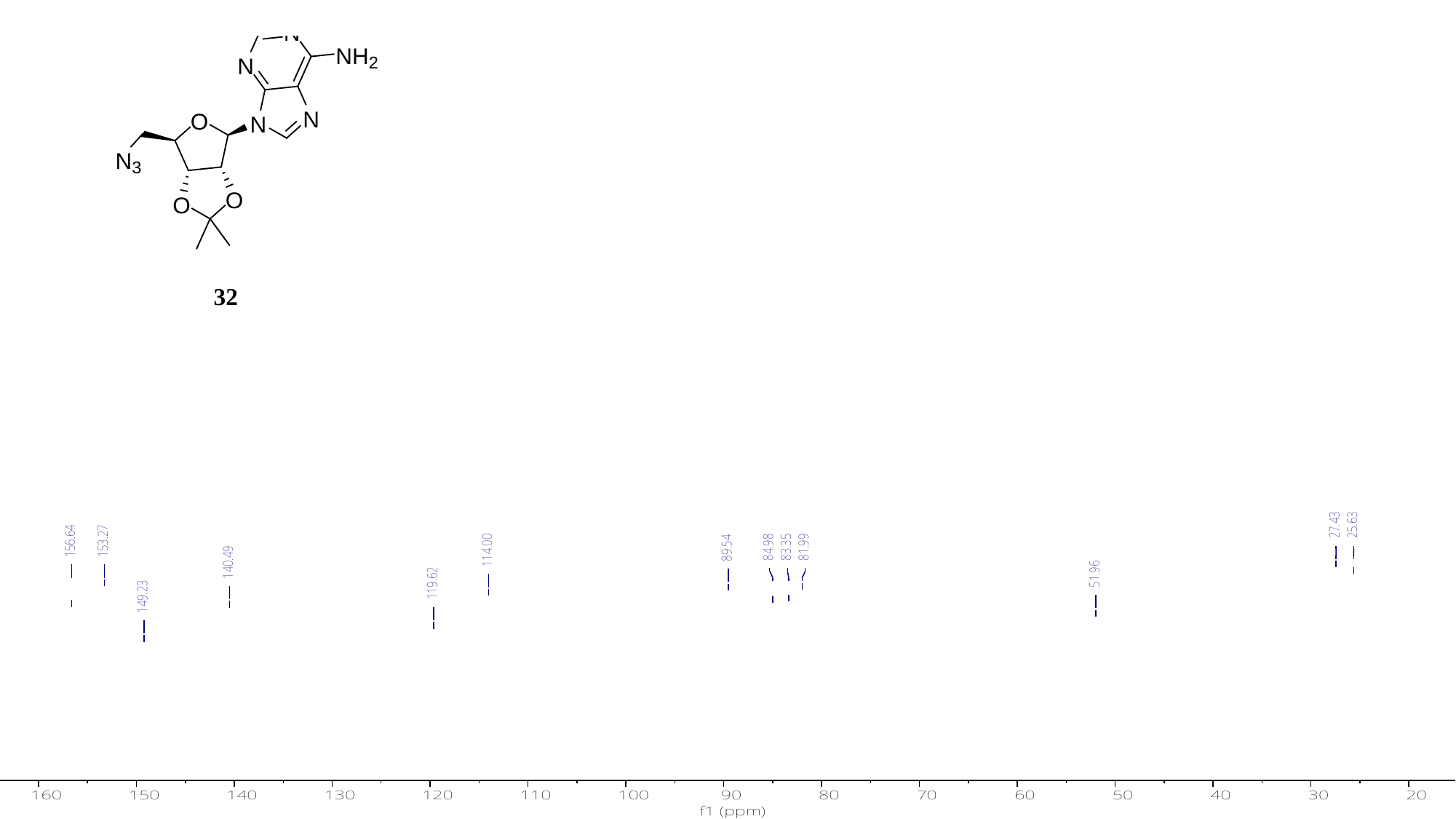

32

## Slide 4
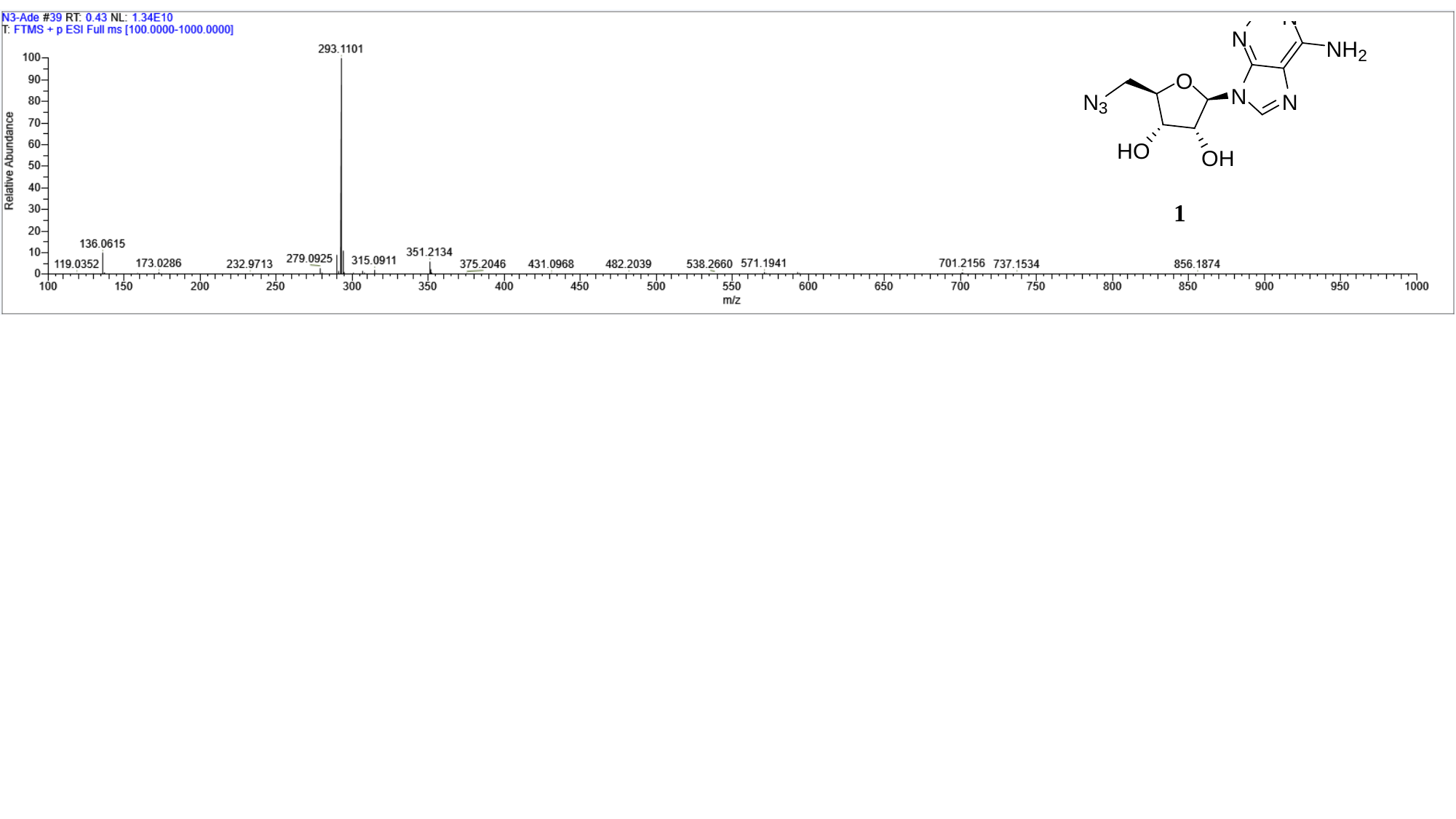

1

## Slide 5
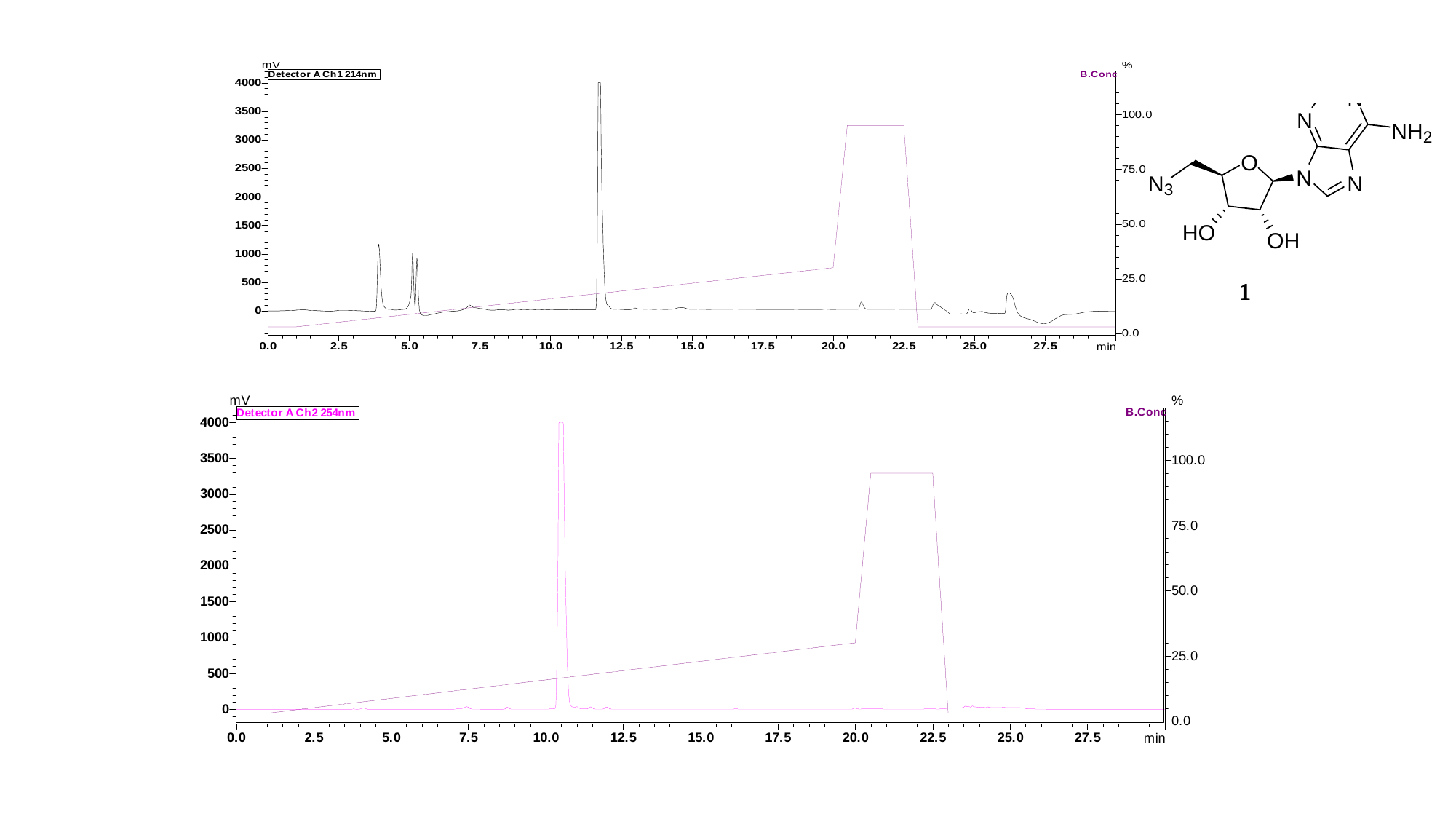

1

## Slide 6
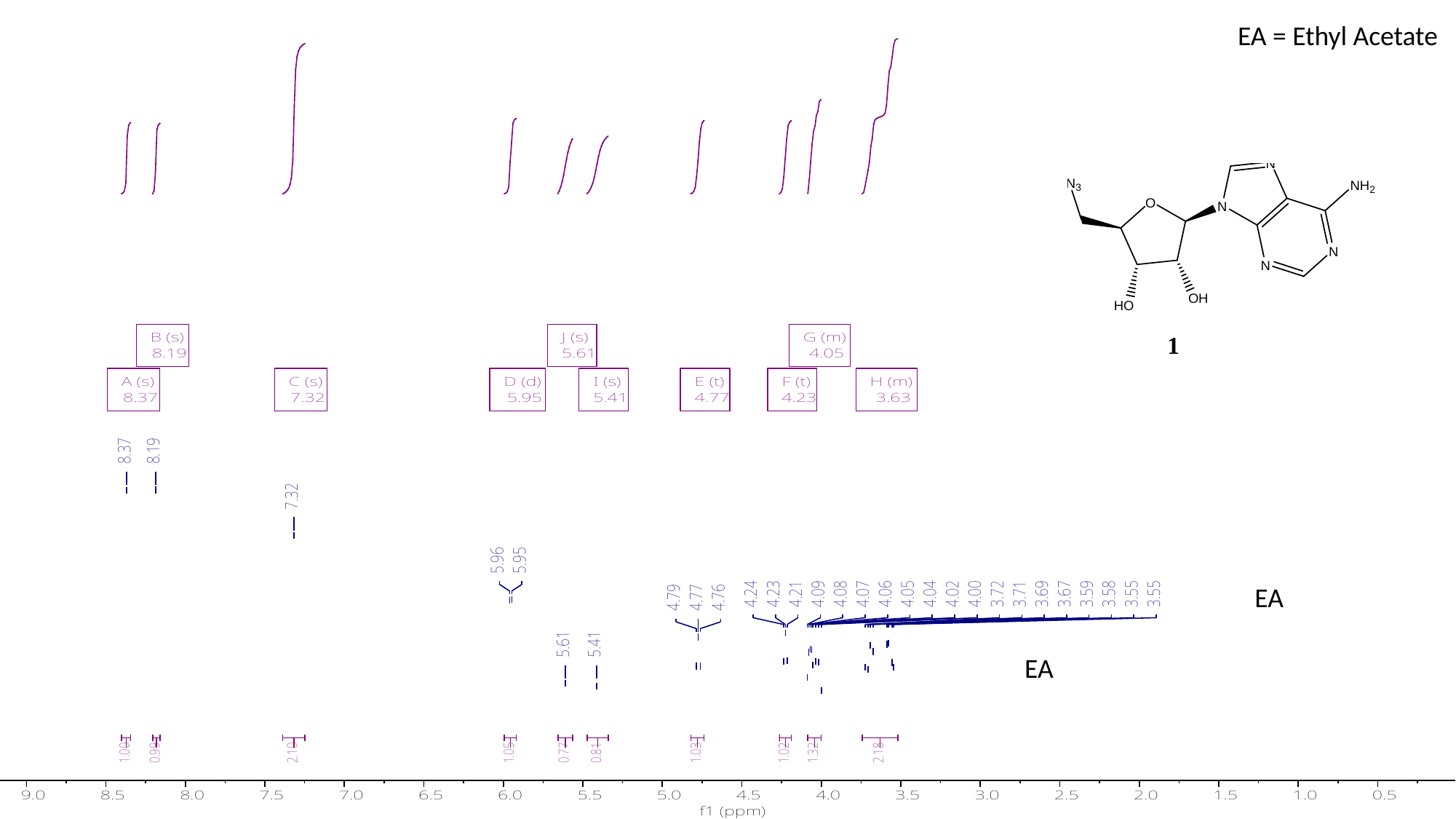

EA = Ethyl Acetate
1
EA
EA

## Slide 7
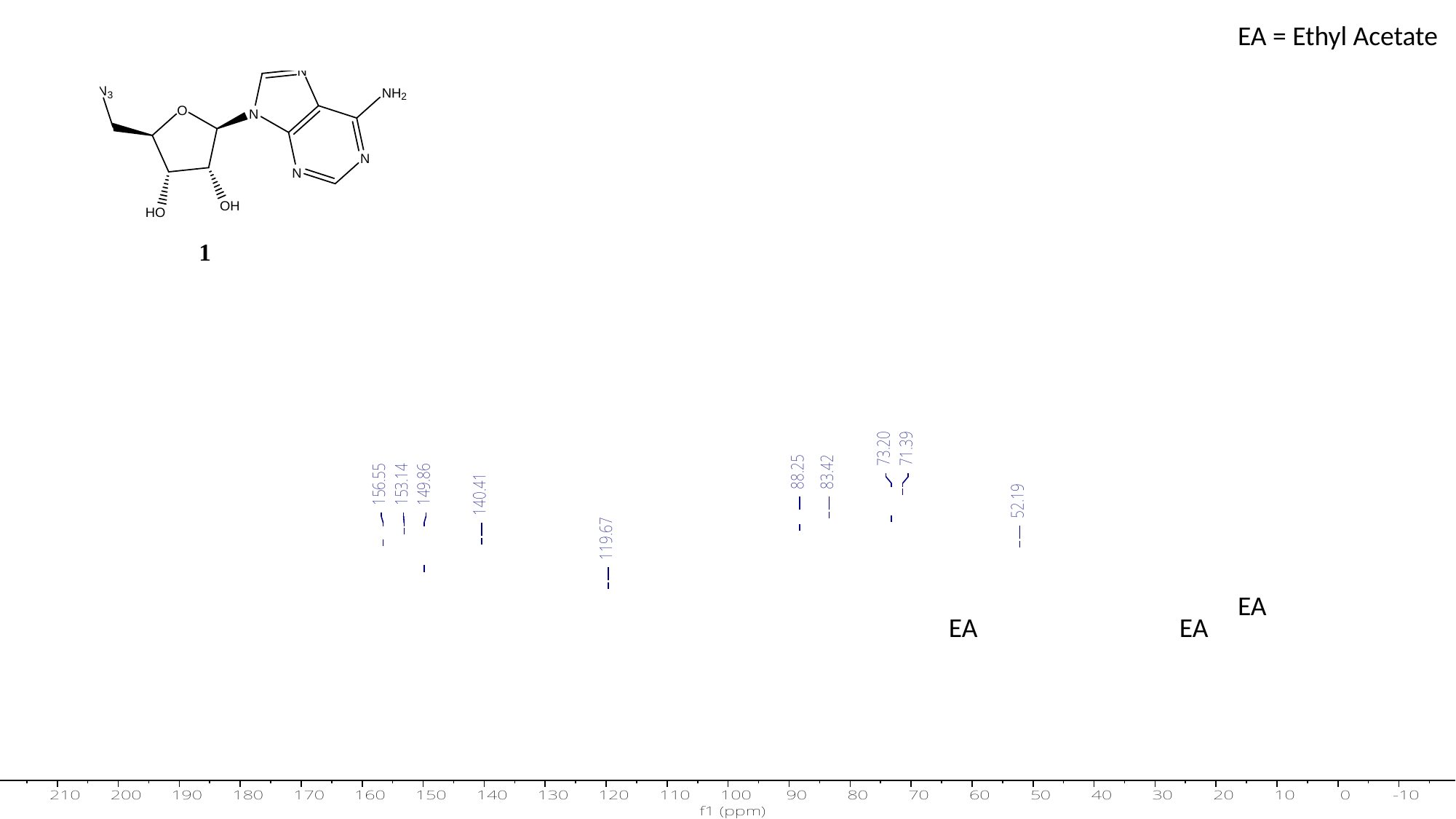

EA = Ethyl Acetate
1
EA
EA
EA

## Slide 8
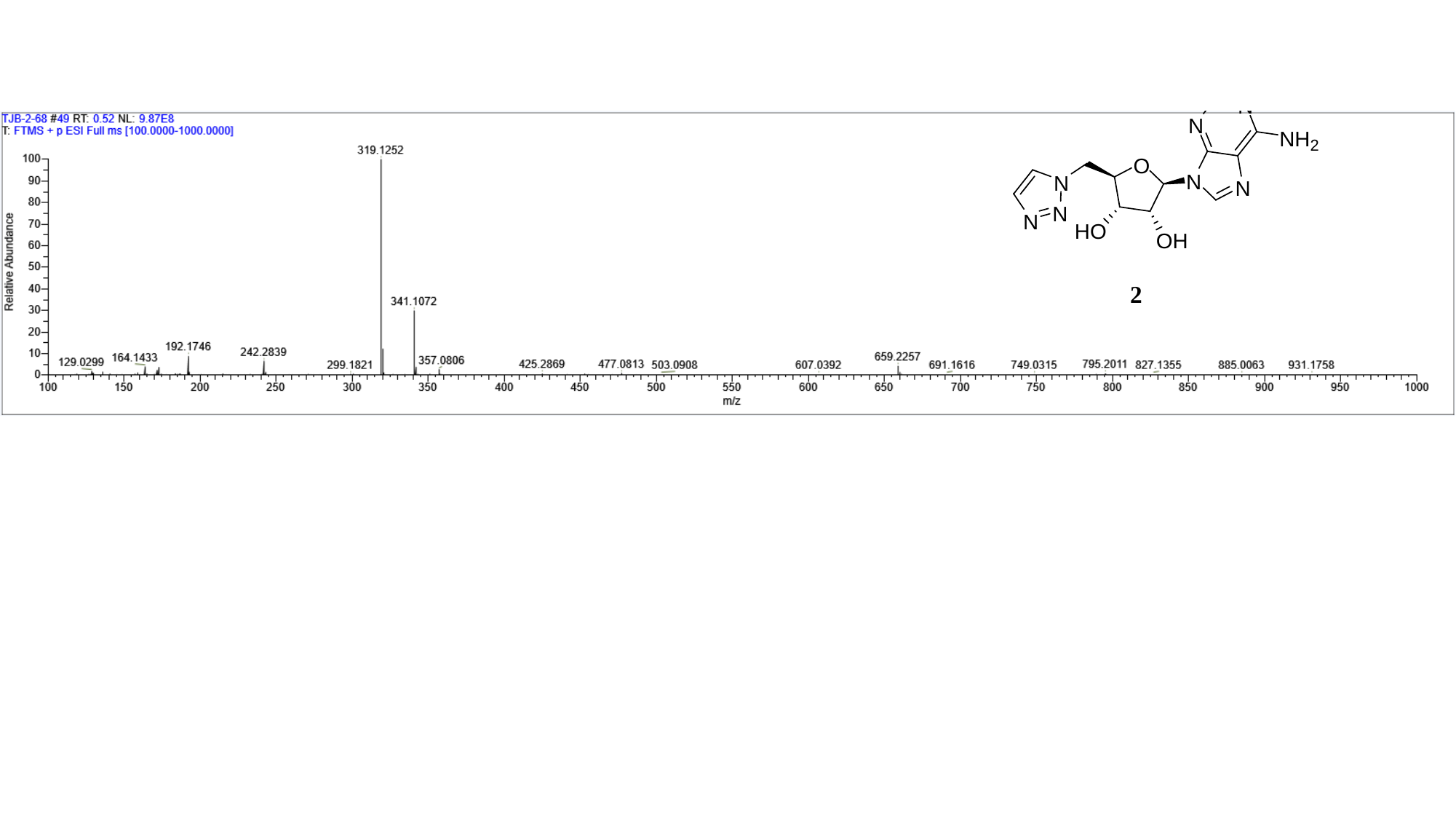

2

## Slide 9
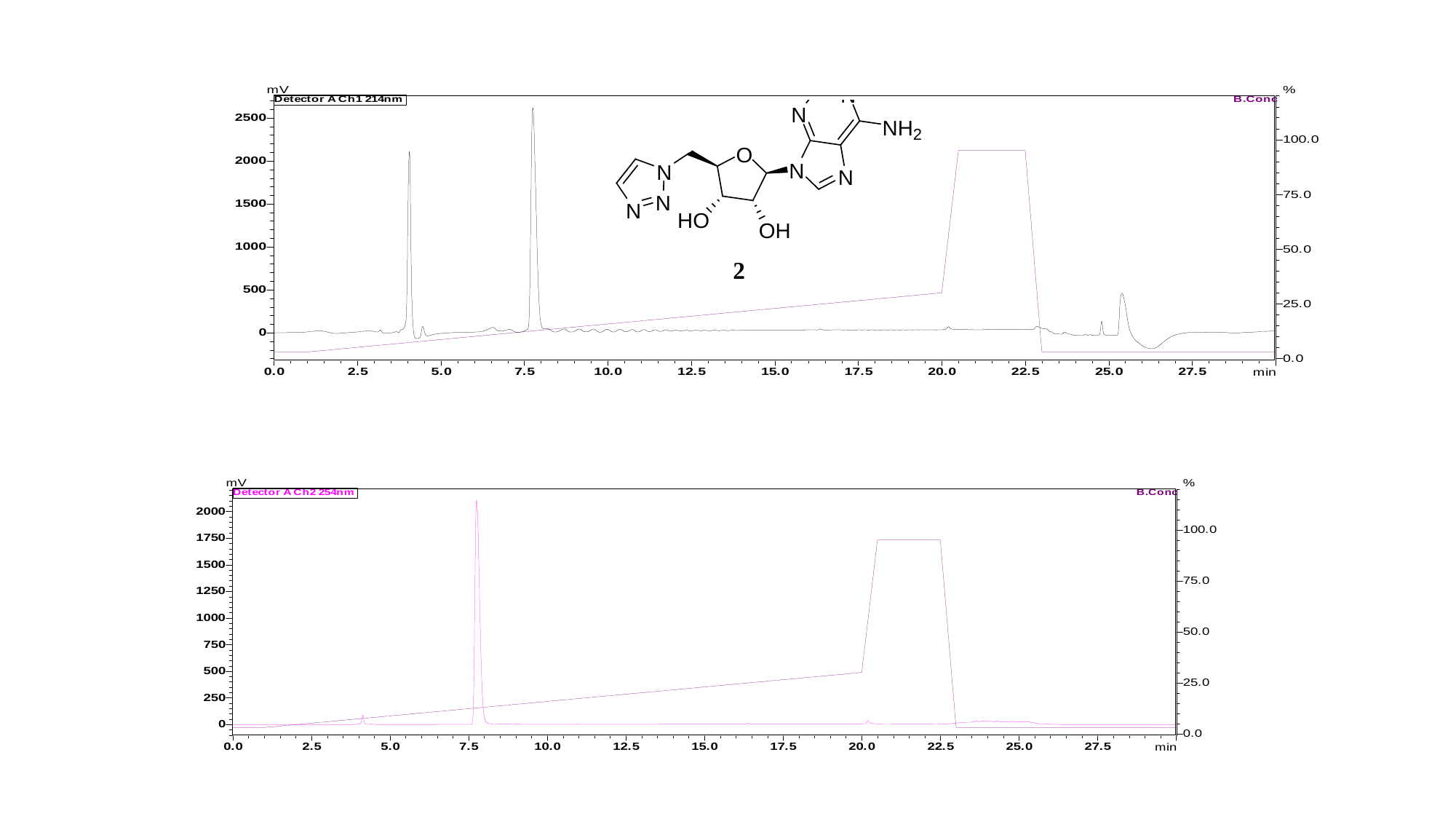

2

## Slide 10
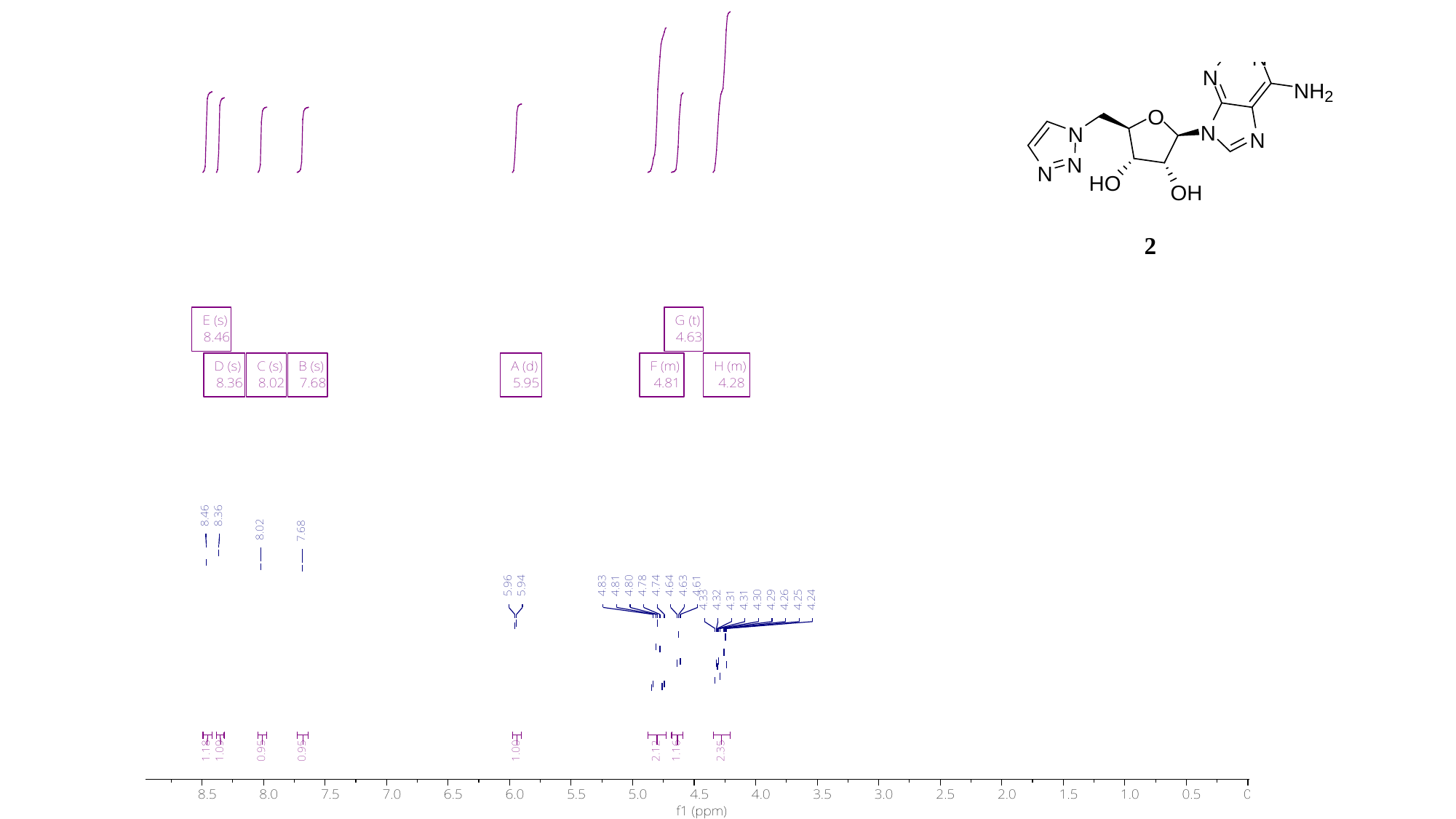

2

## Slide 11
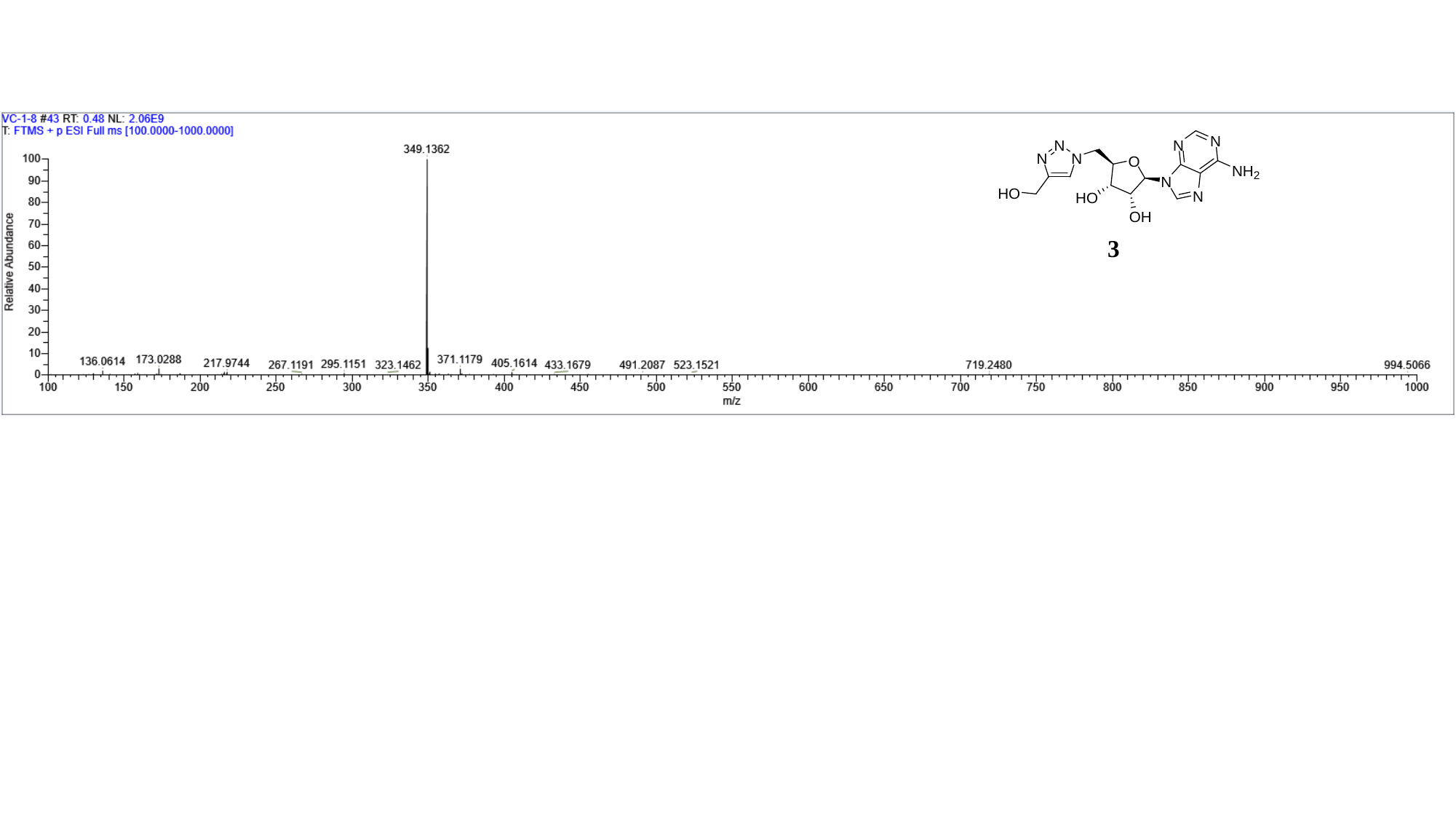

3

## Slide 12
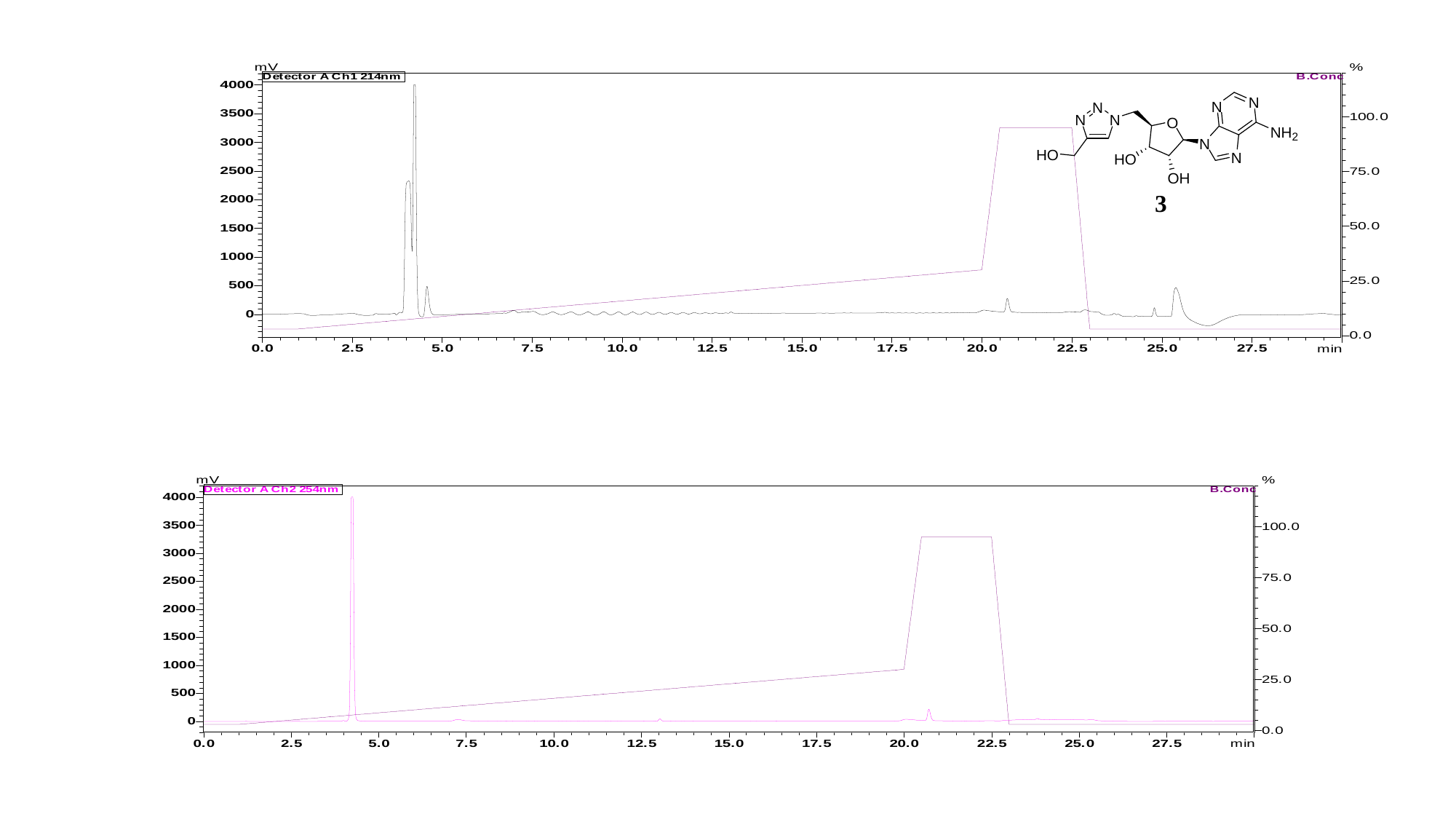

3

## Slide 13
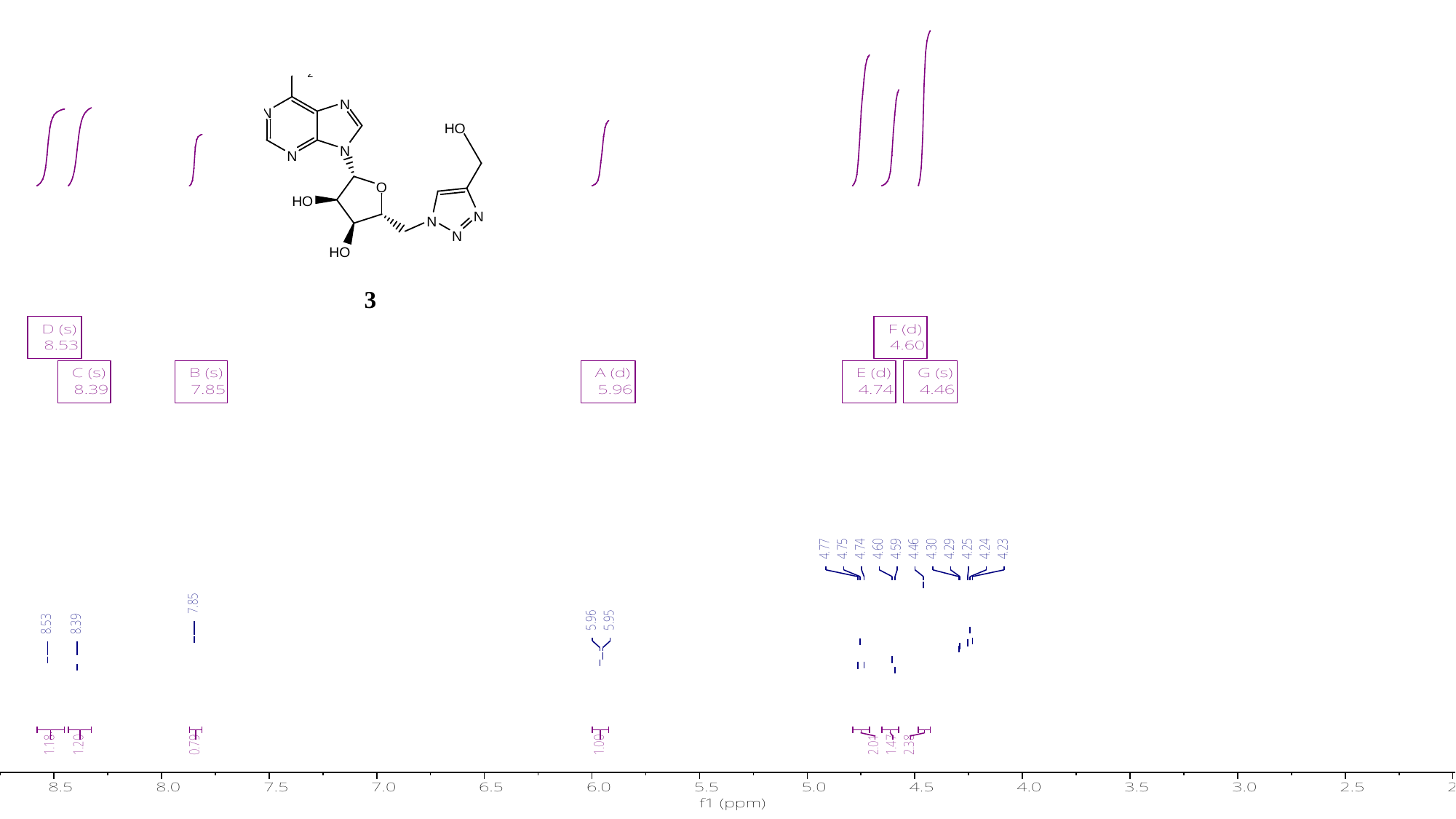

3

## Slide 14
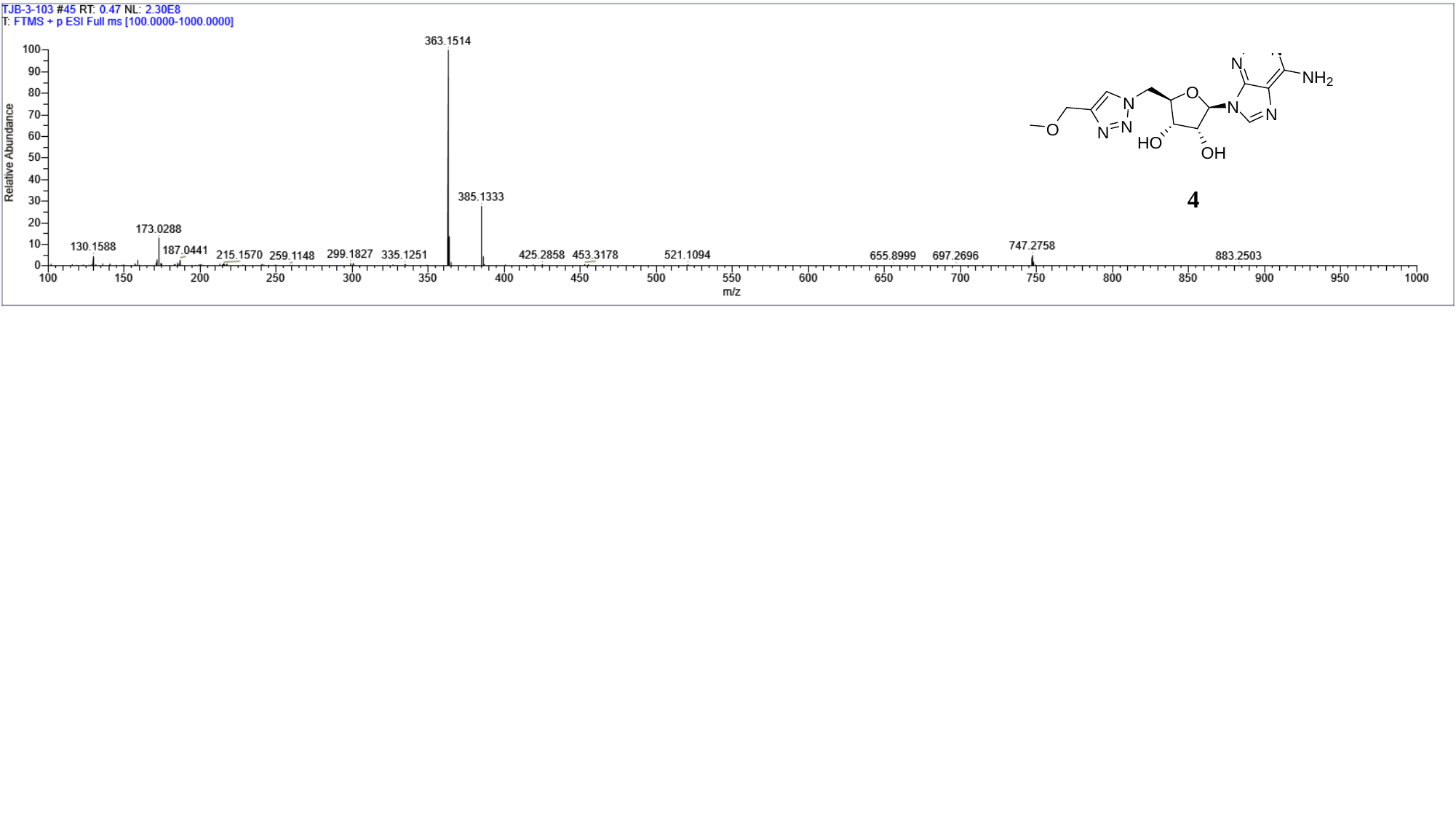

4

## Slide 15
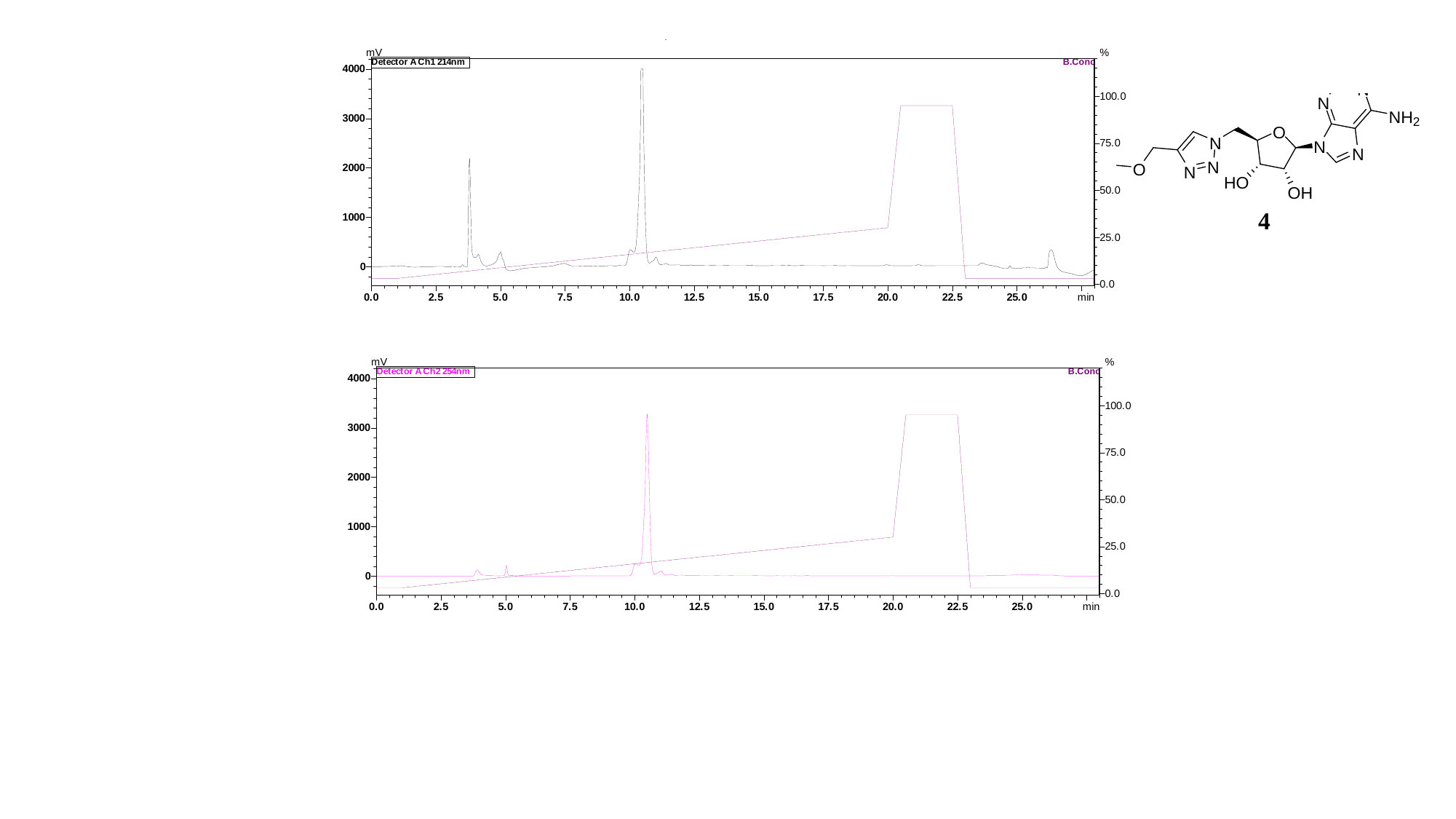

4

## Slide 16
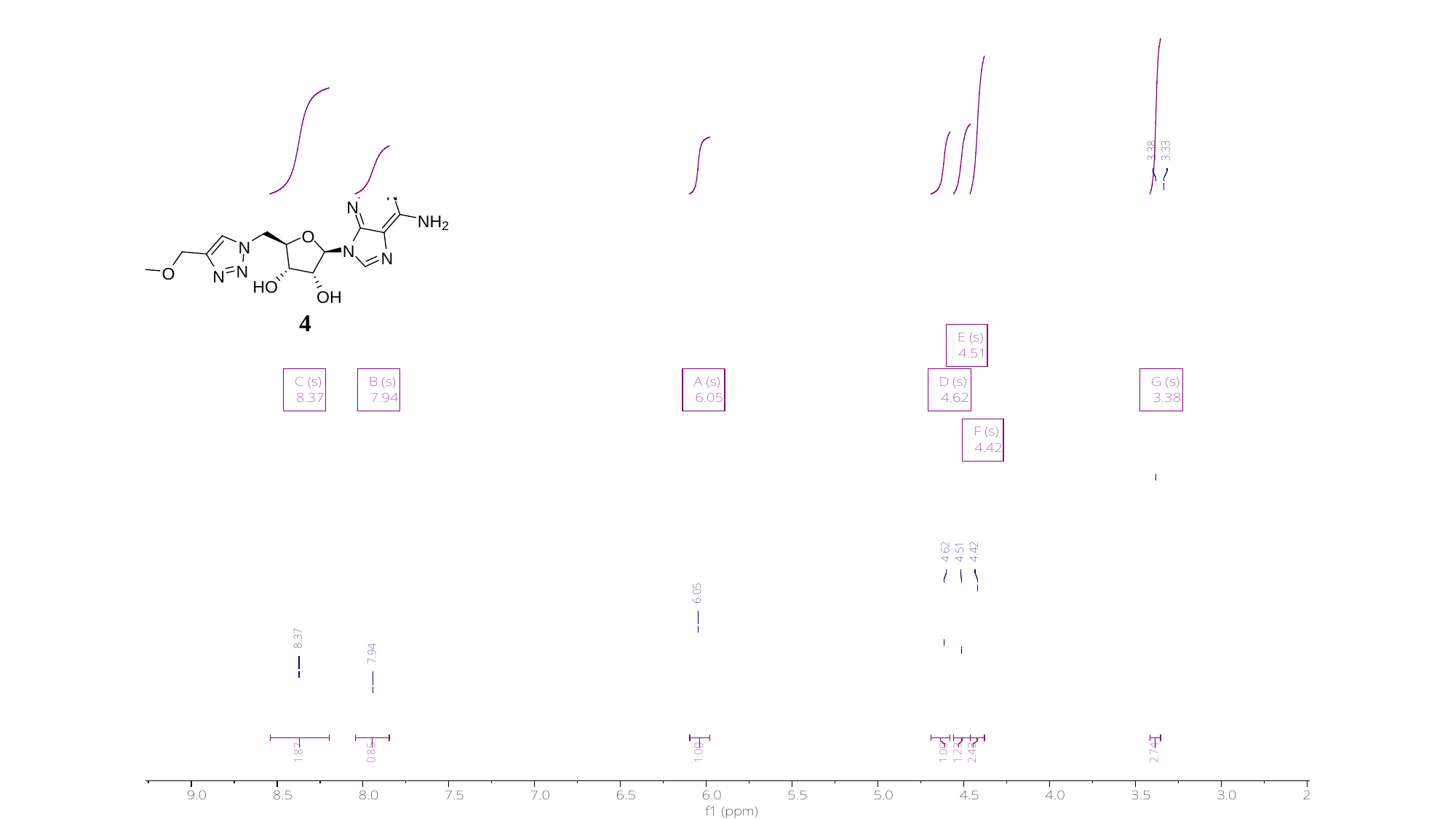

4

## Slide 17
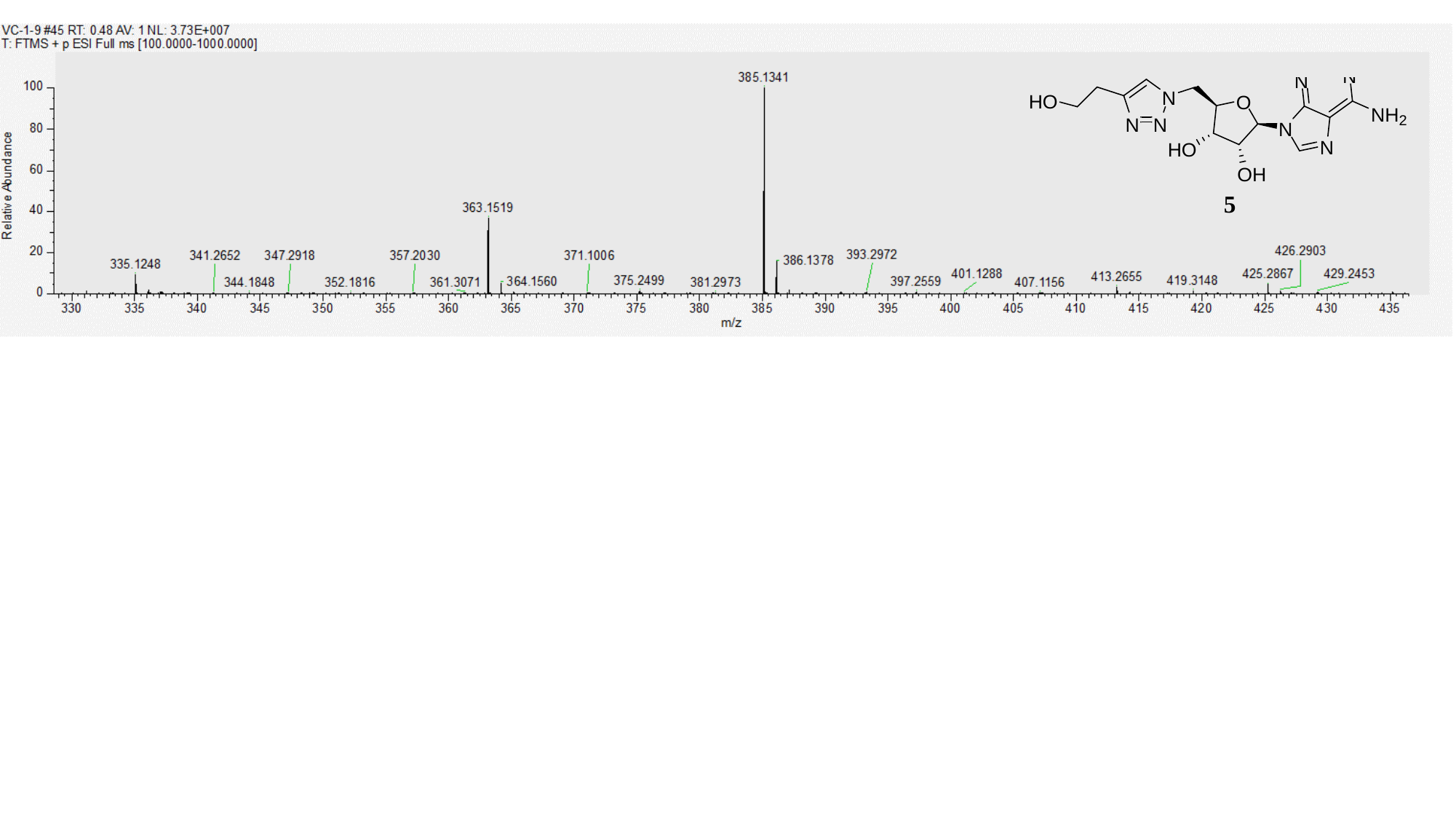

5

## Slide 18
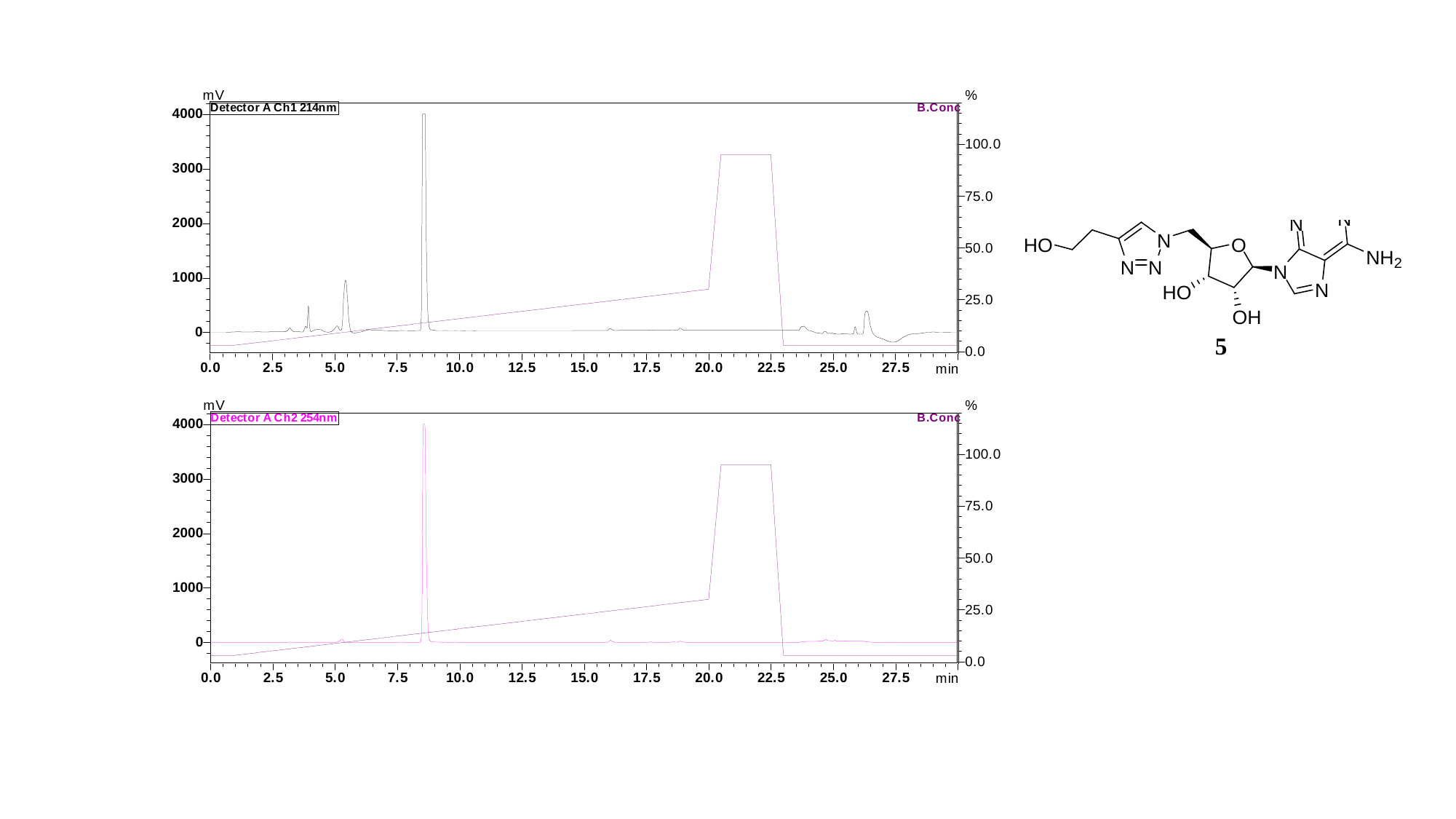

5

## Slide 19
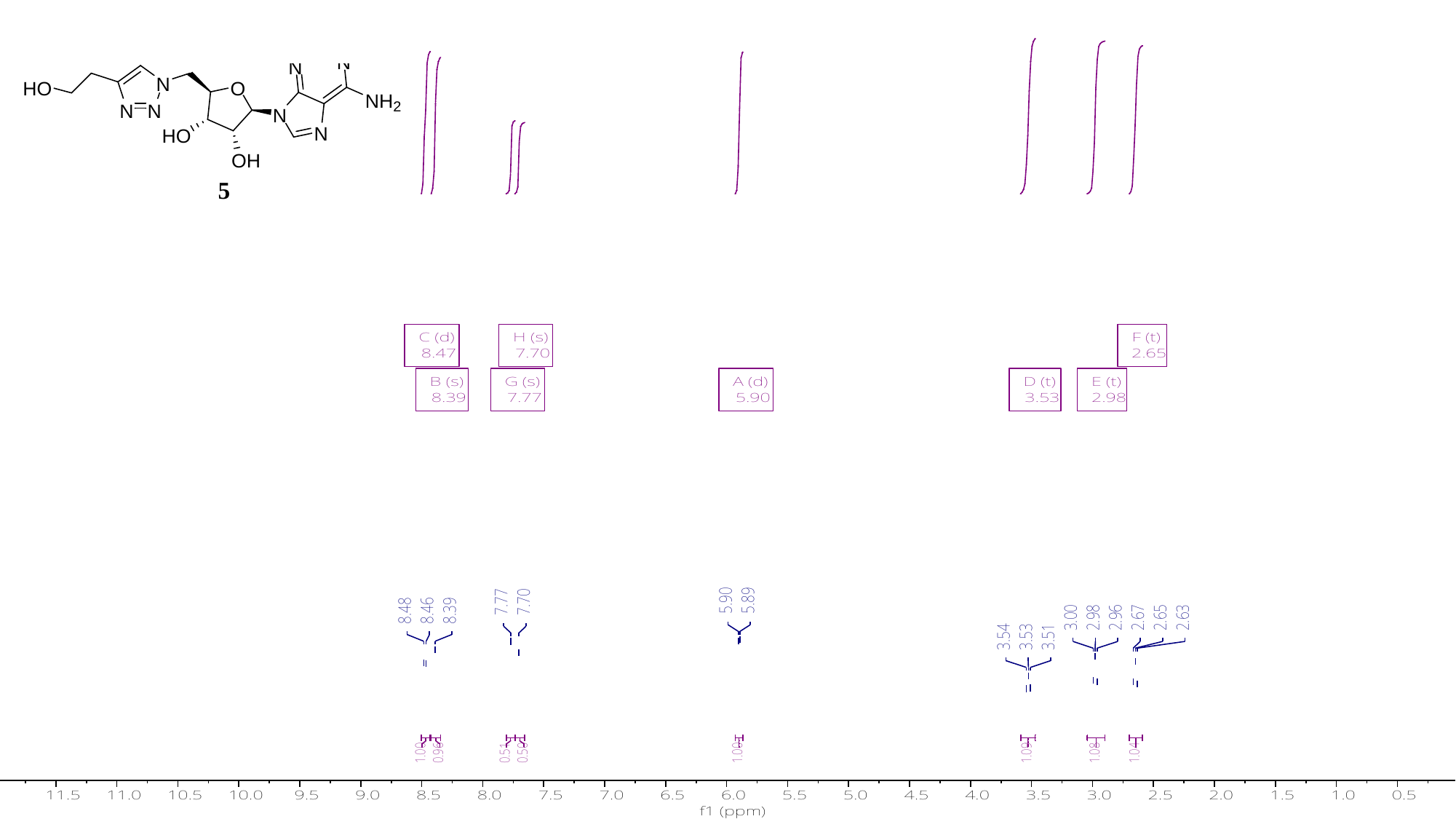

5

## Slide 20
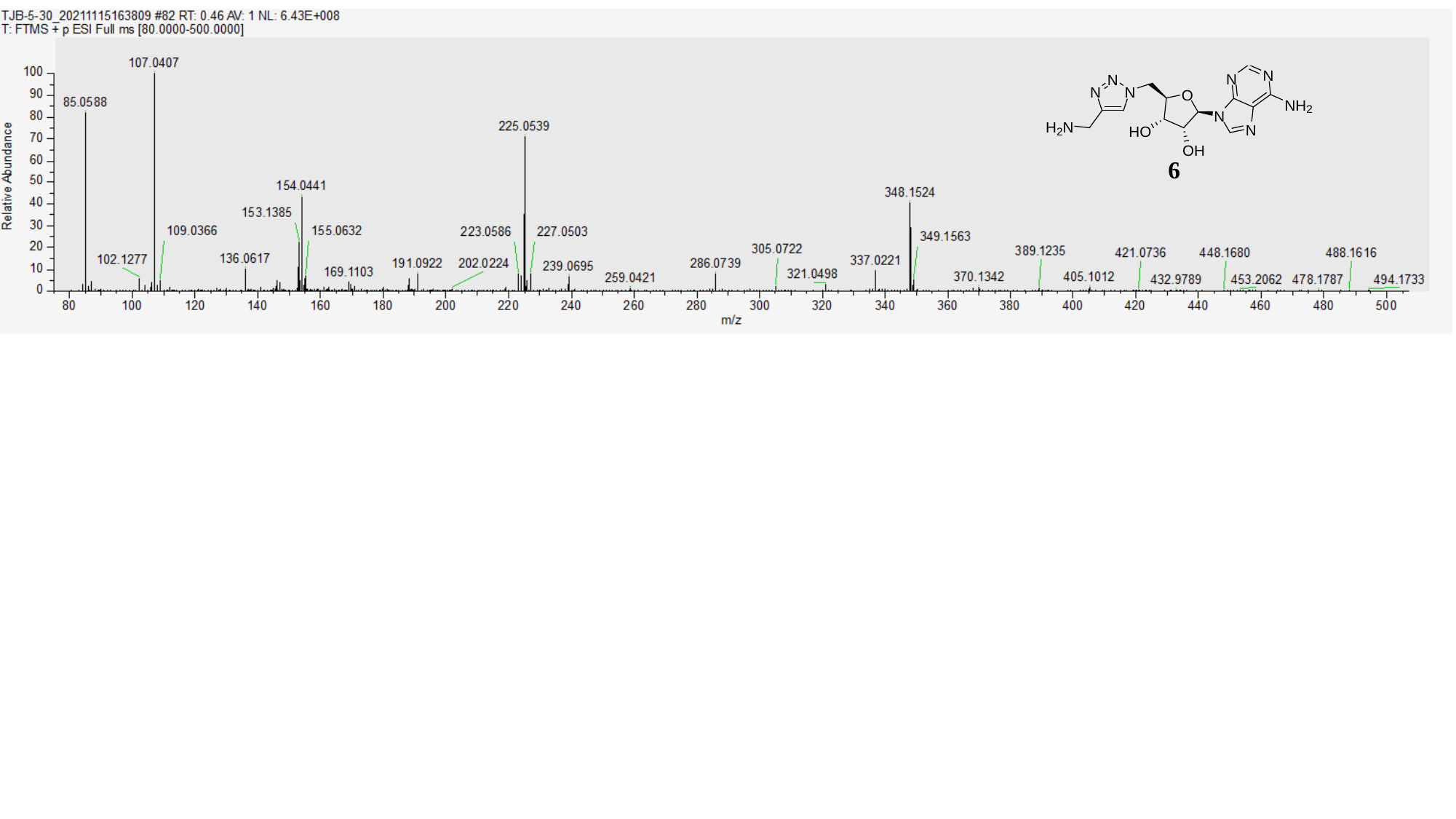

6

## Slide 21
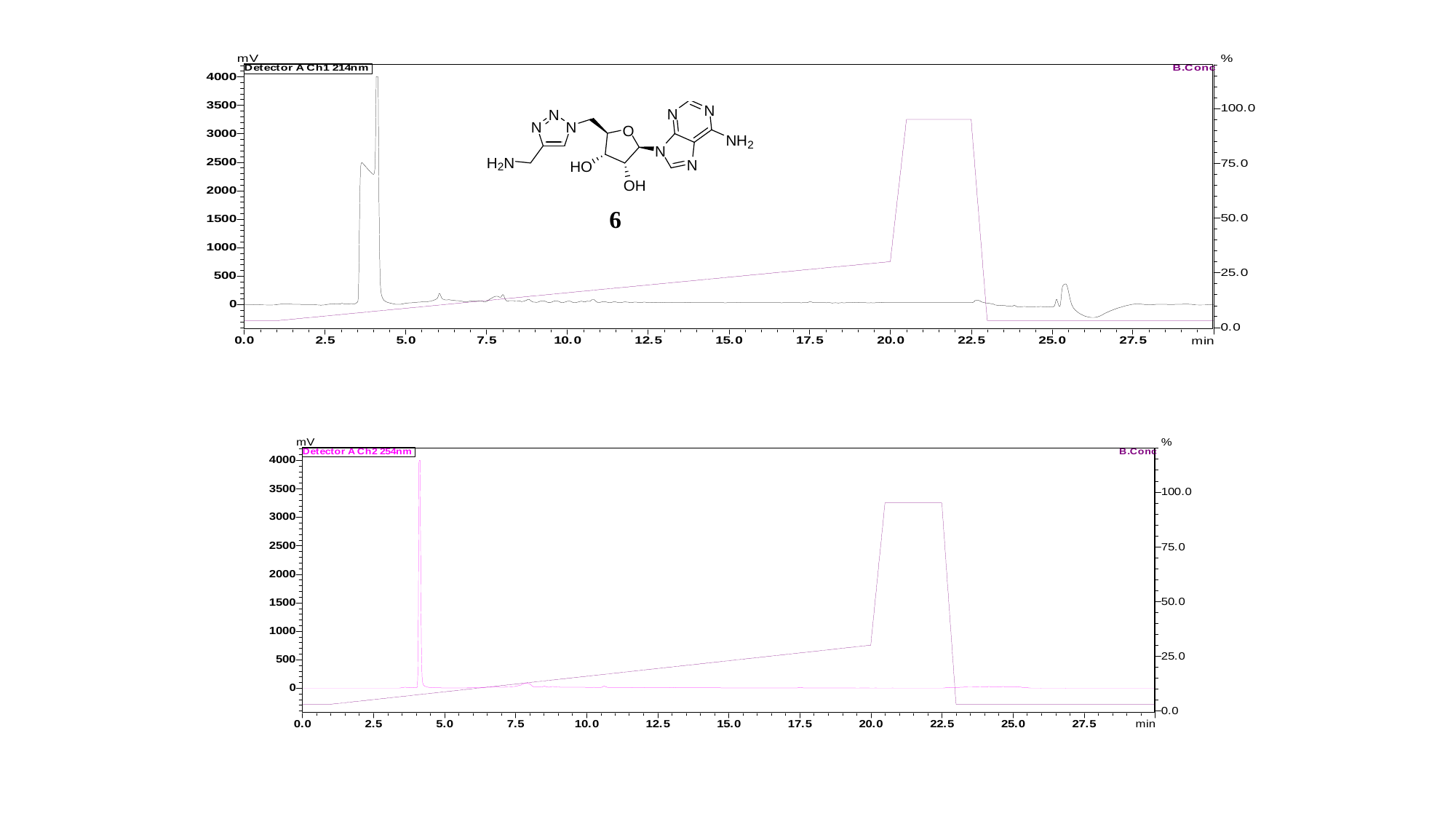

6

## Slide 22
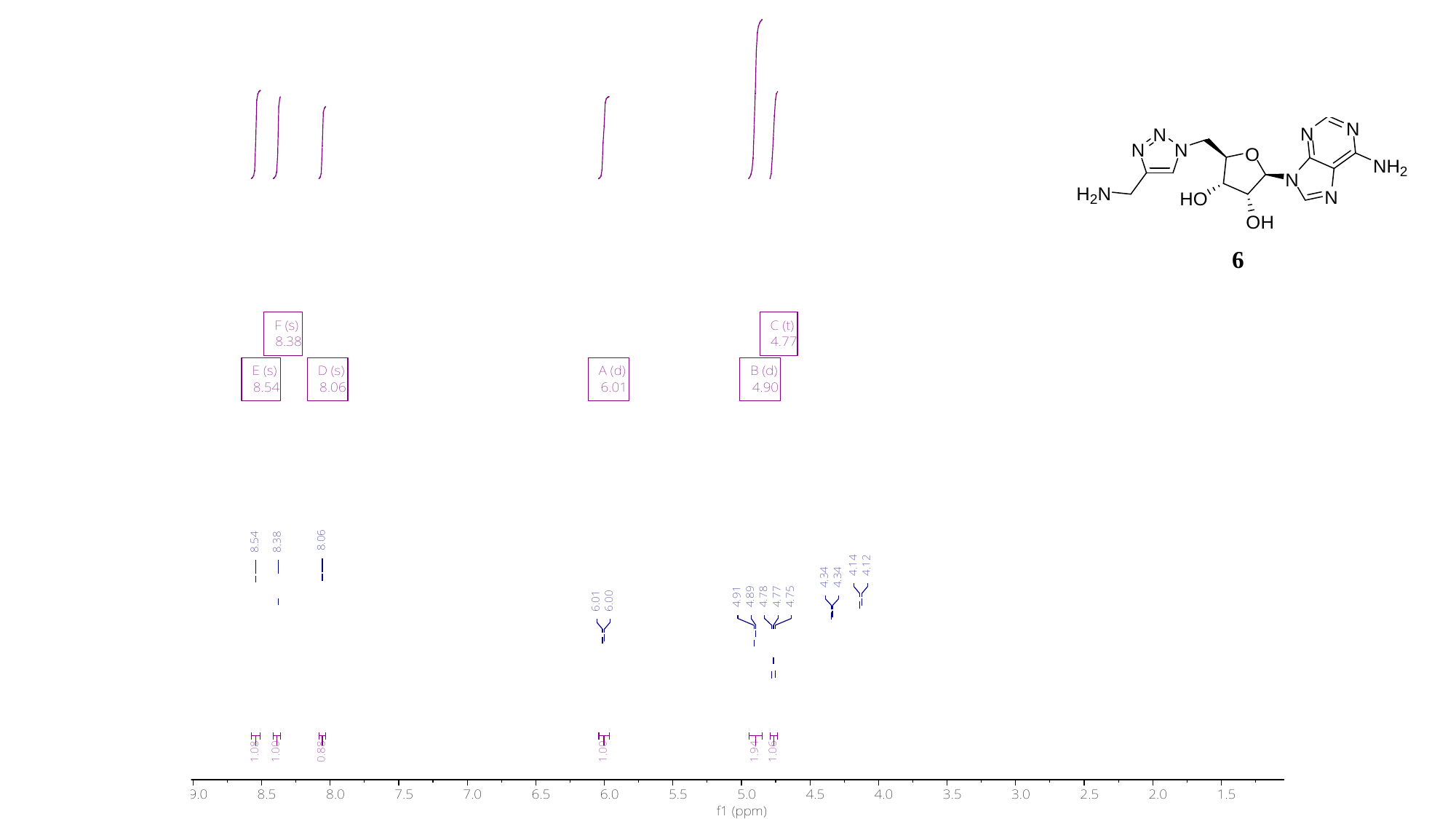

6

## Slide 23
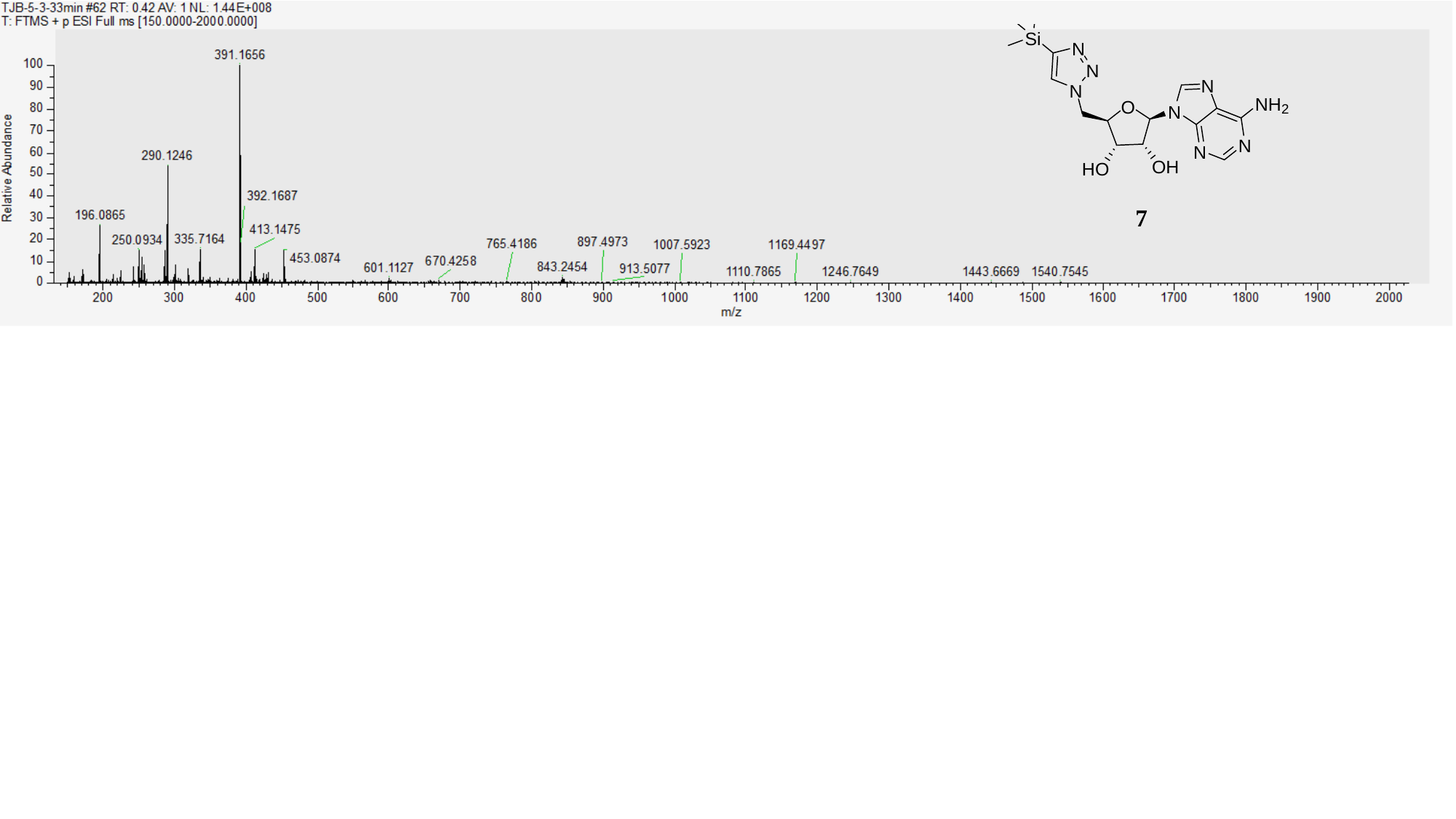

7

## Slide 24
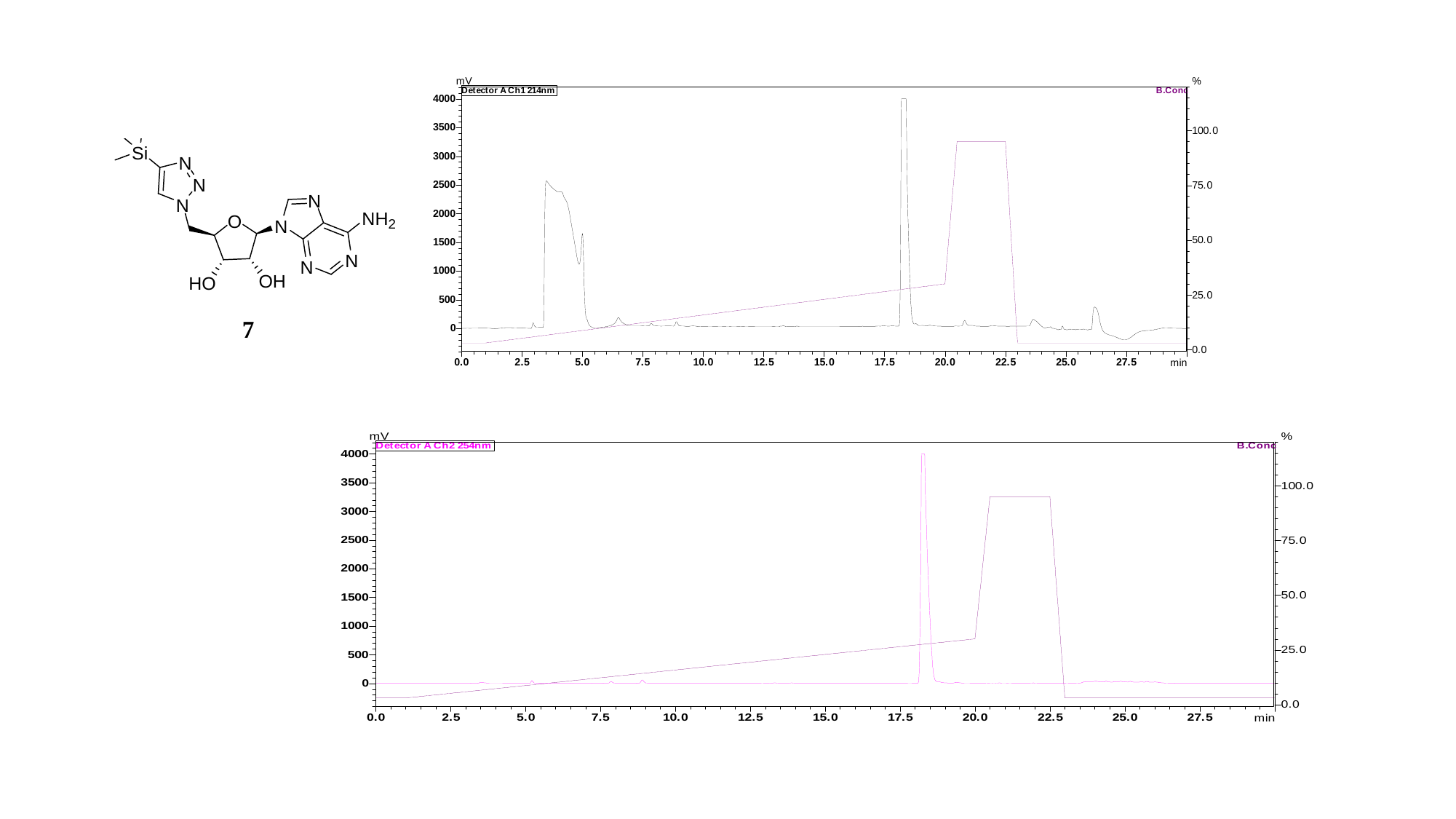

7

## Slide 25
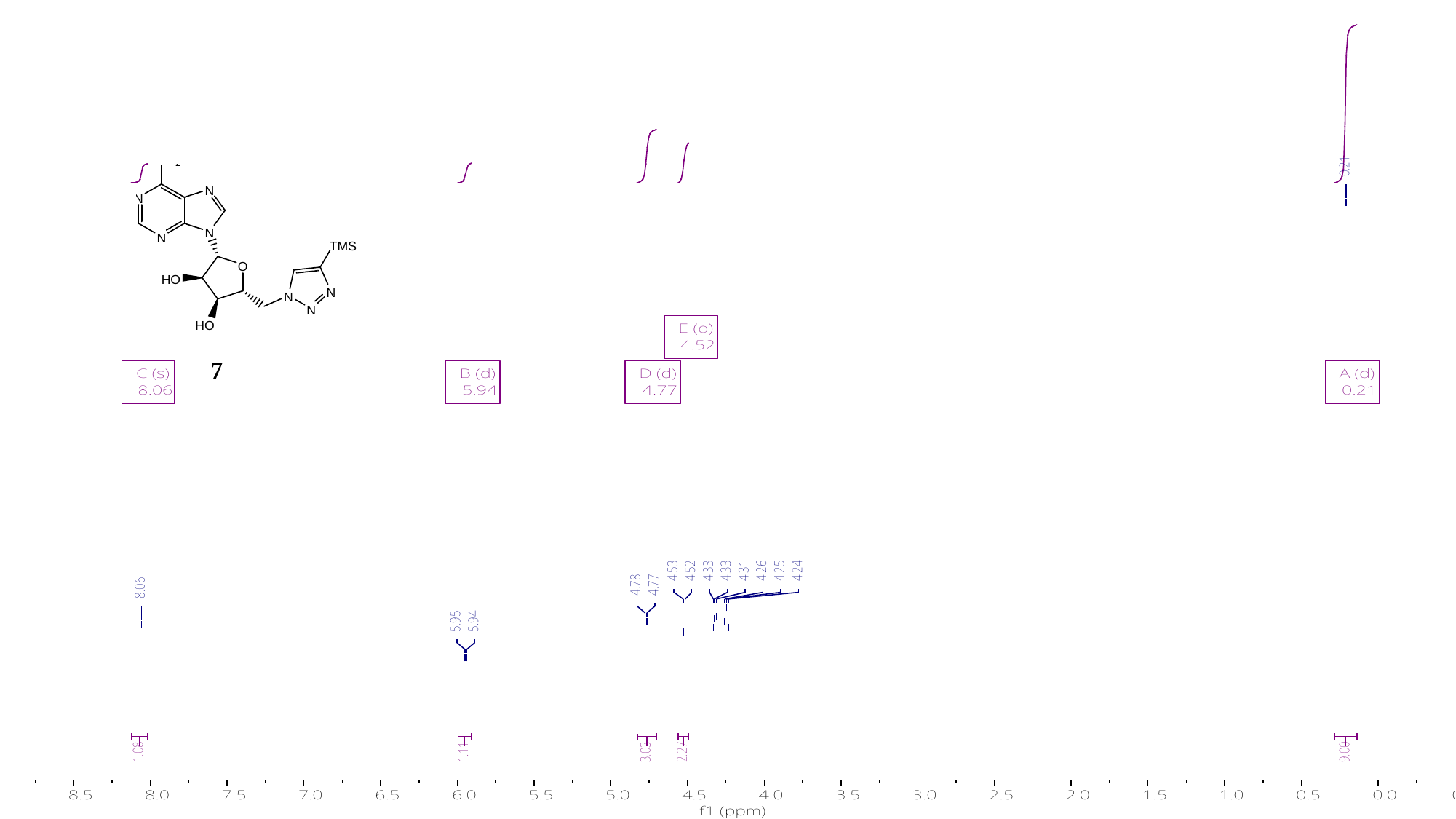

7

## Slide 26
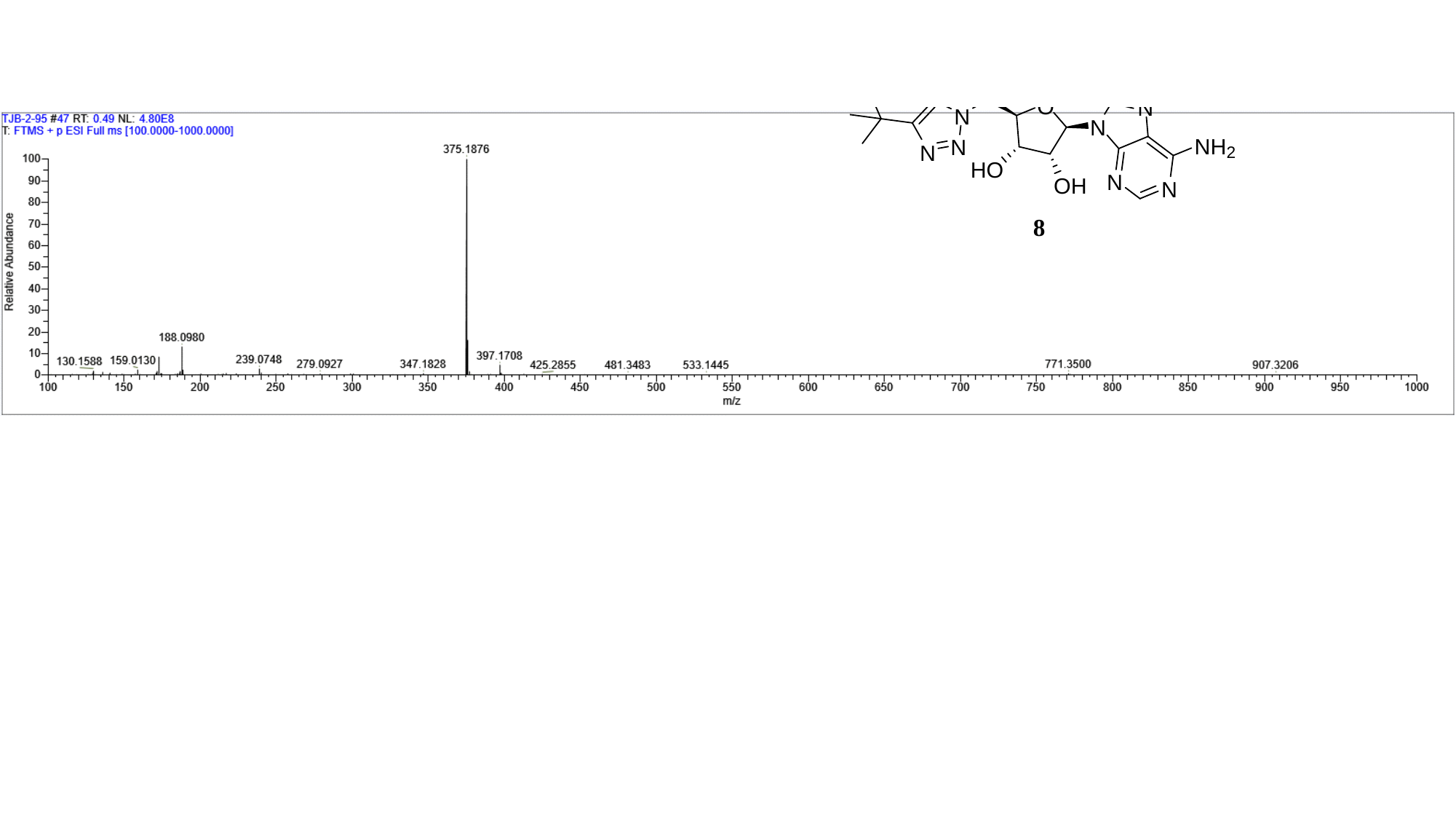

8

## Slide 27
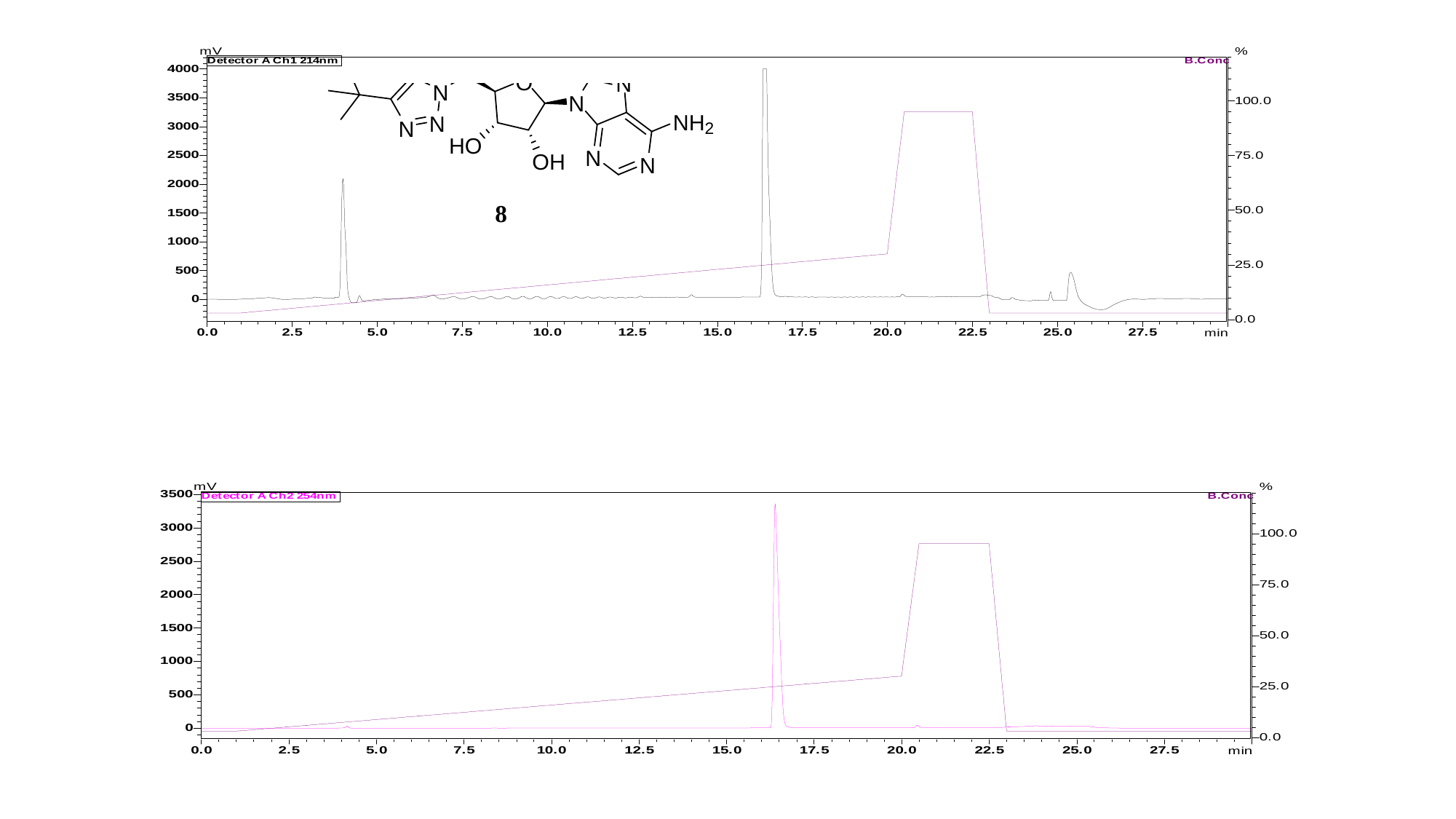

8

## Slide 28
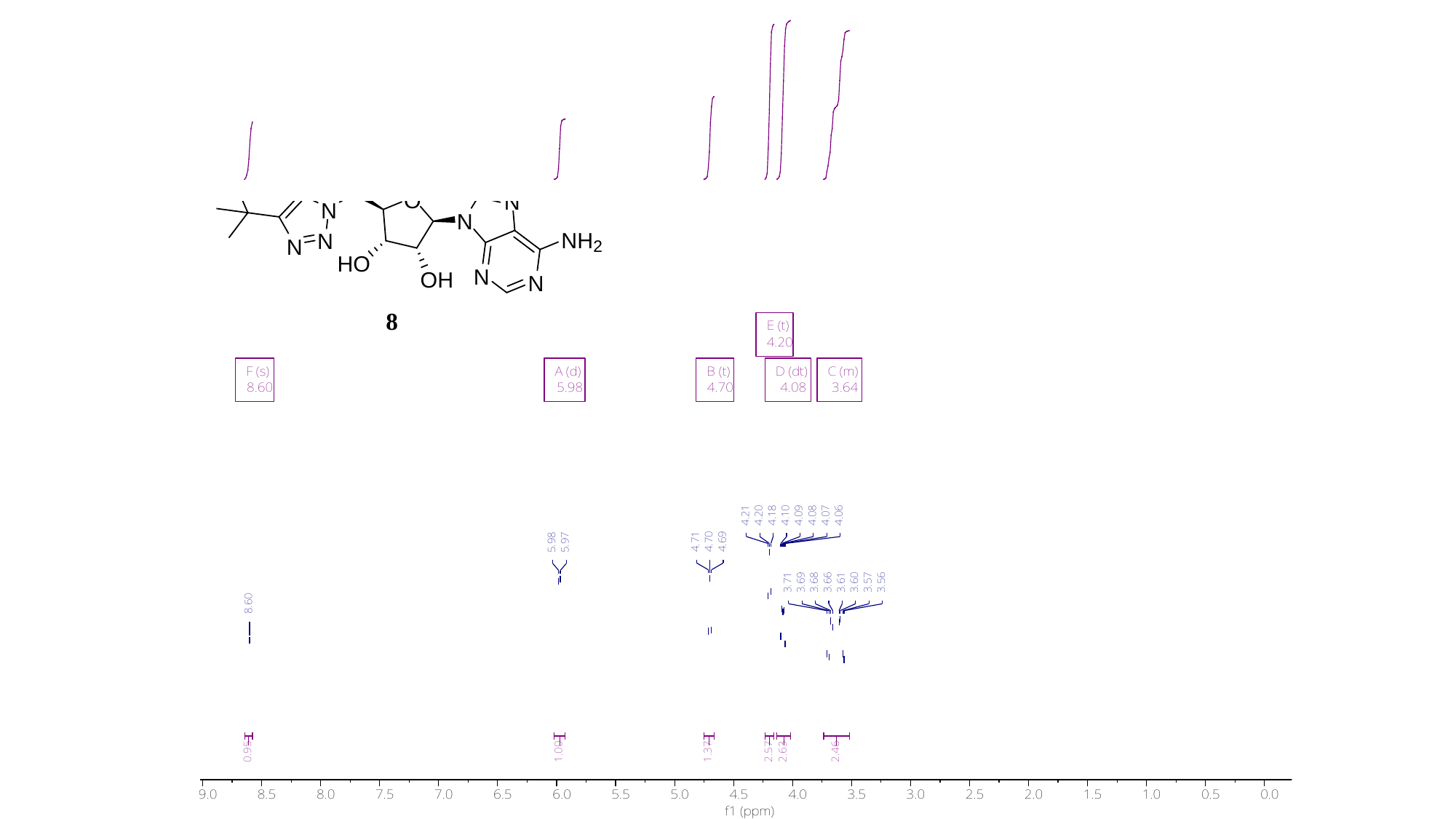

8

## Slide 29
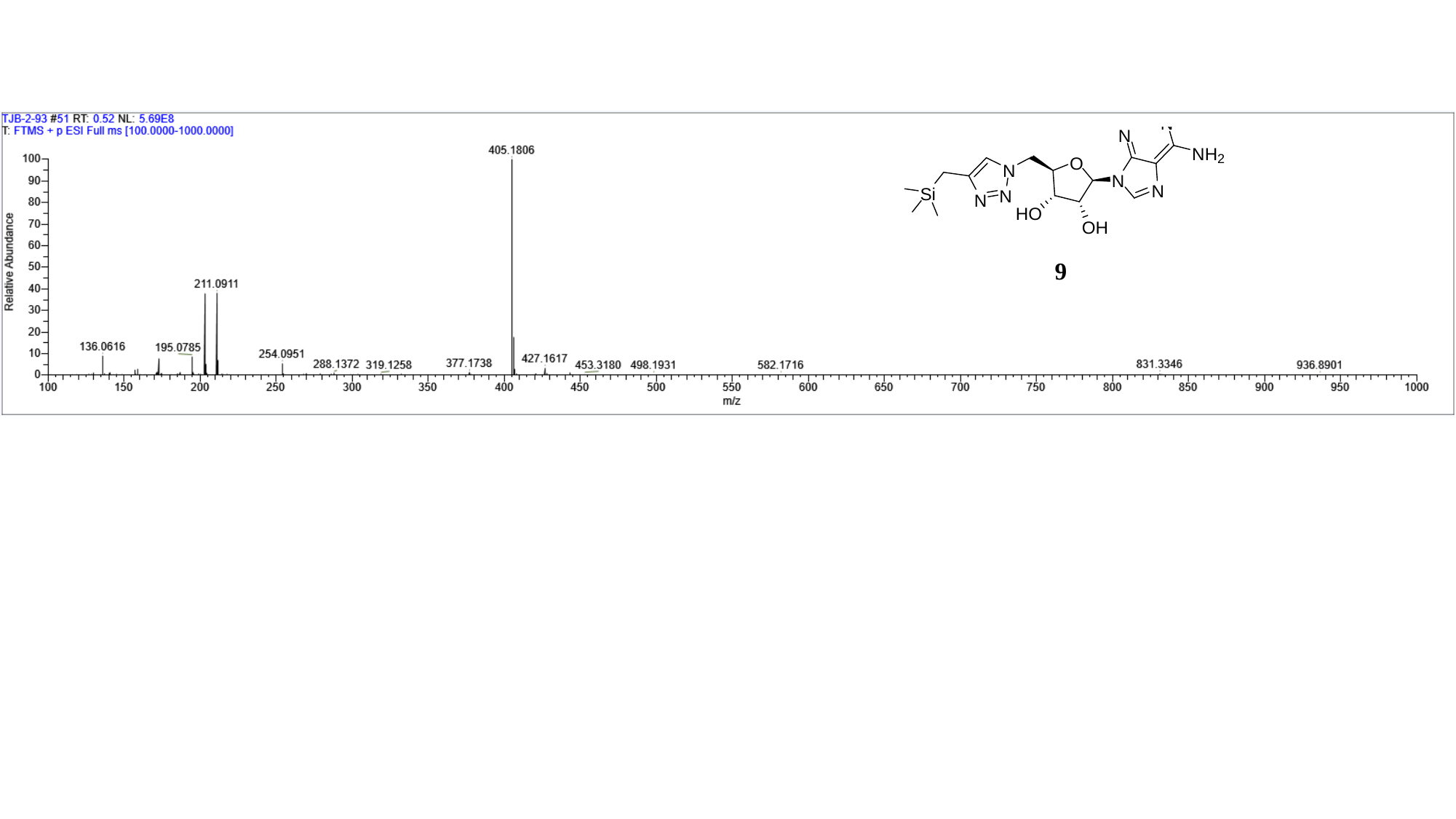

9

## Slide 30
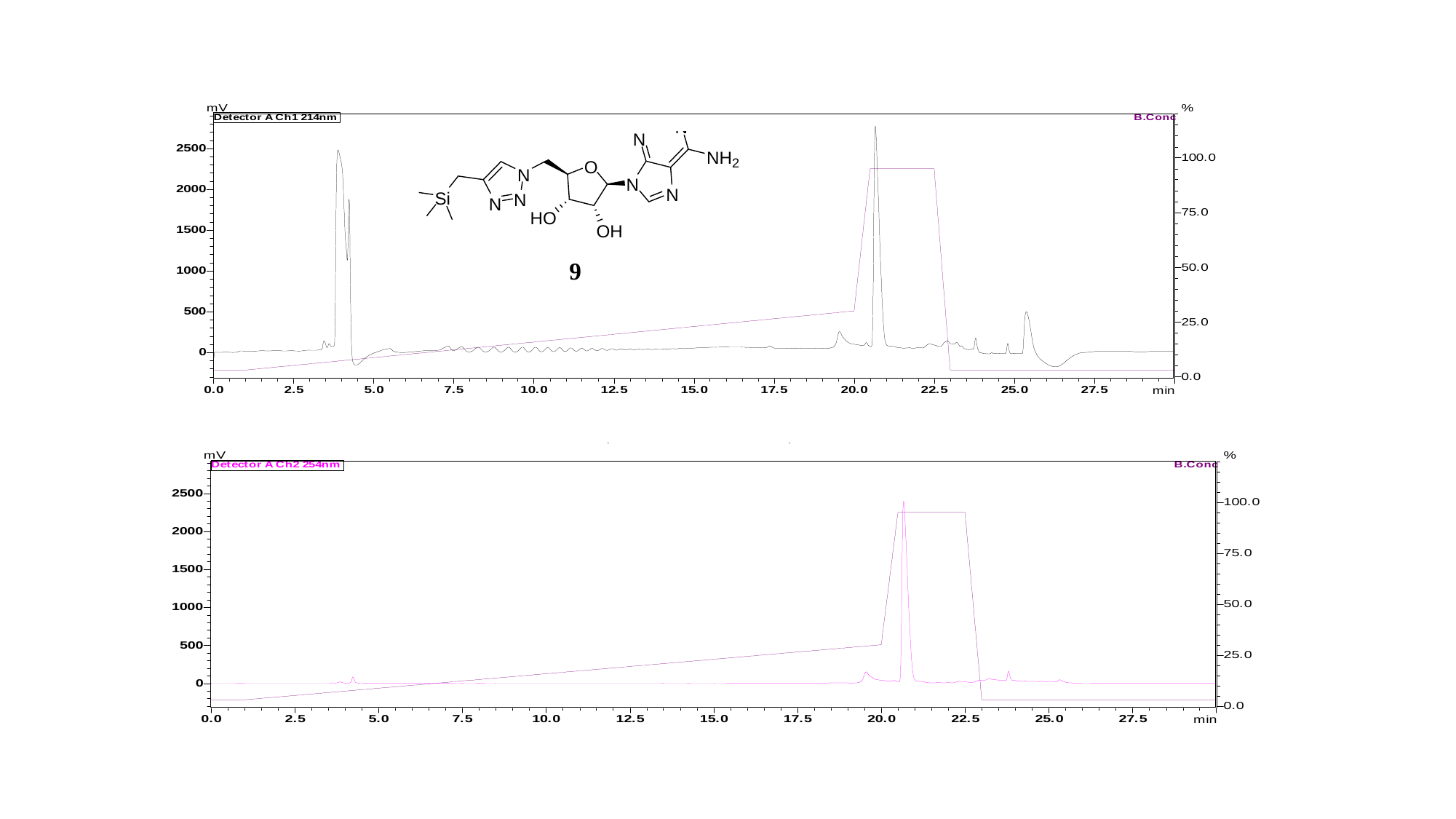

9

## Slide 31
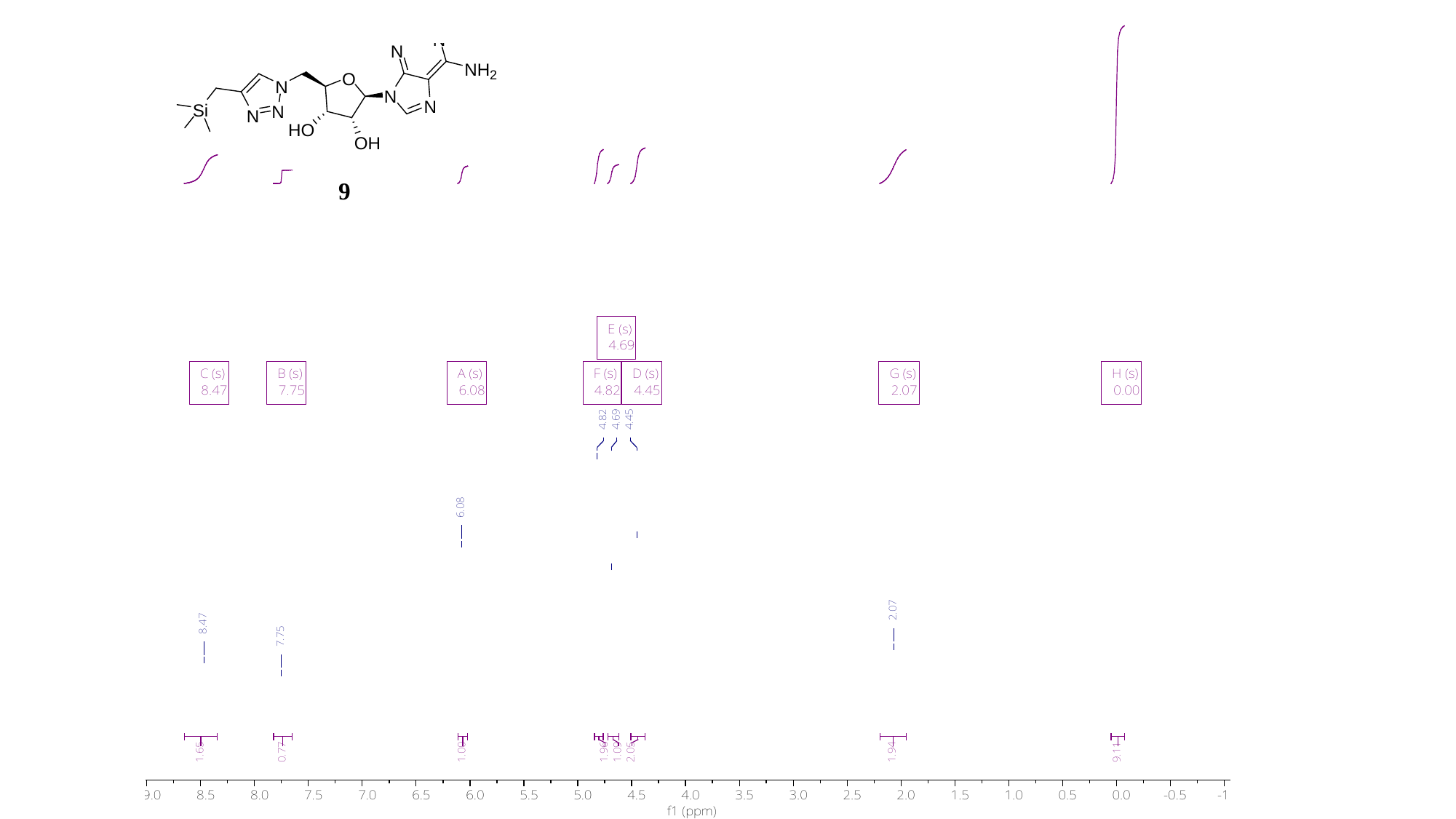

9

## Slide 32
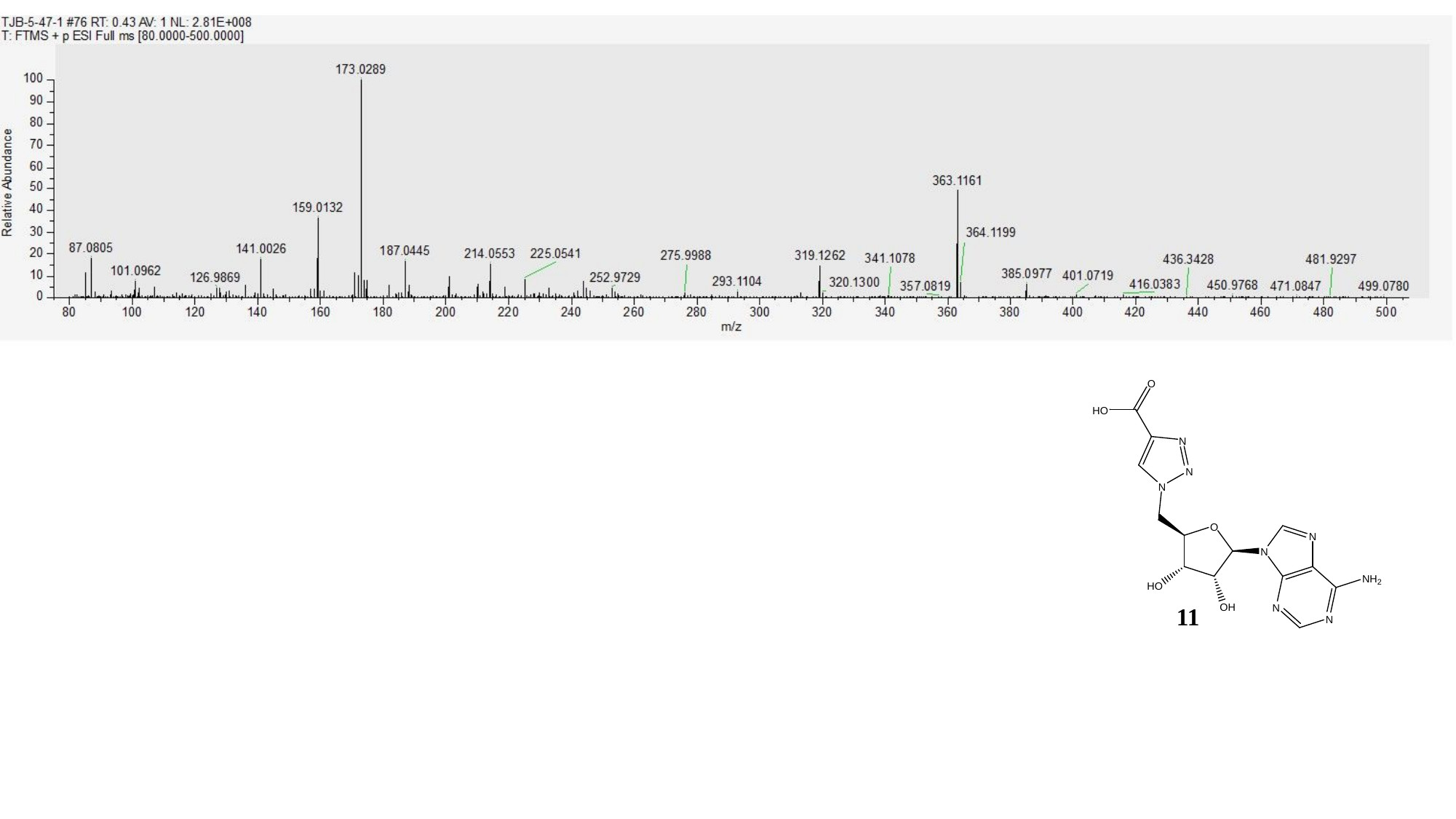

11

## Slide 33
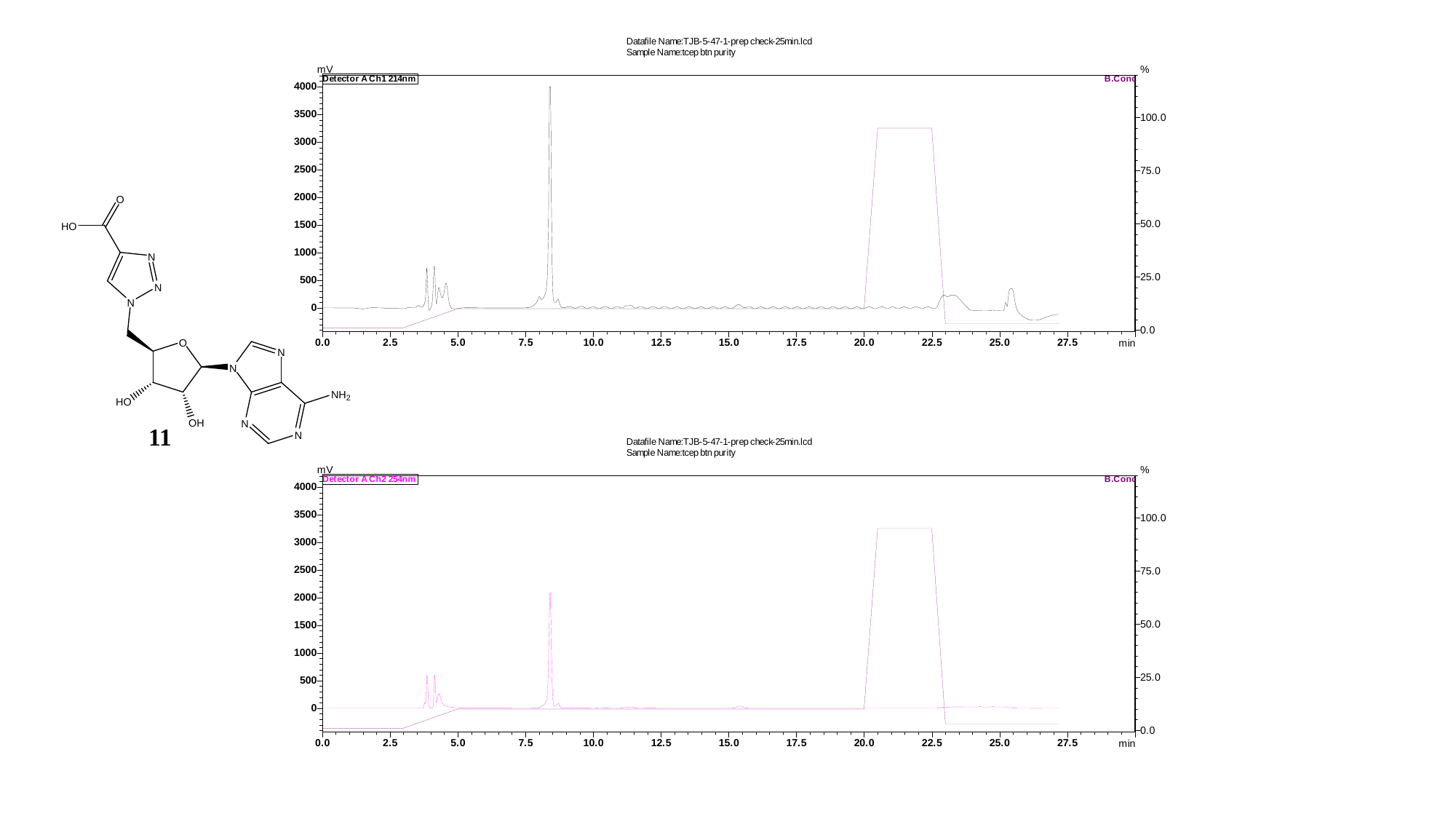

11

## Slide 34
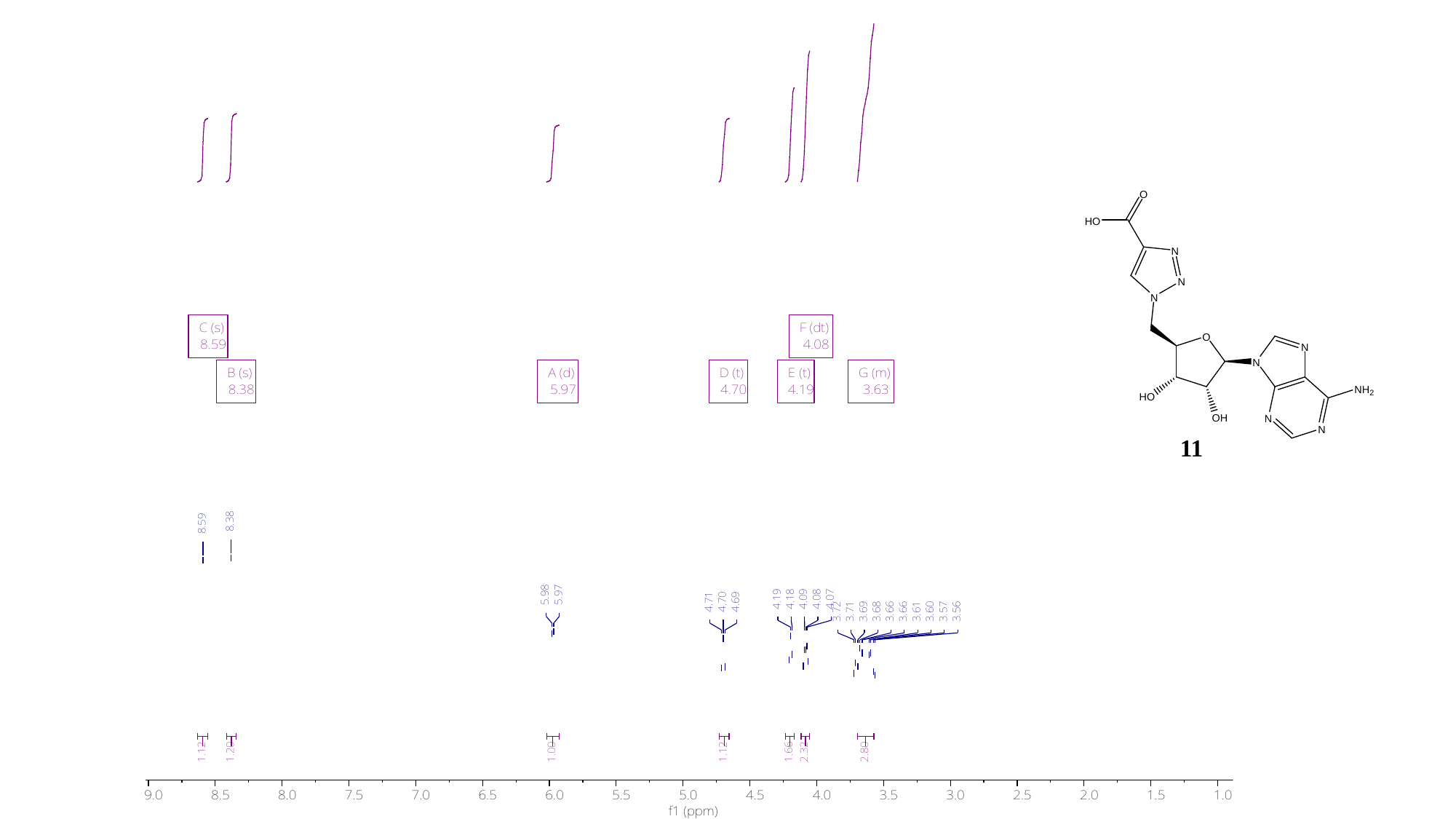

11

## Slide 35
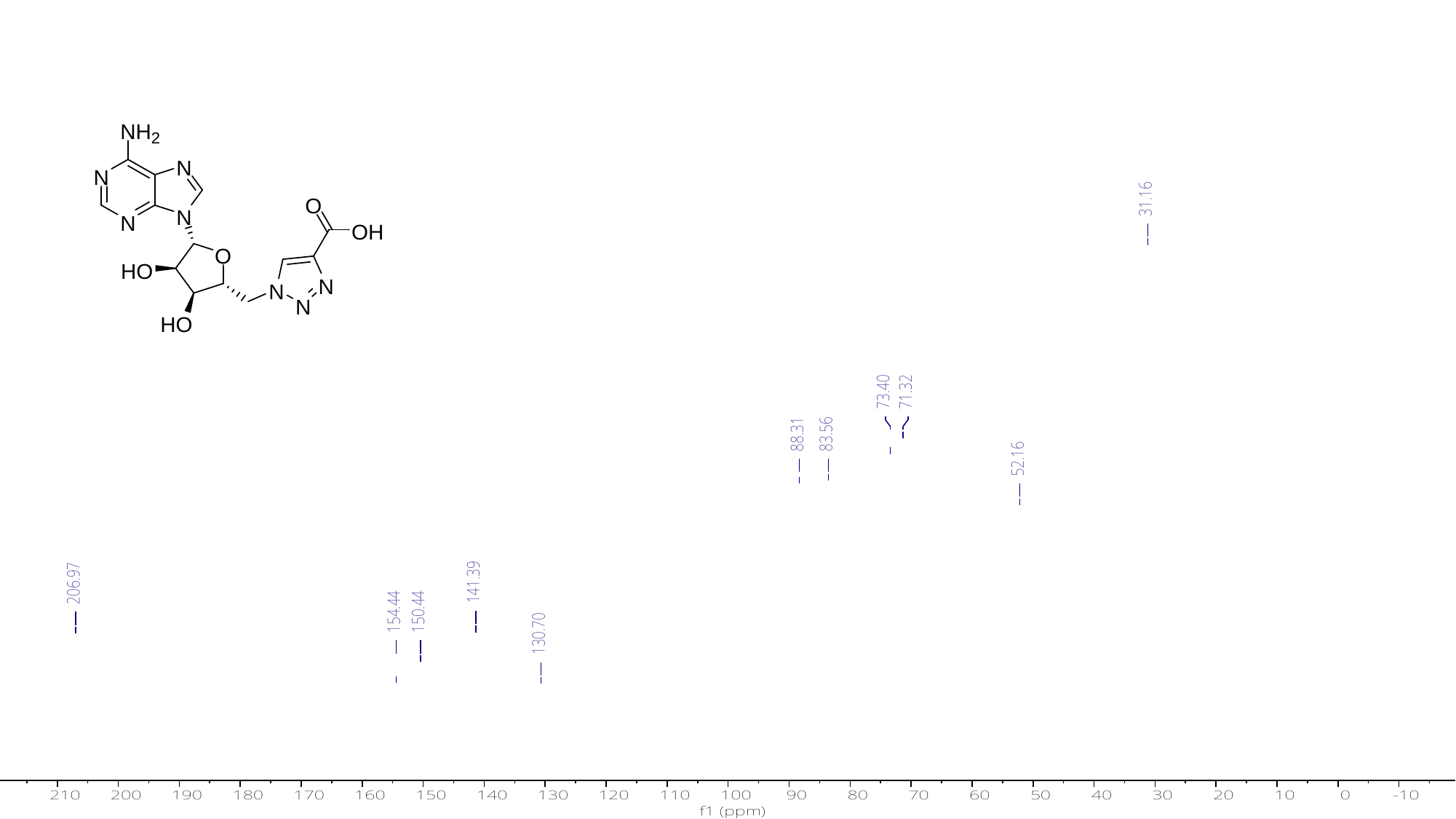

## Slide 36
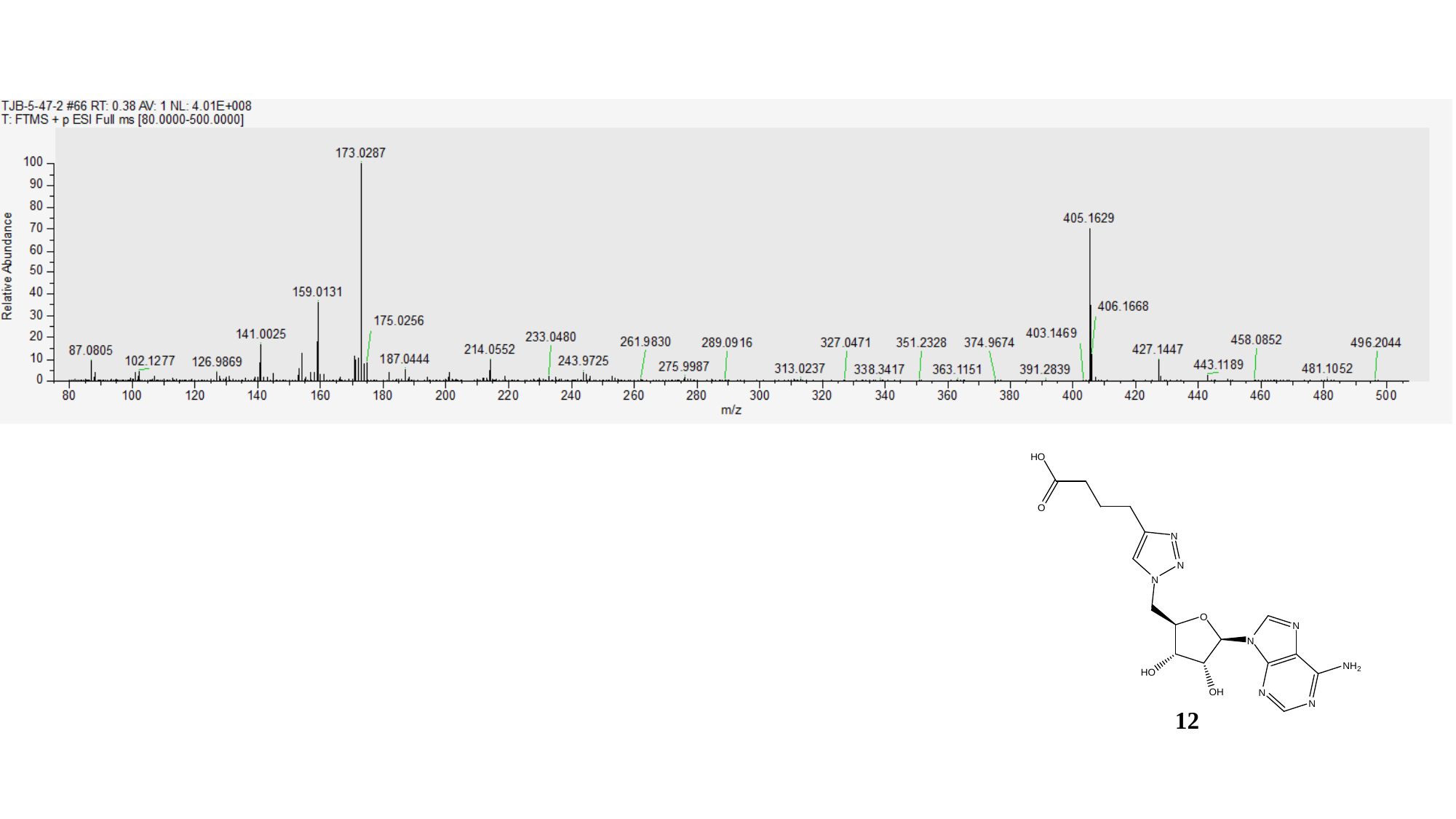

12

## Slide 37
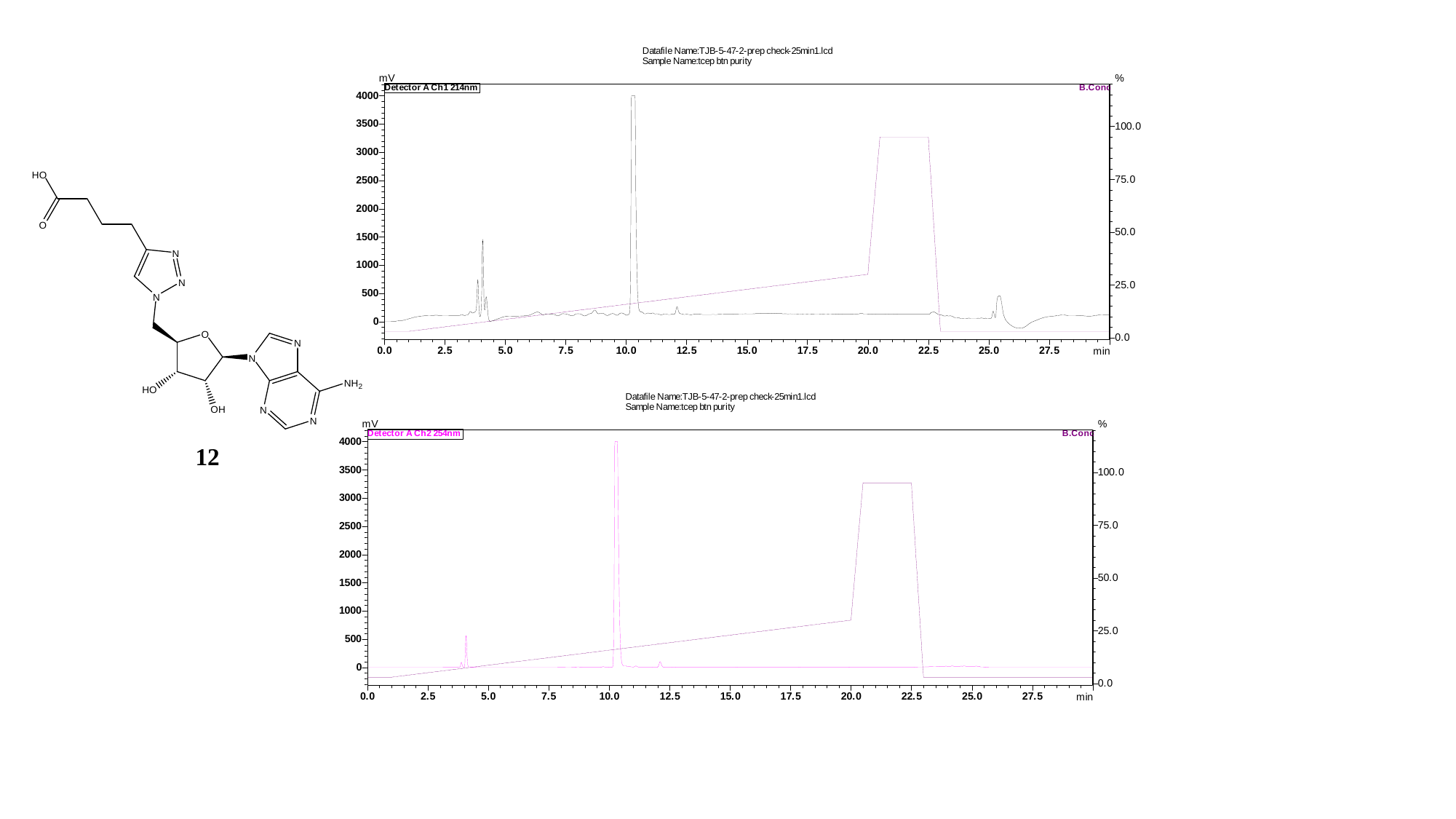

12

## Slide 38
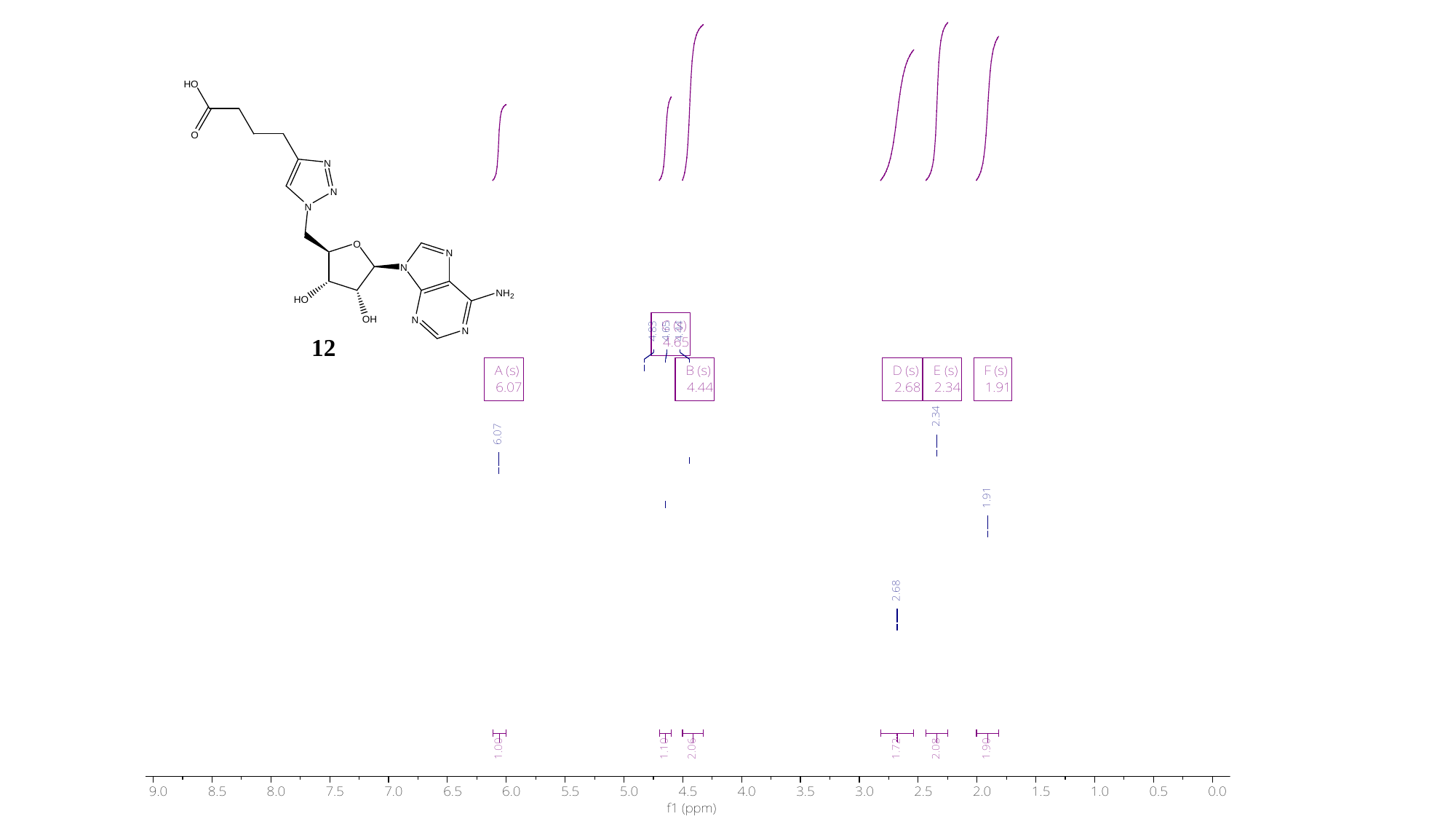

12

## Slide 39
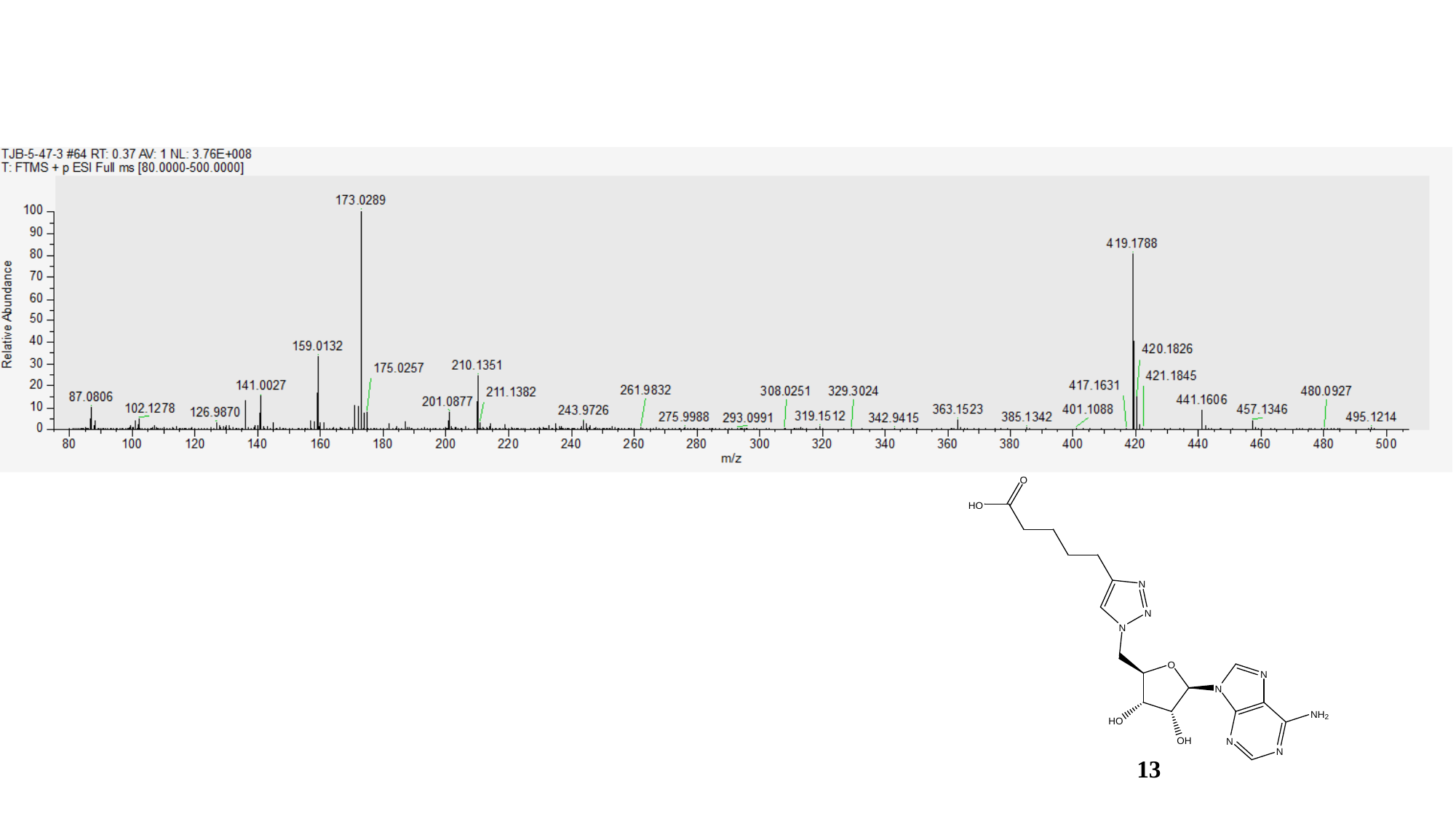

13

## Slide 40
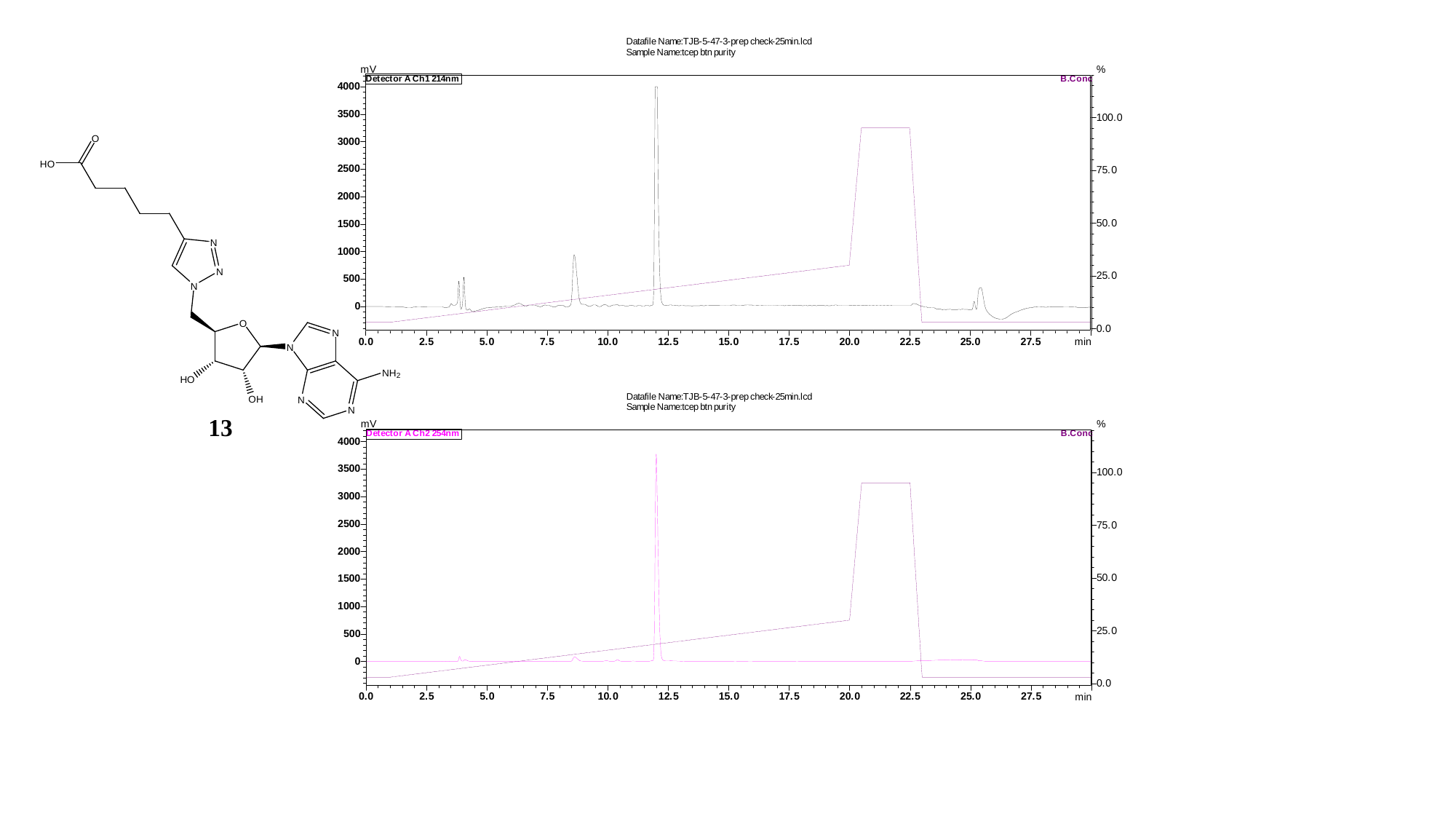

13

## Slide 41
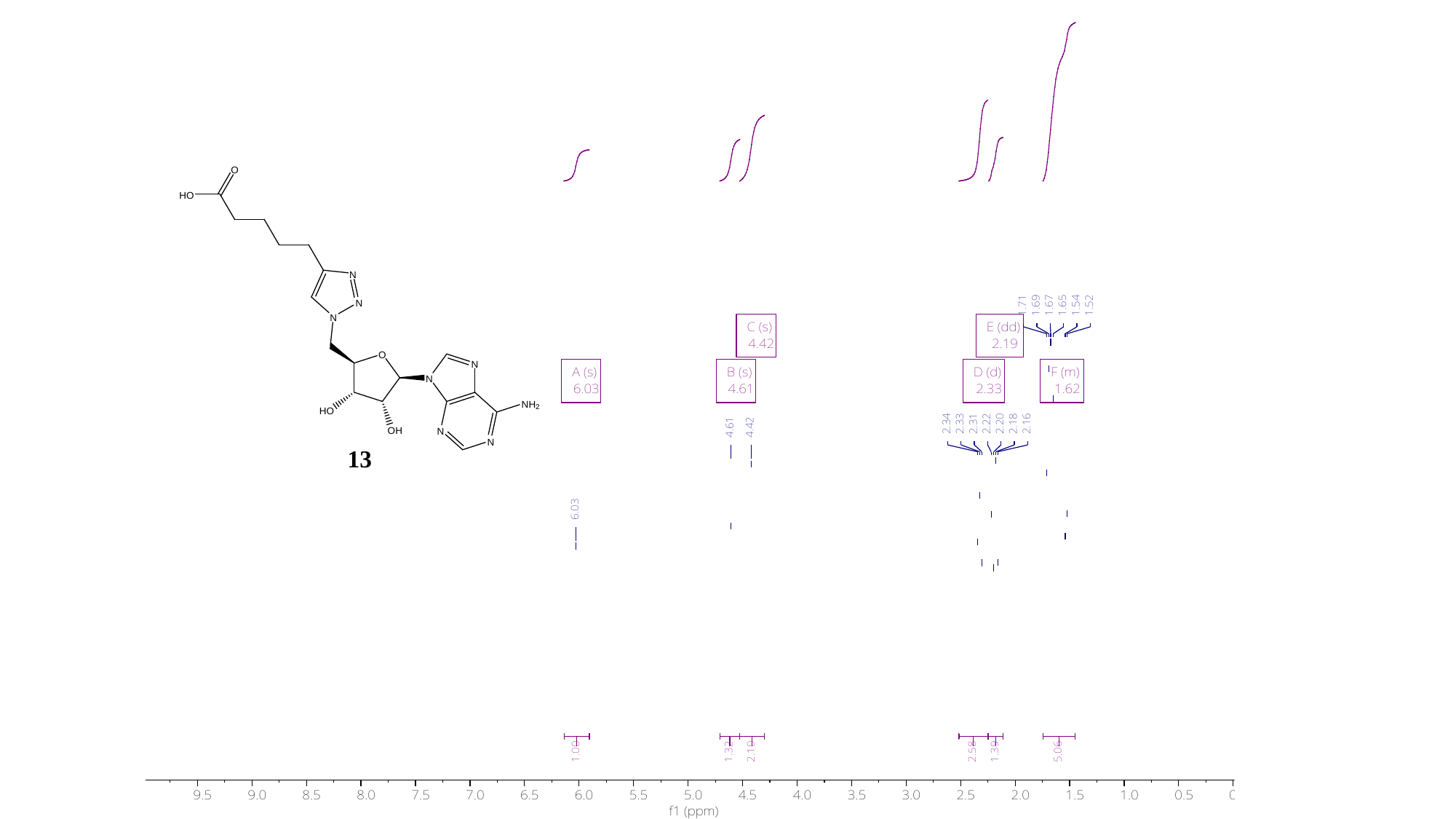

13

## Slide 42
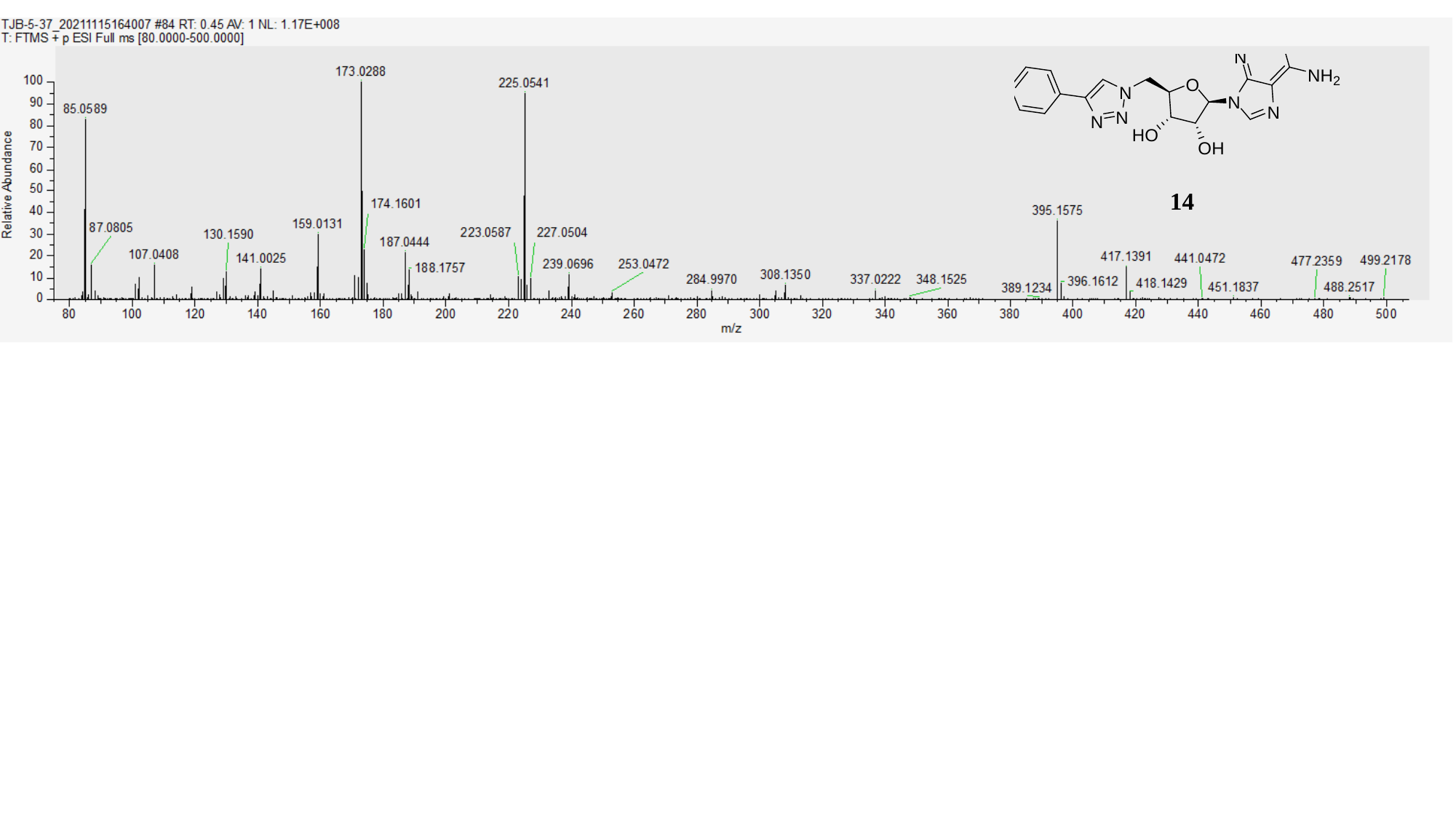

14

## Slide 43
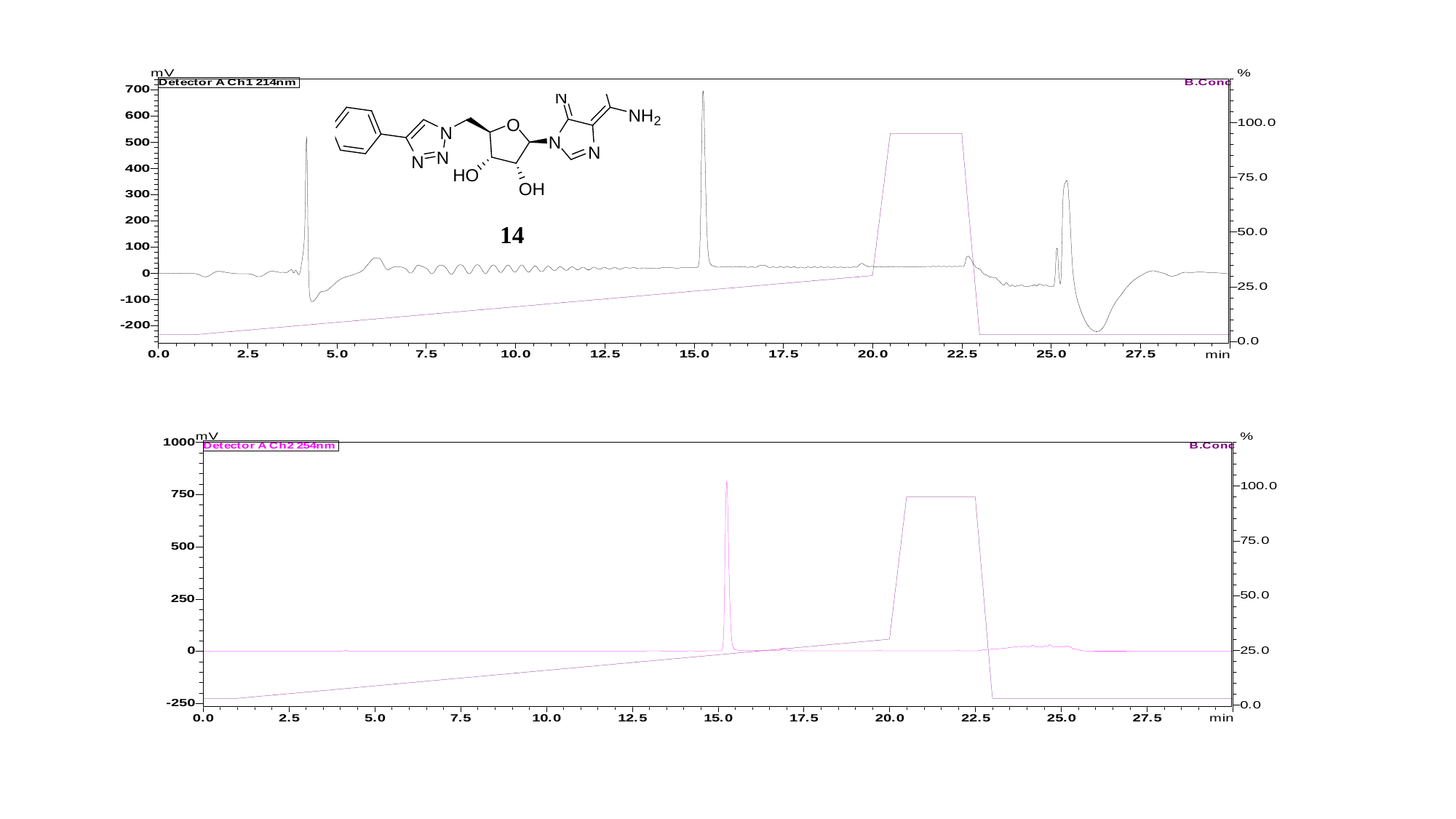

14

## Slide 44
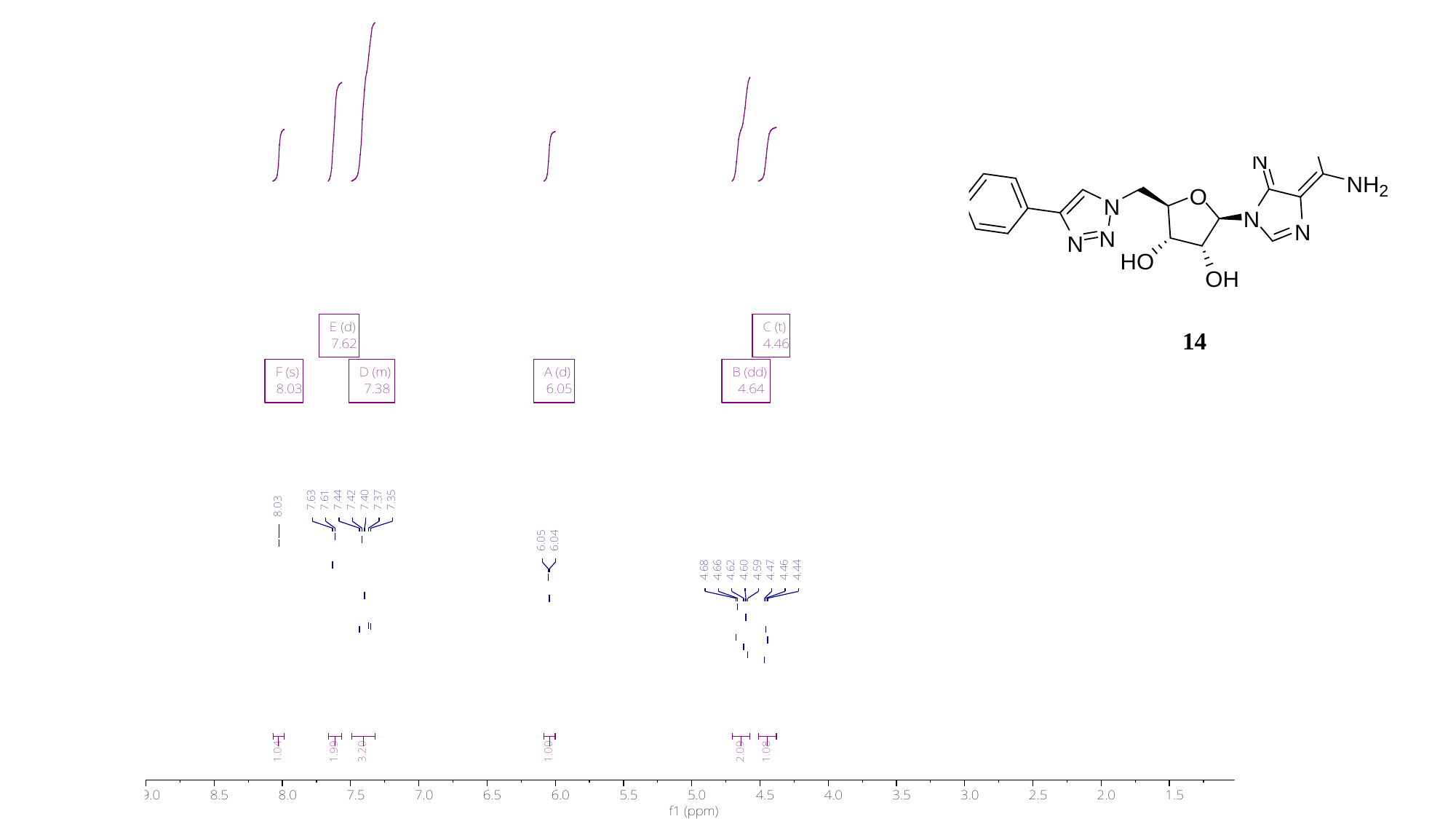

14

## Slide 45
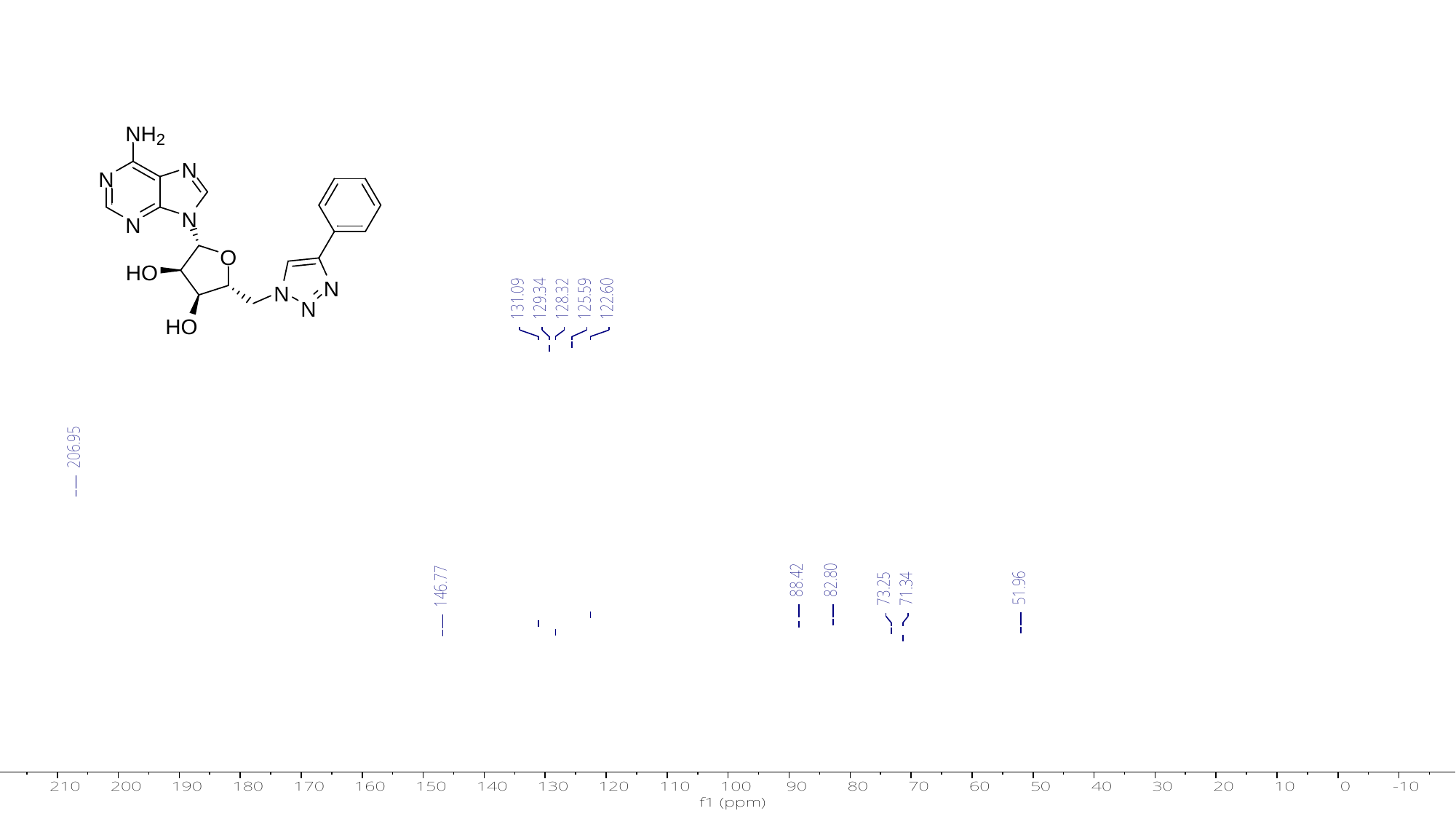

## Slide 46
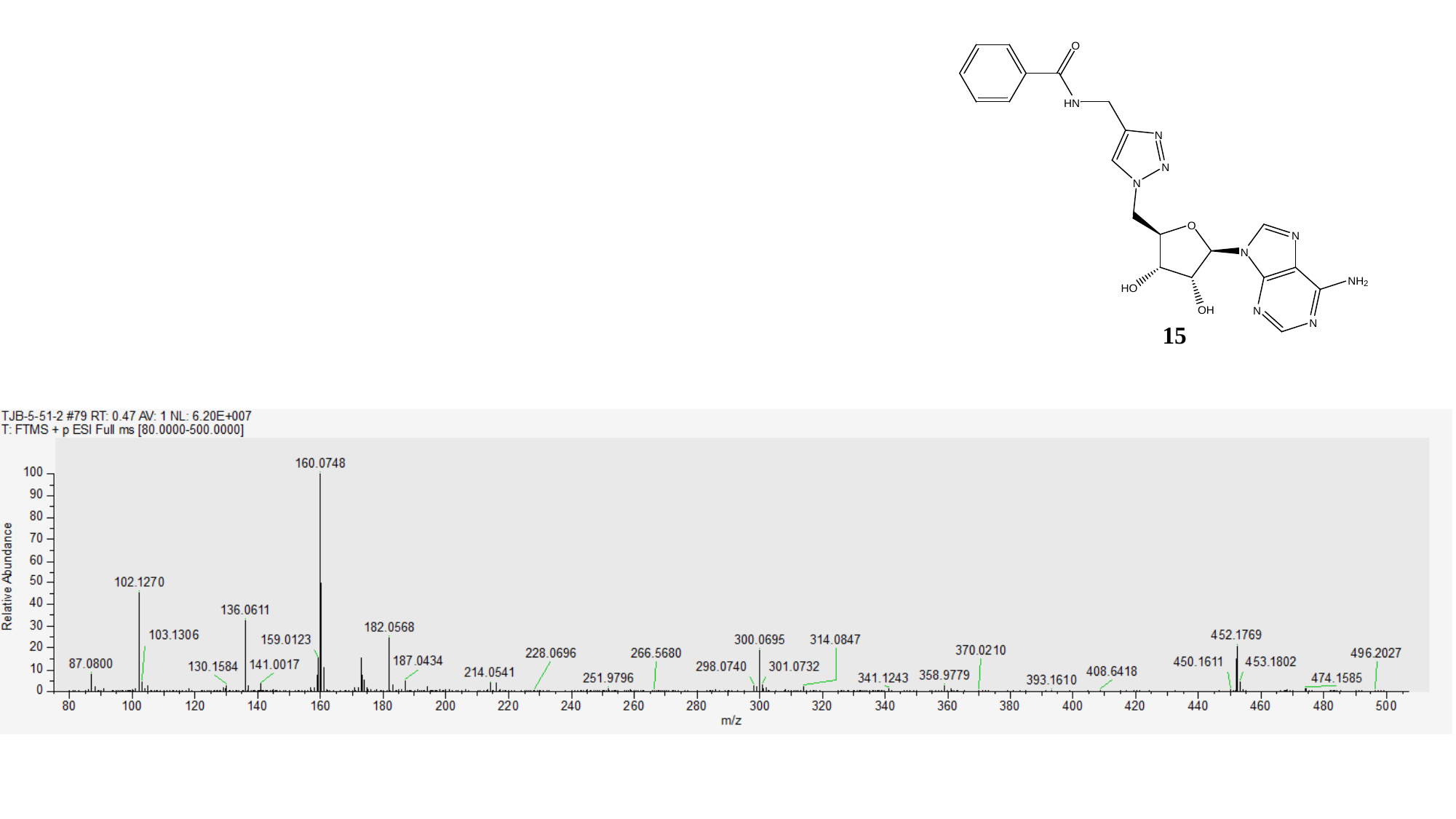

15

## Slide 47
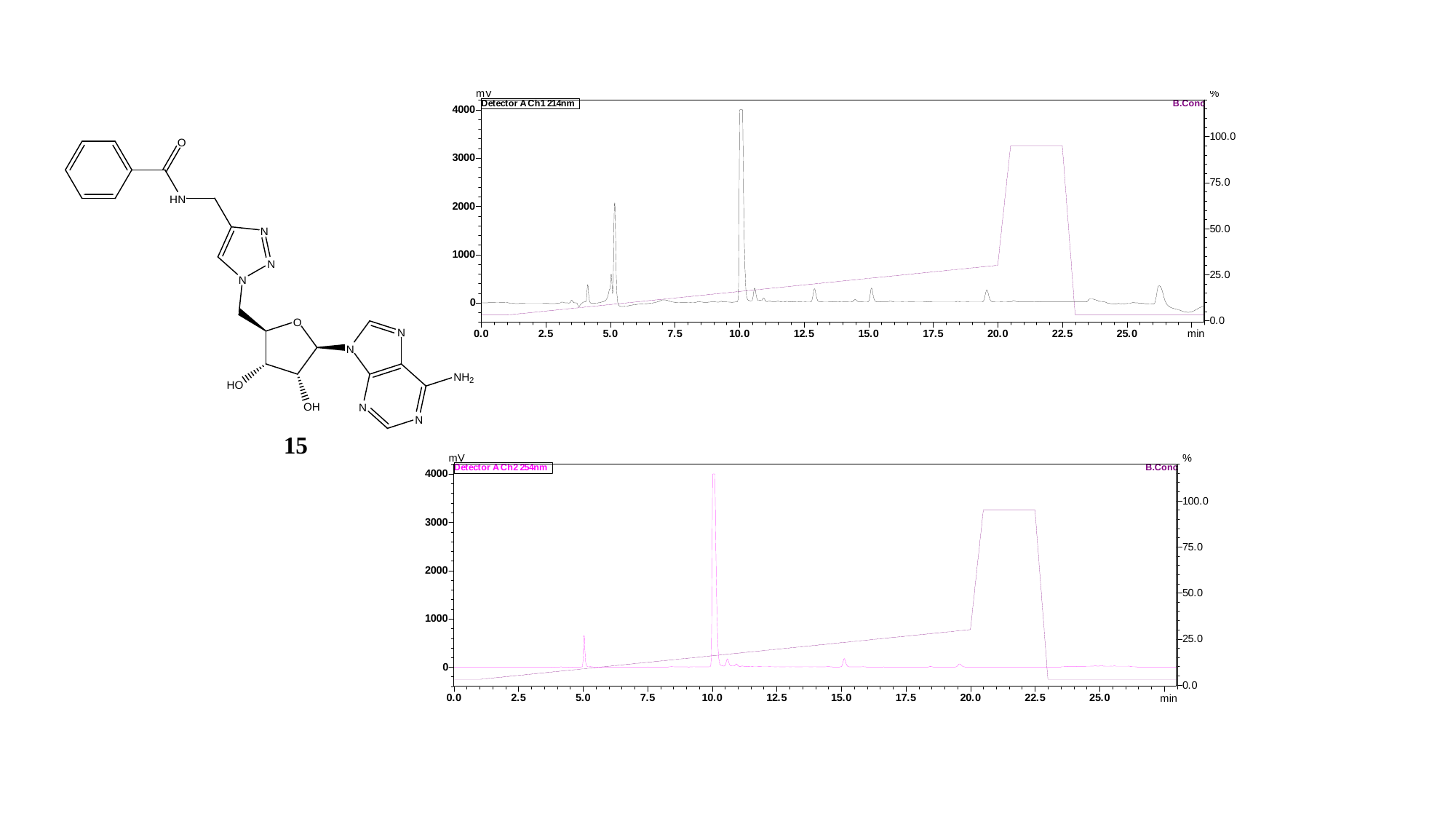

15

## Slide 48
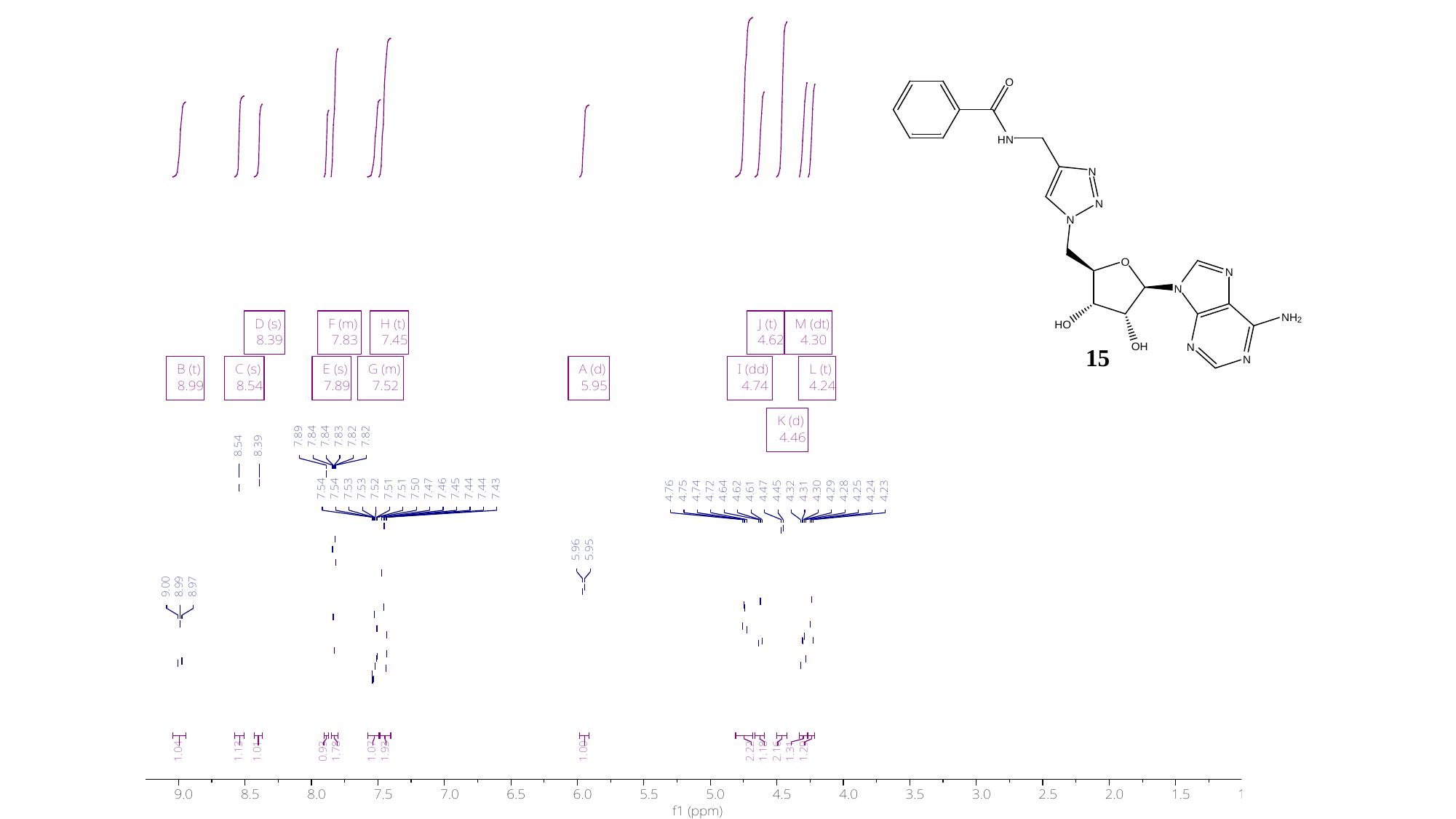

15

## Slide 49
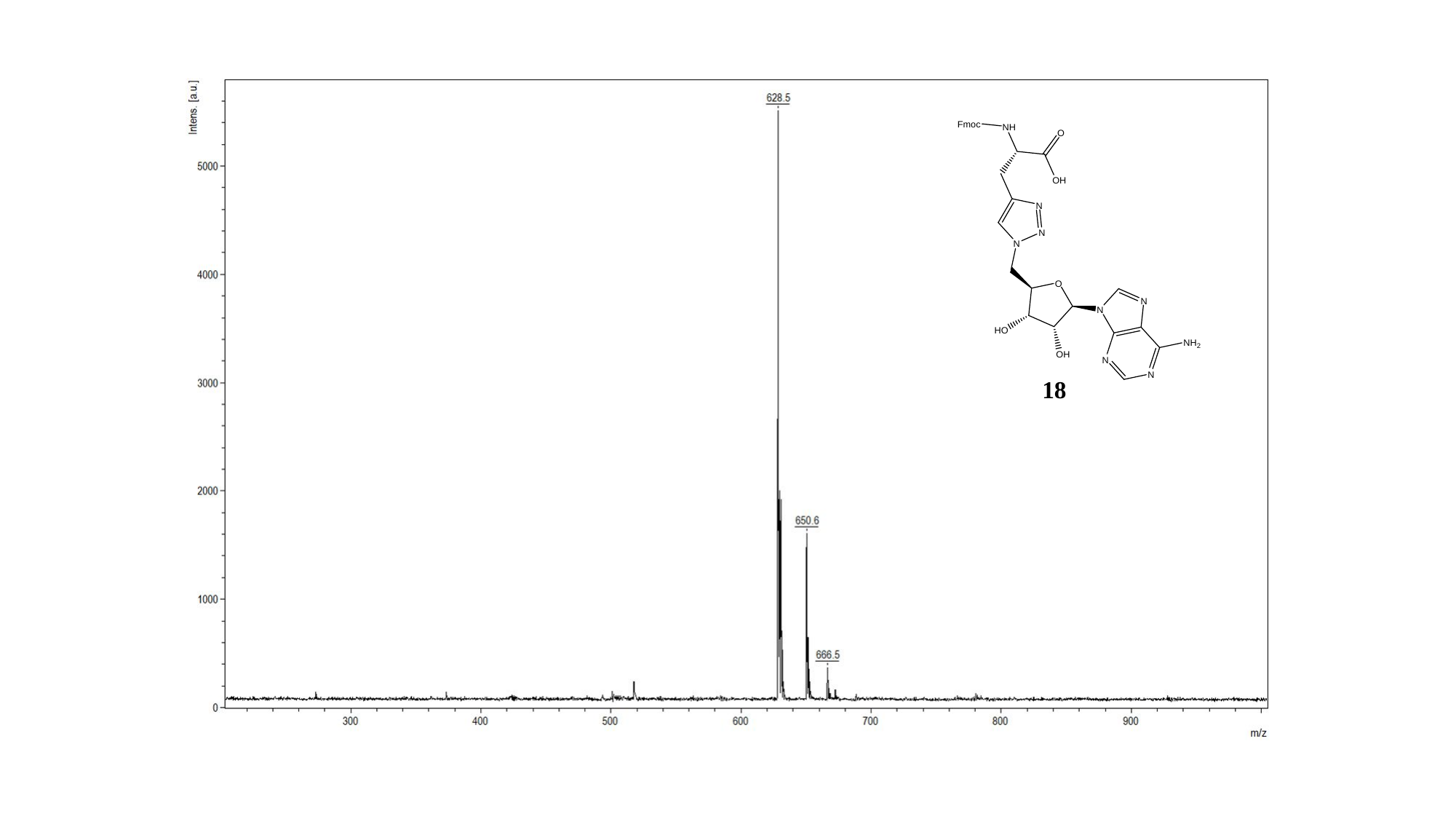

18

## Slide 50
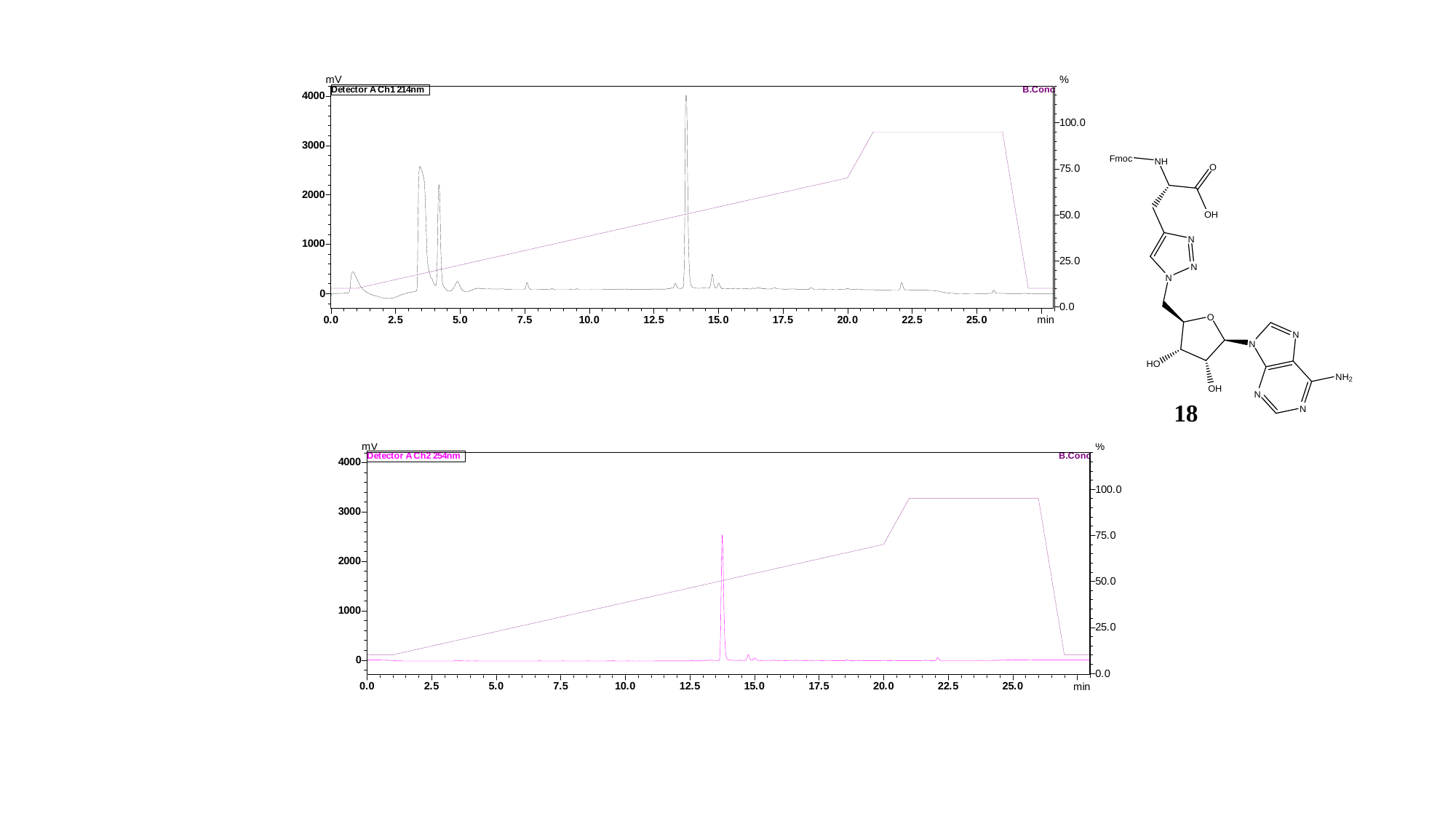

18

## Slide 51
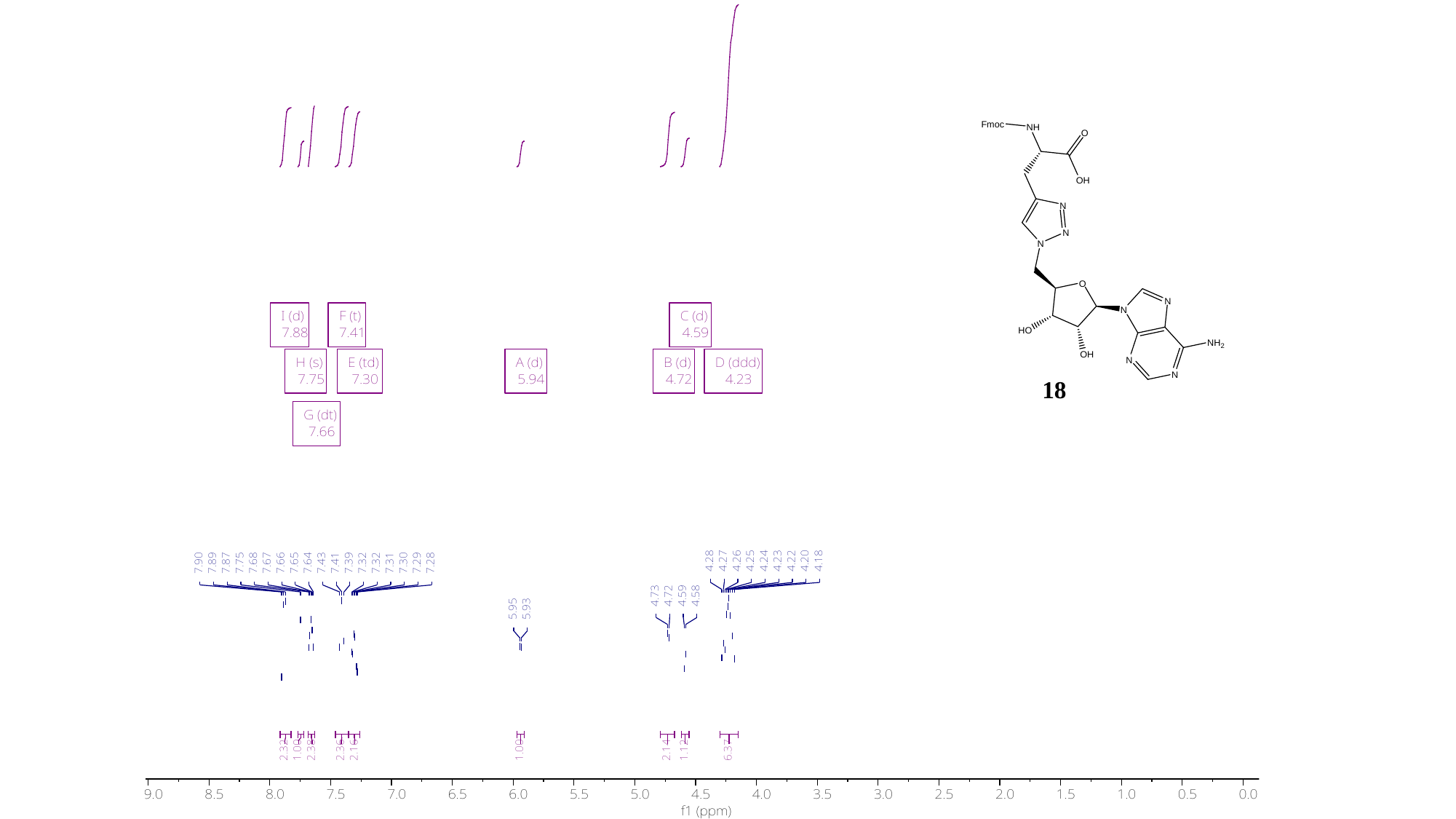

18

## Slide 52
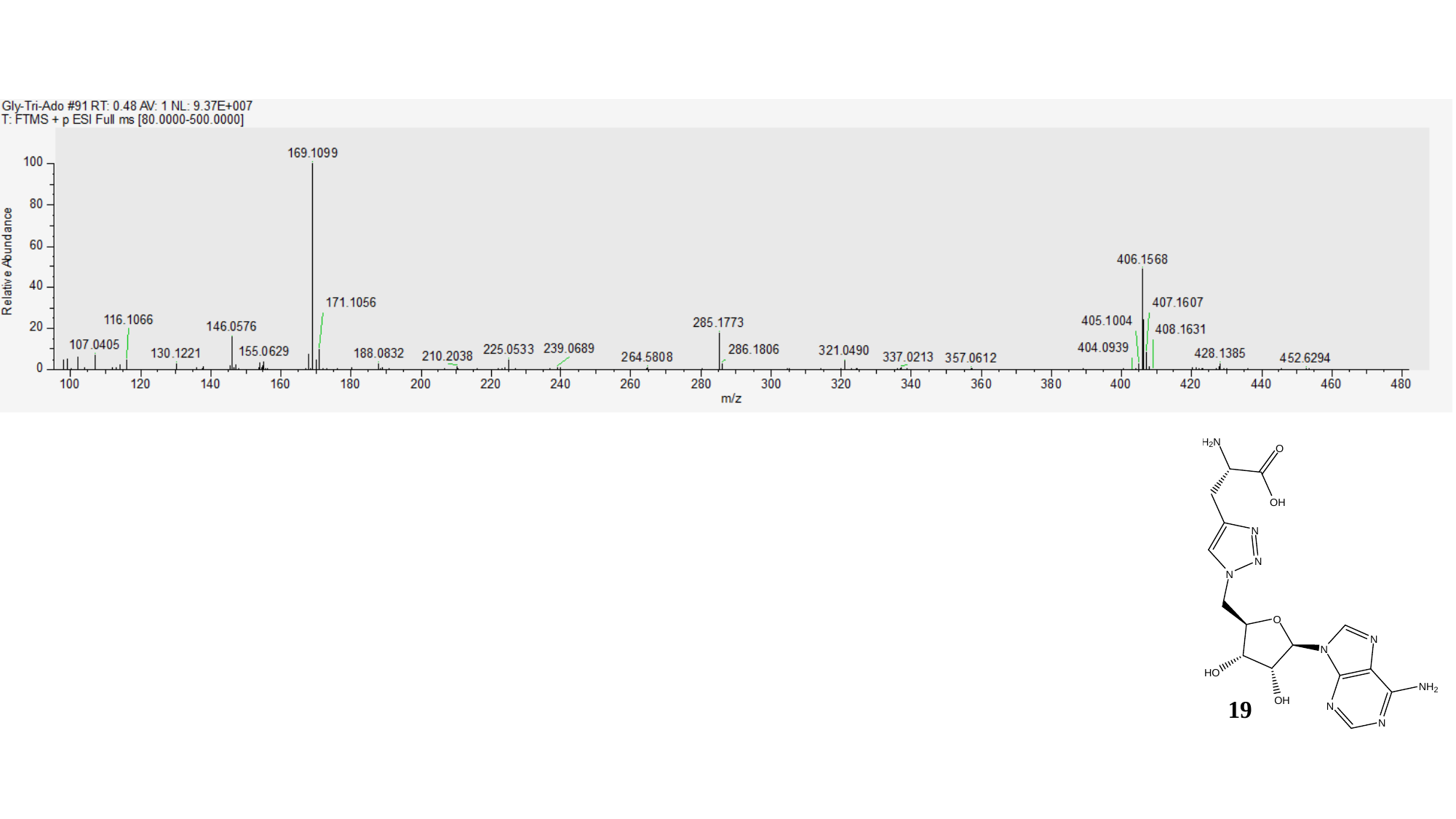

19

## Slide 53
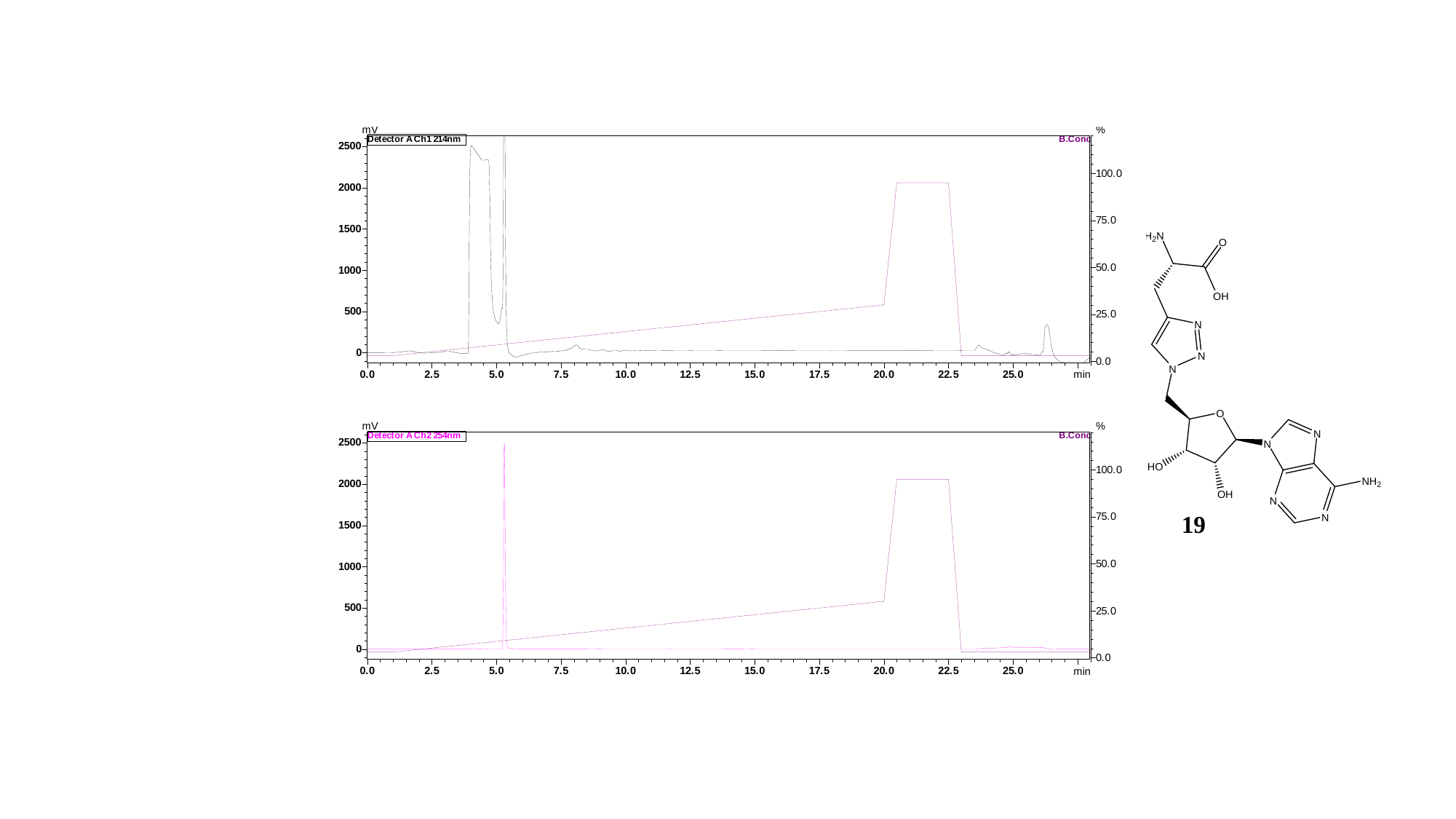

19

## Slide 54
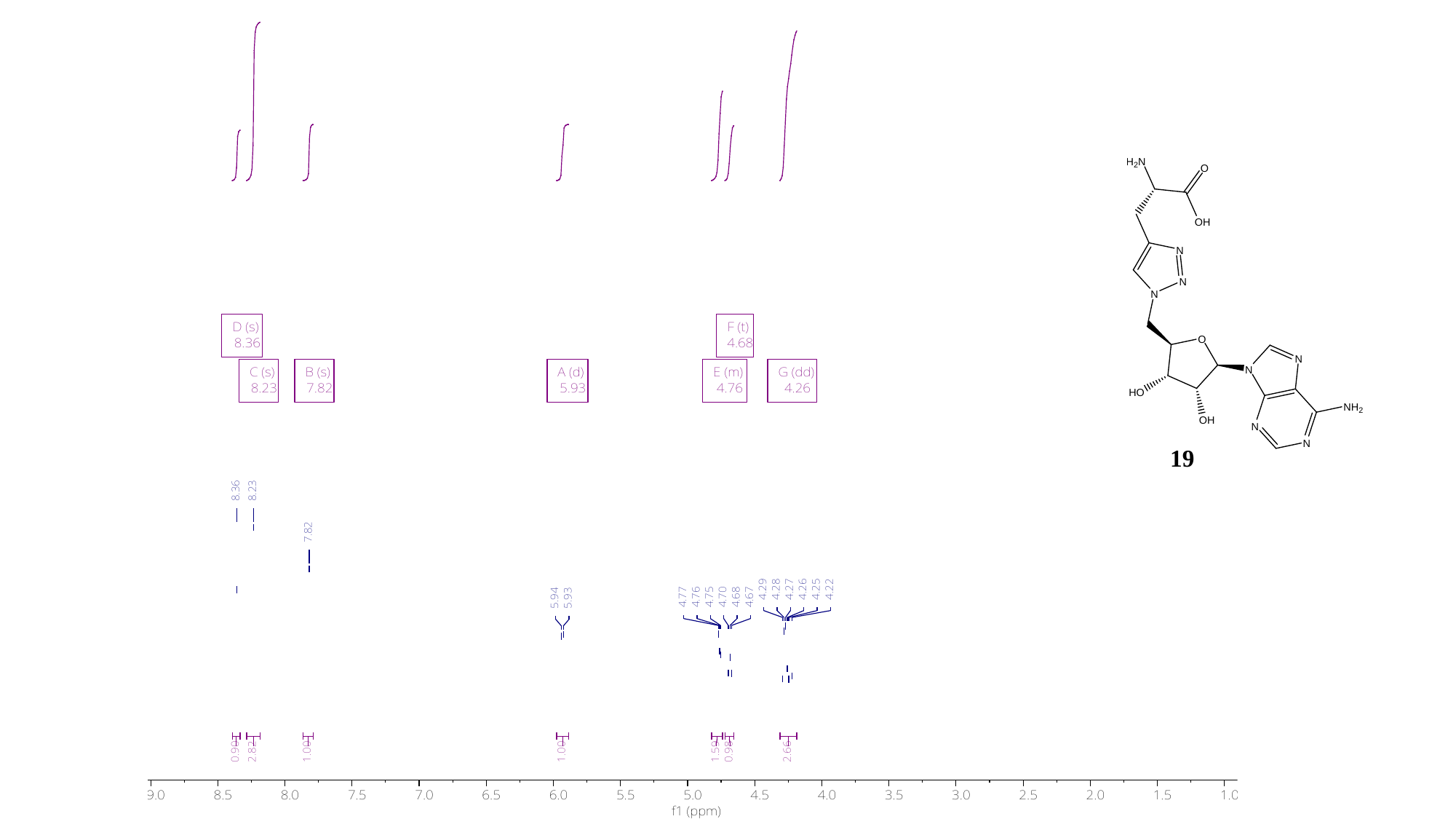

19

## Slide 55
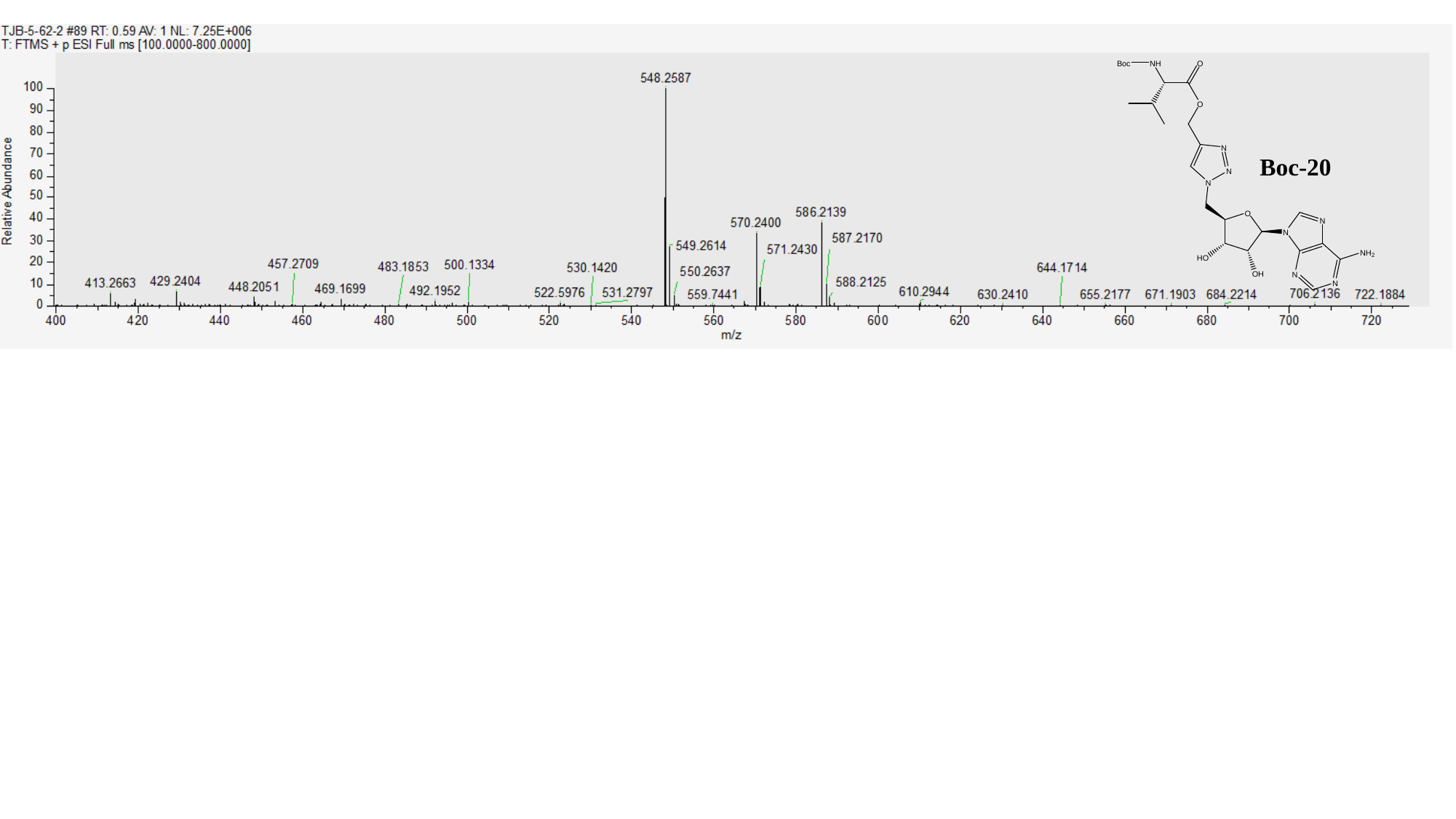

Boc-20

## Slide 56
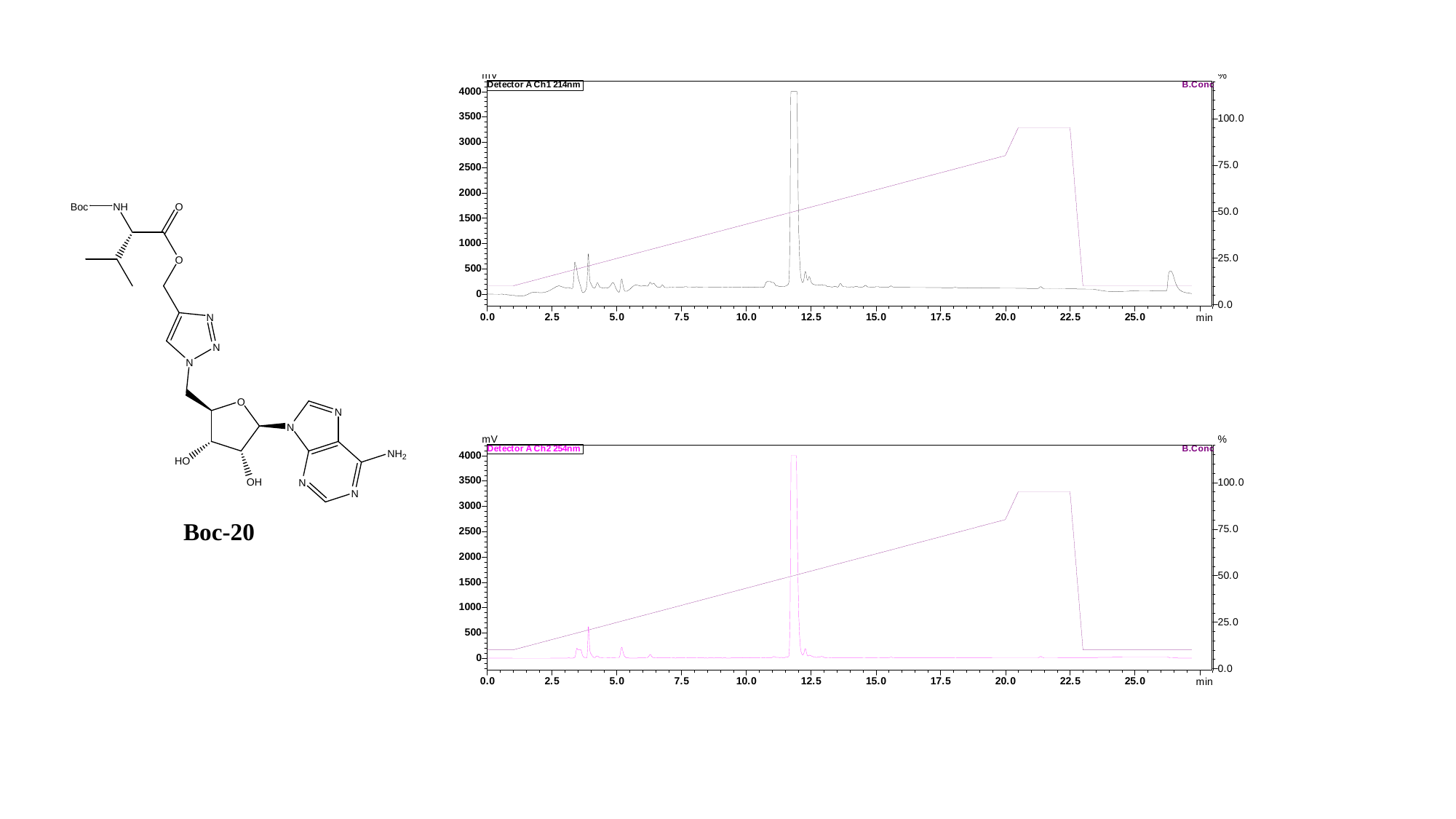

Boc-20

## Slide 57
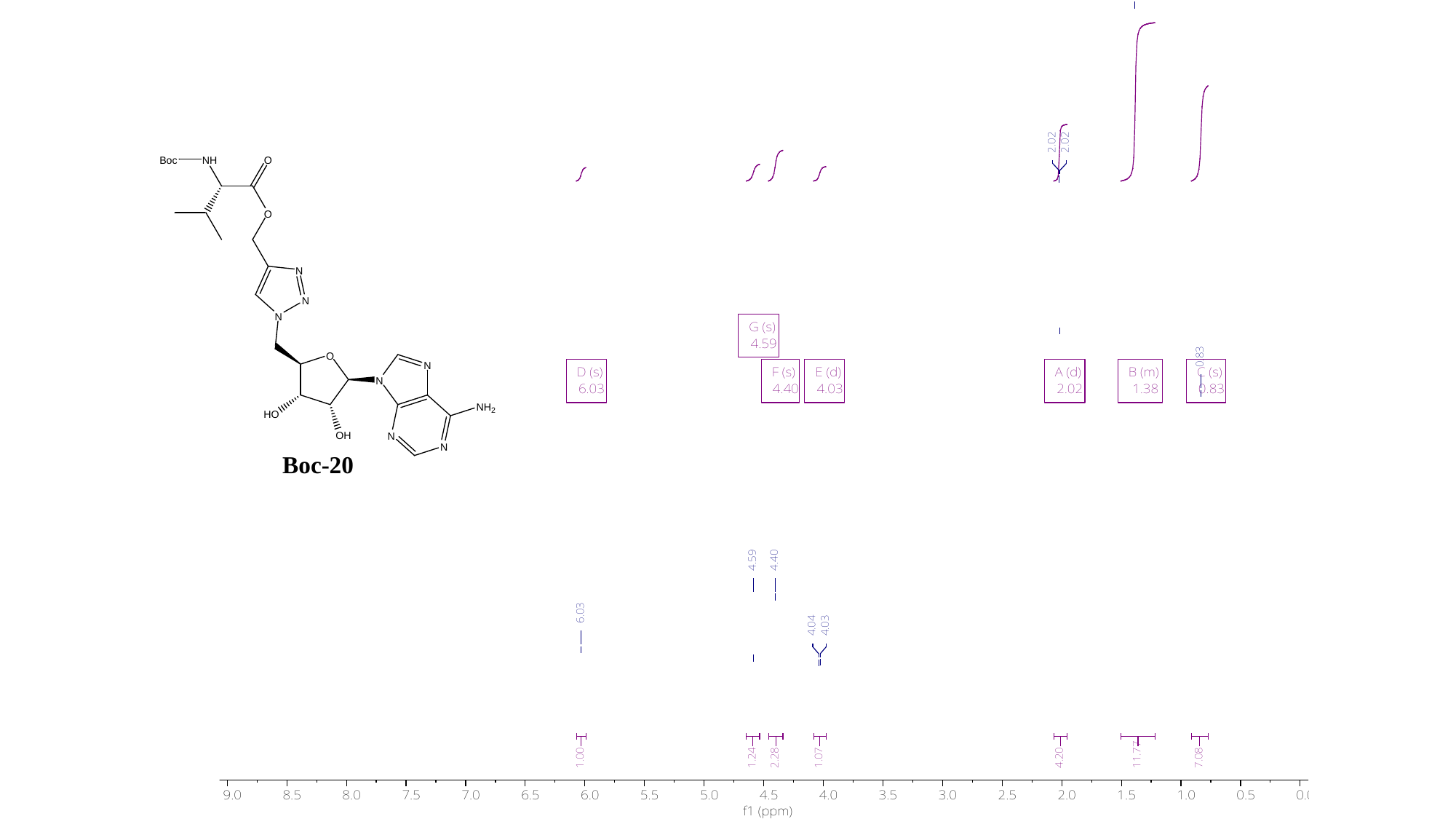

Boc-20

## Slide 58
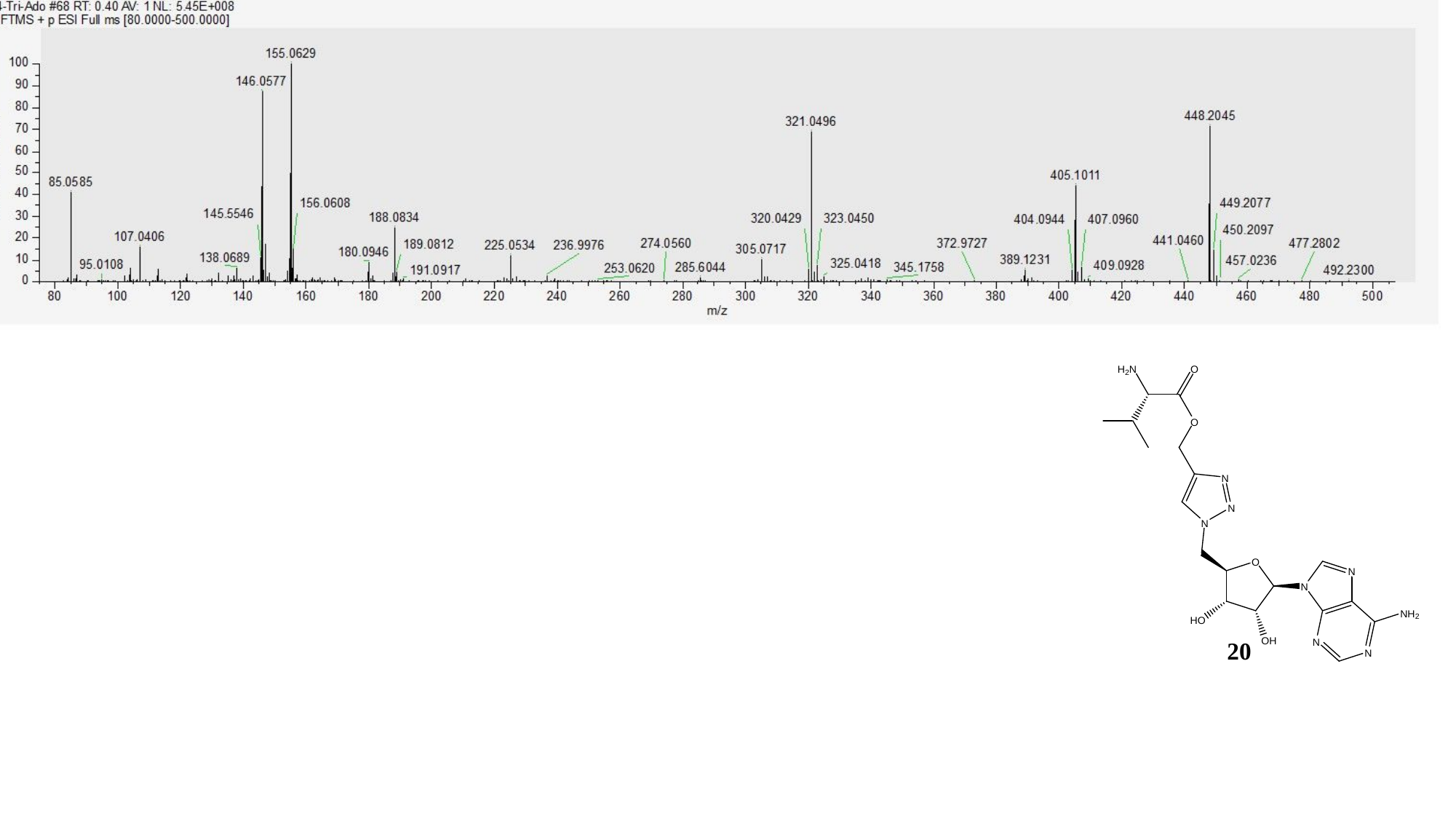

20

## Slide 59
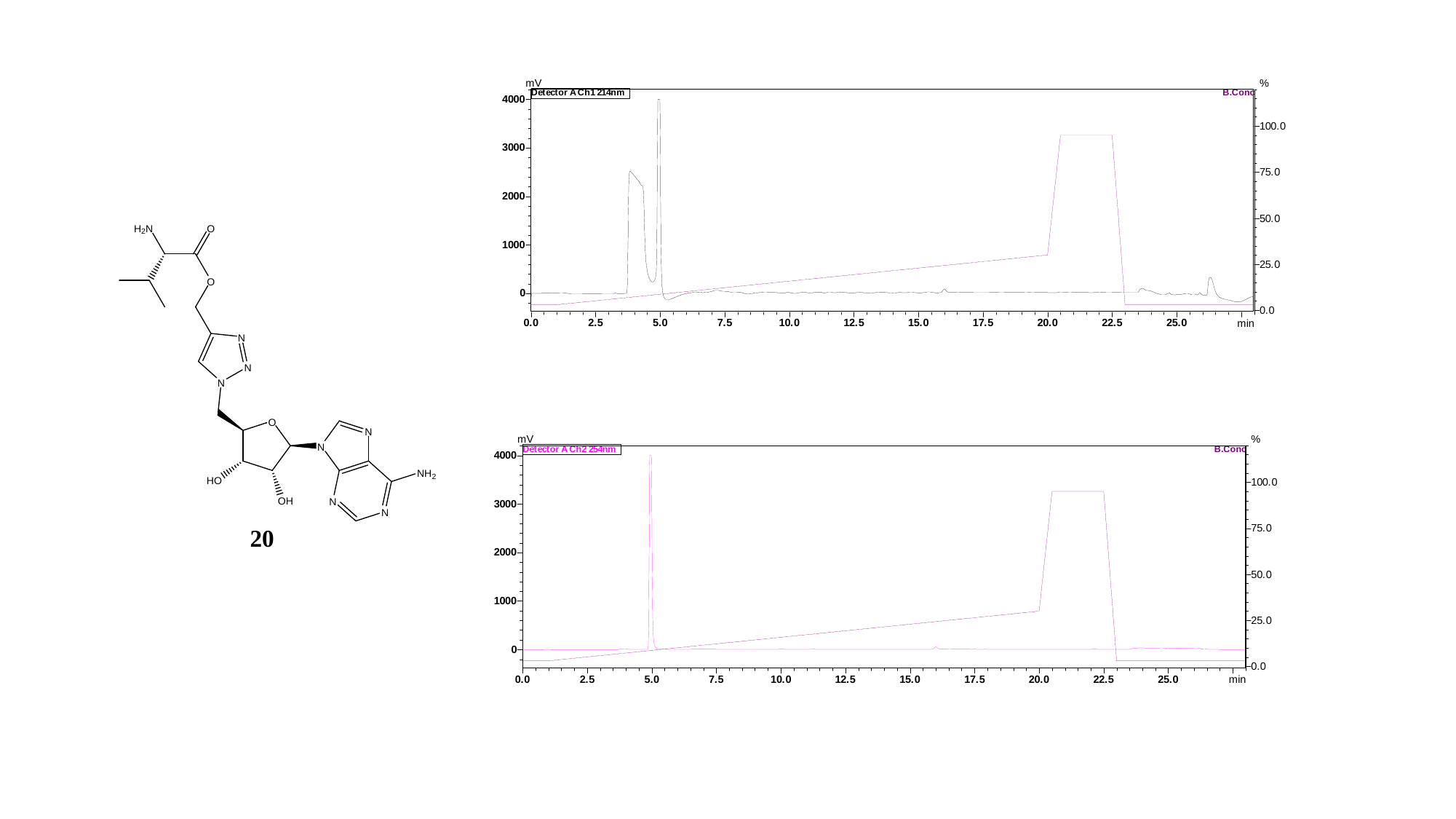

20

## Slide 60
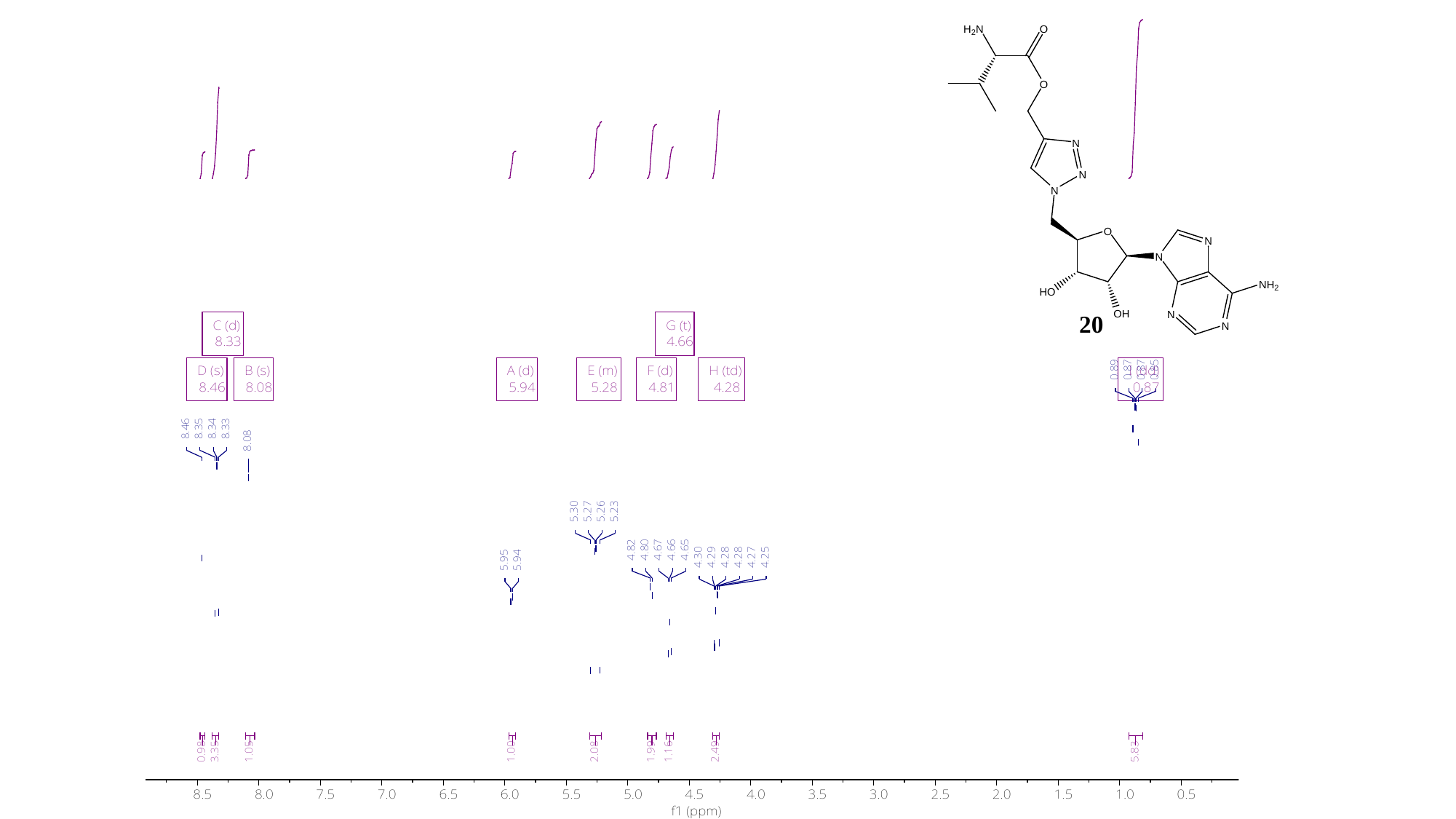

20

## Slide 61
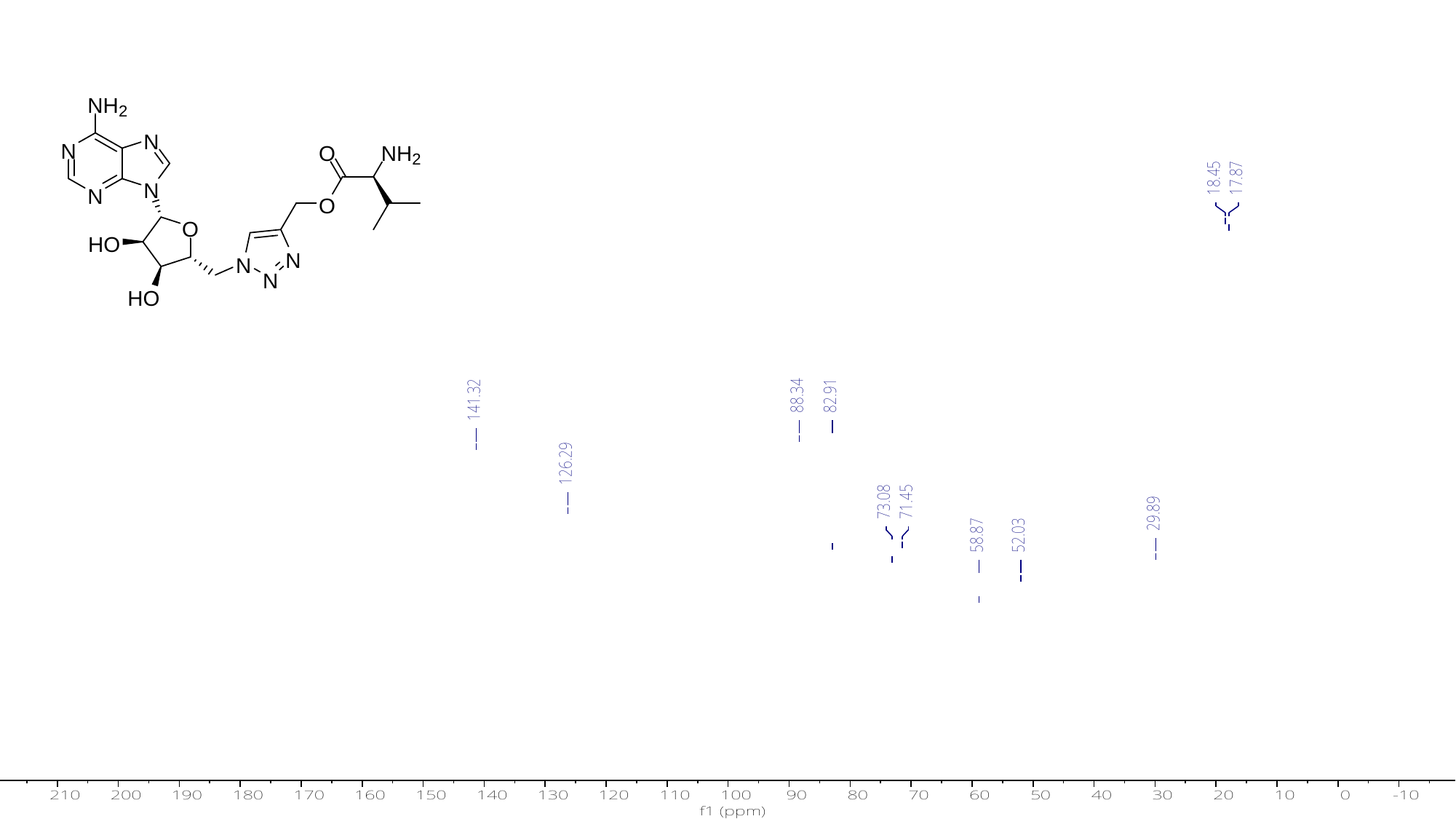

## Slide 62
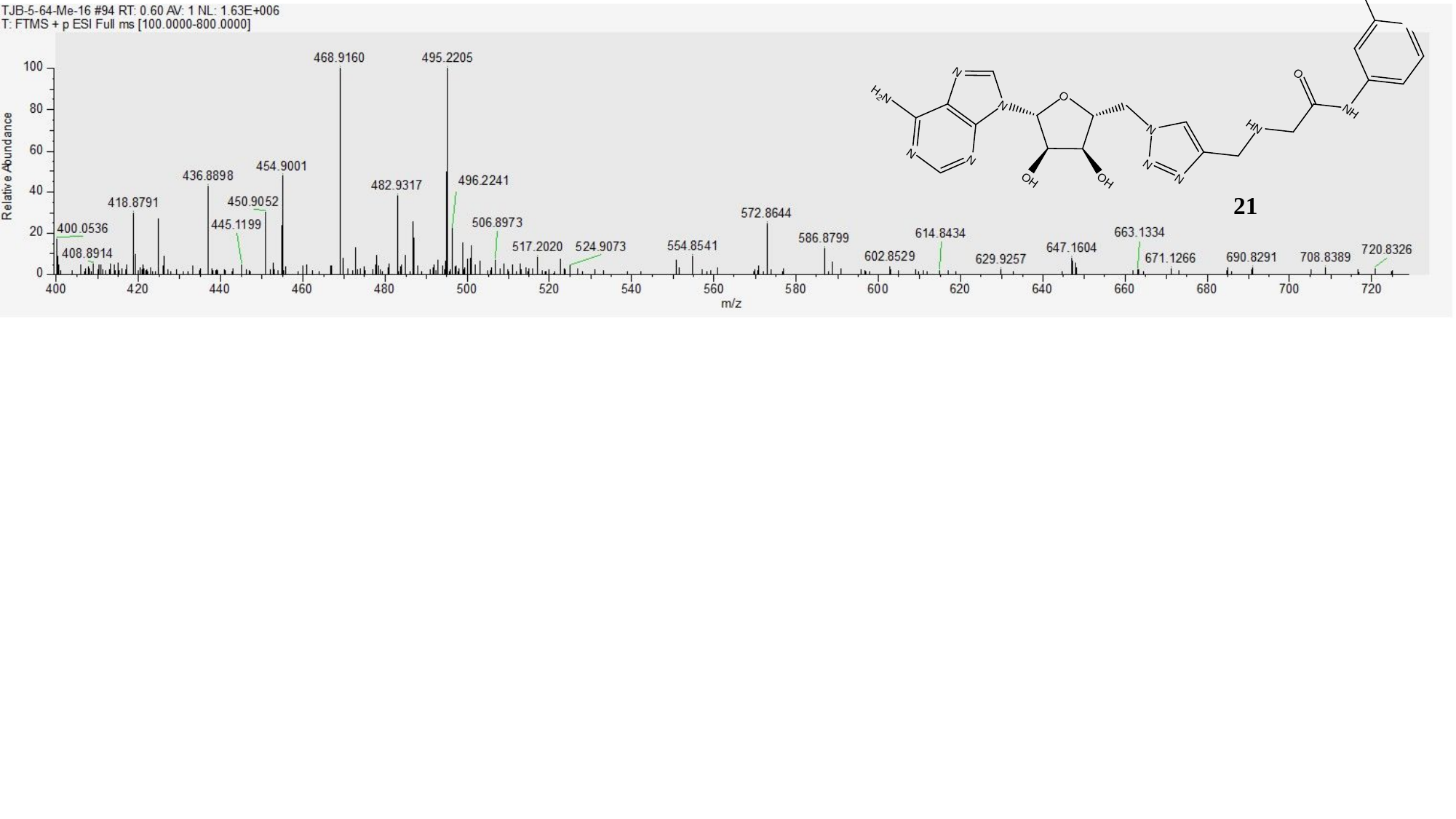

21

## Slide 63
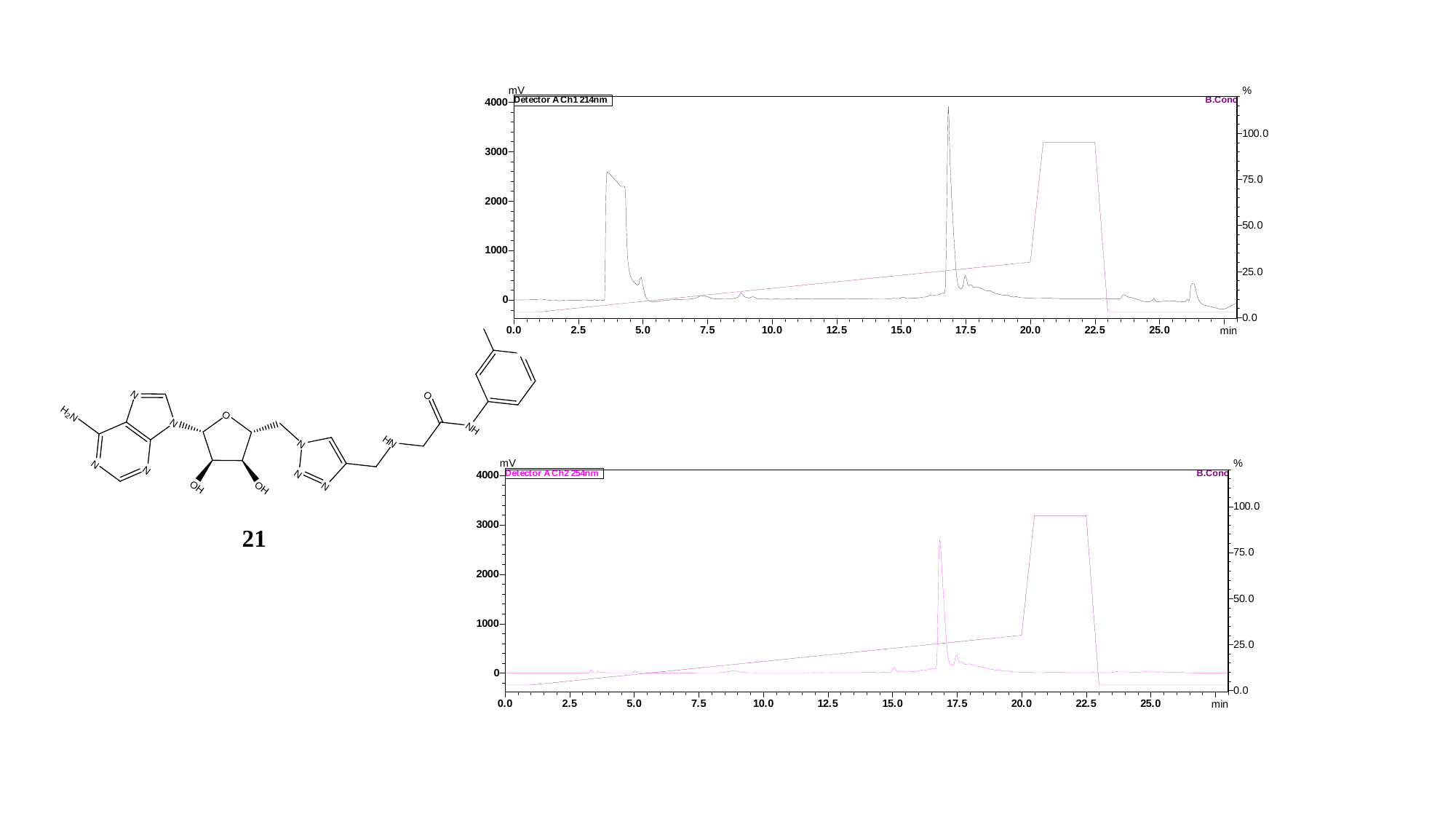

21

## Slide 64
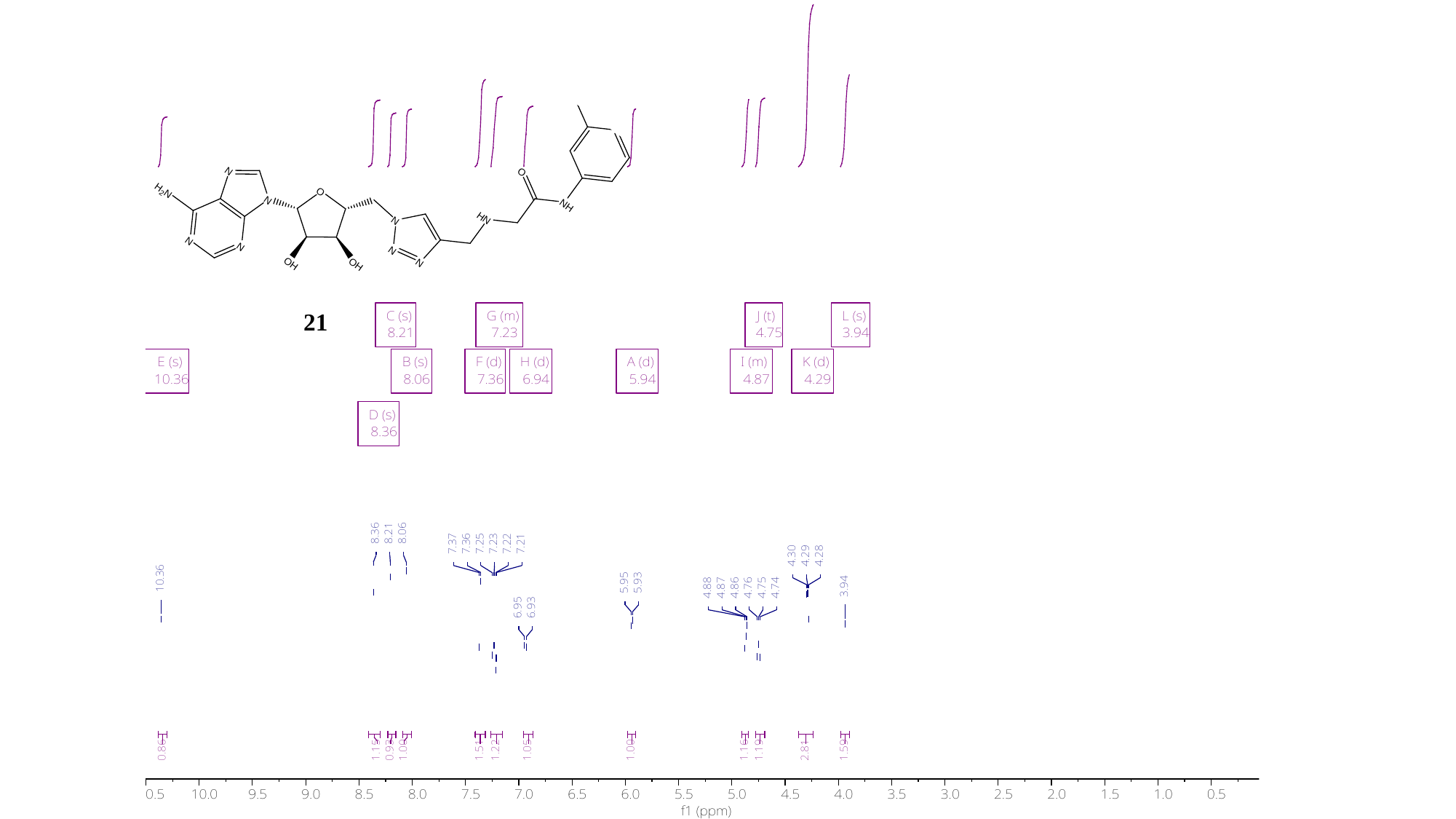

21

## Slide 65
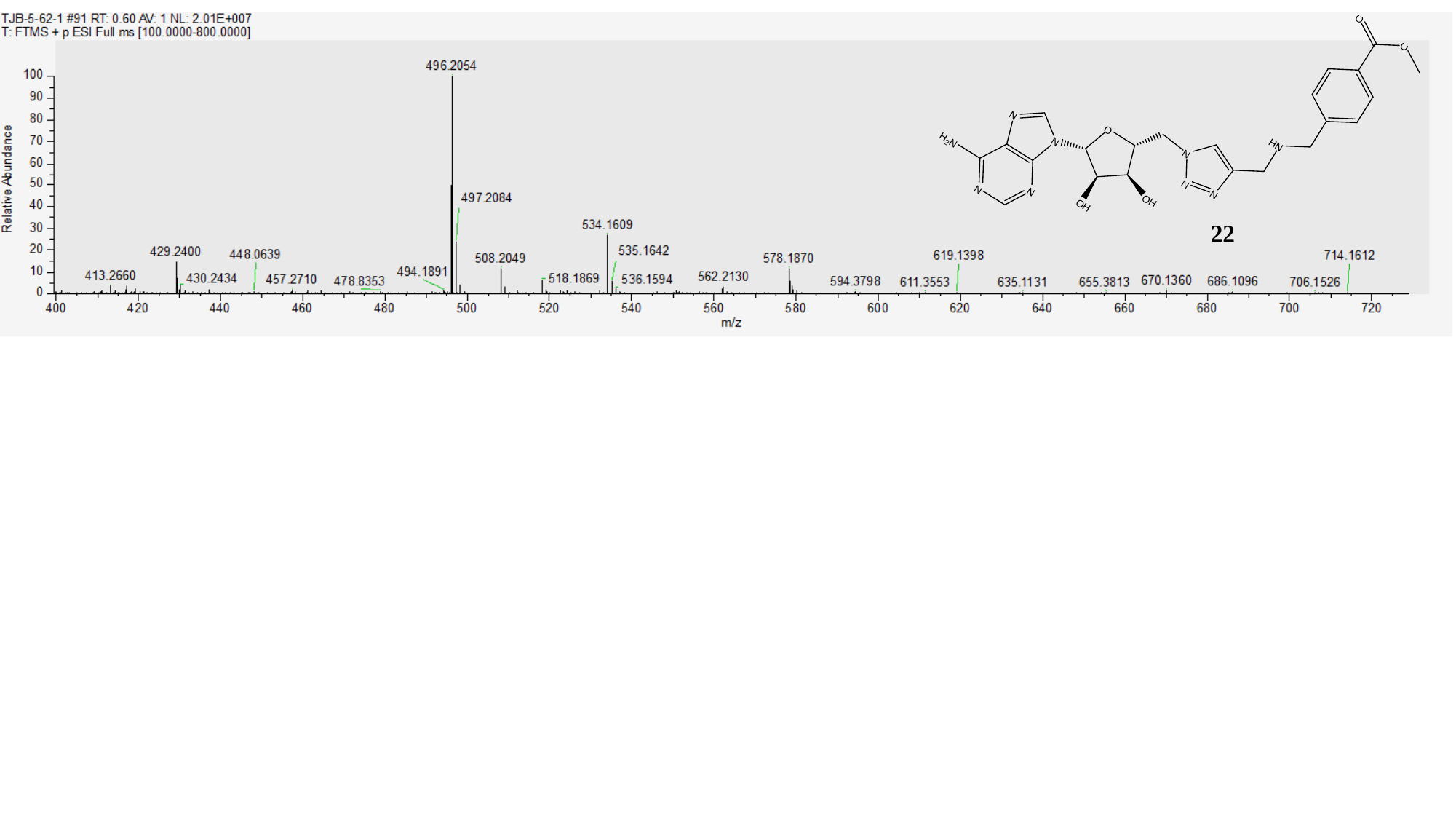

22

## Slide 66
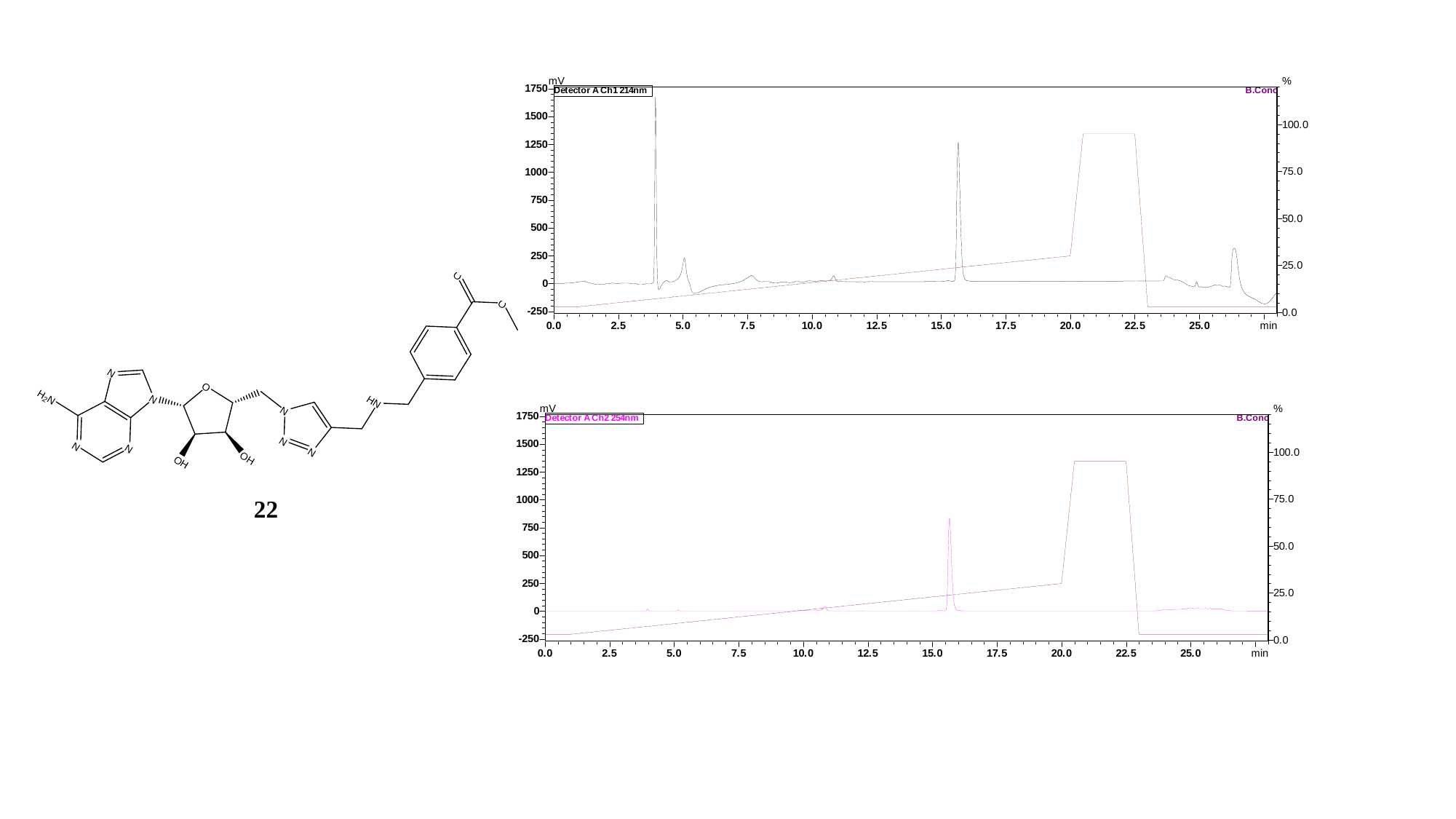

22

## Slide 67
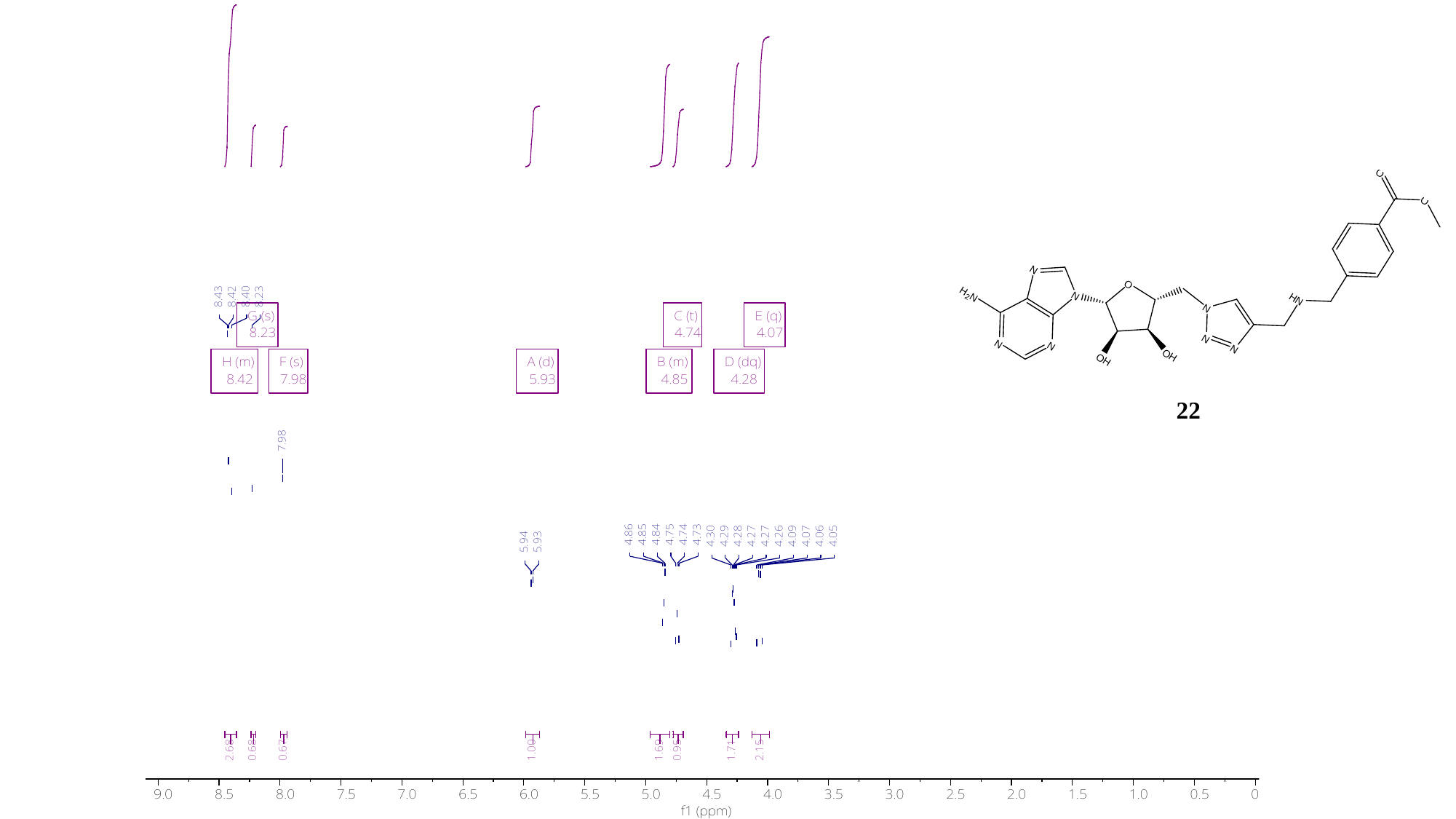

22

## Slide 68
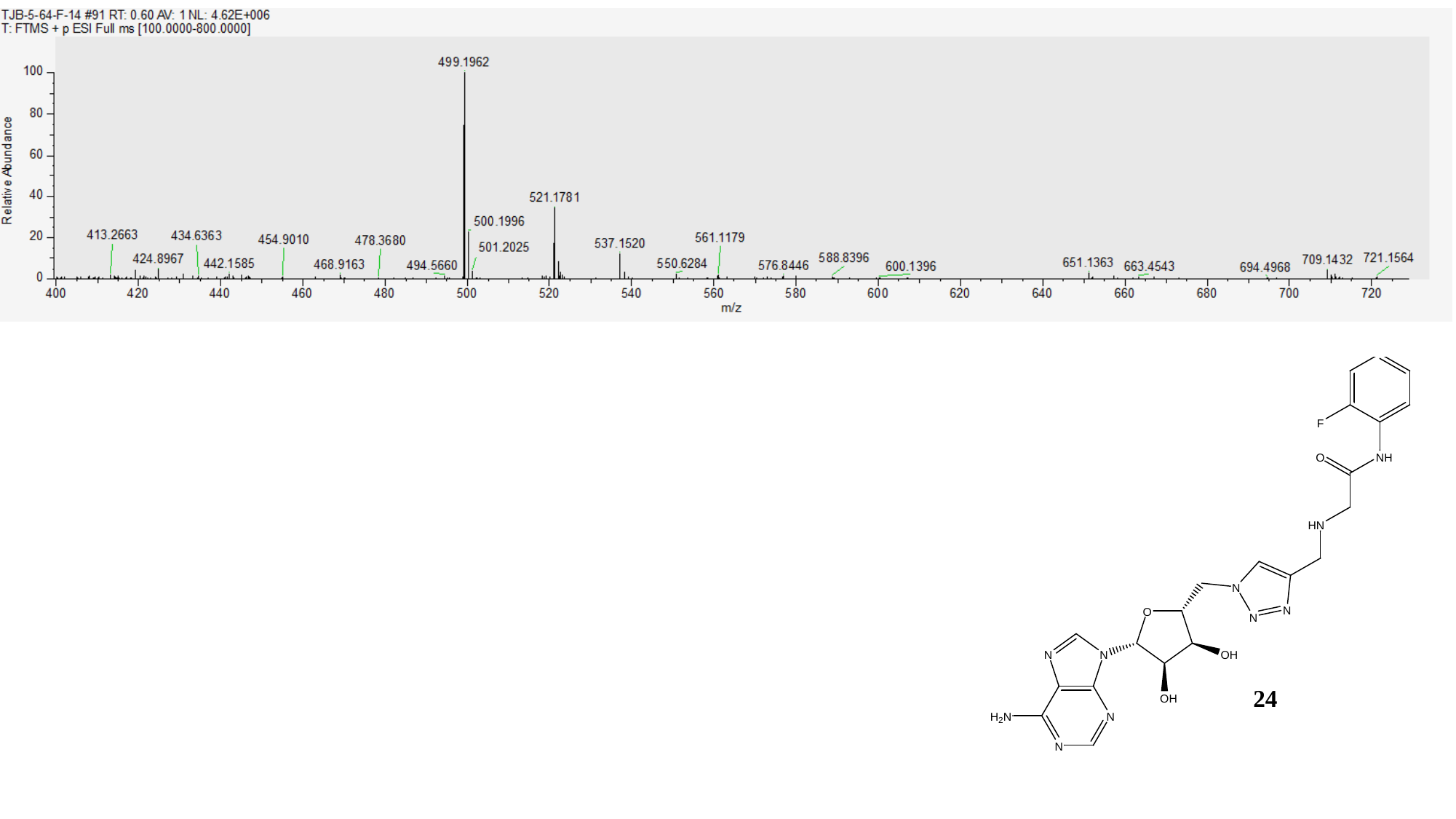

24

## Slide 69
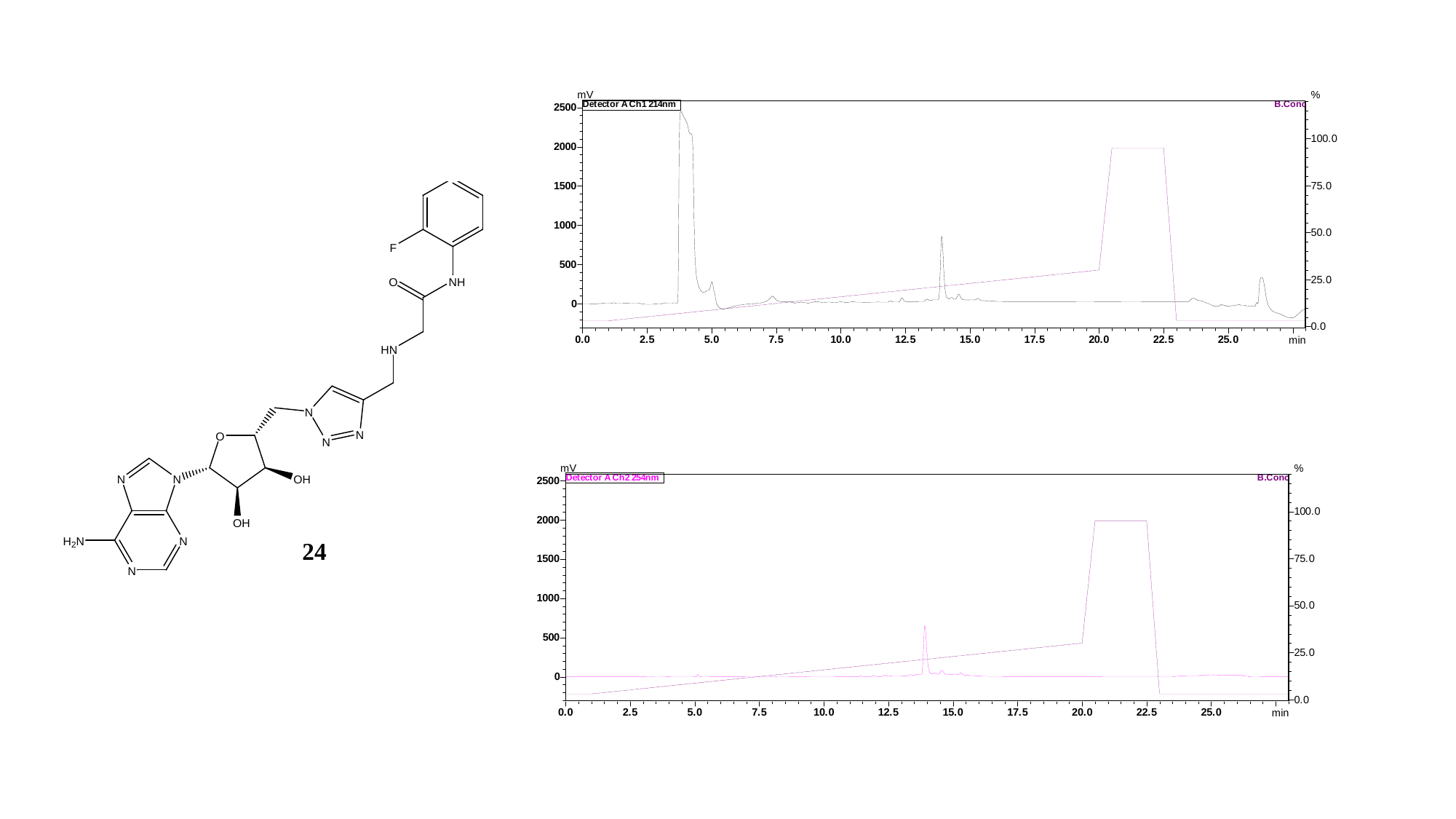

24

## Slide 70
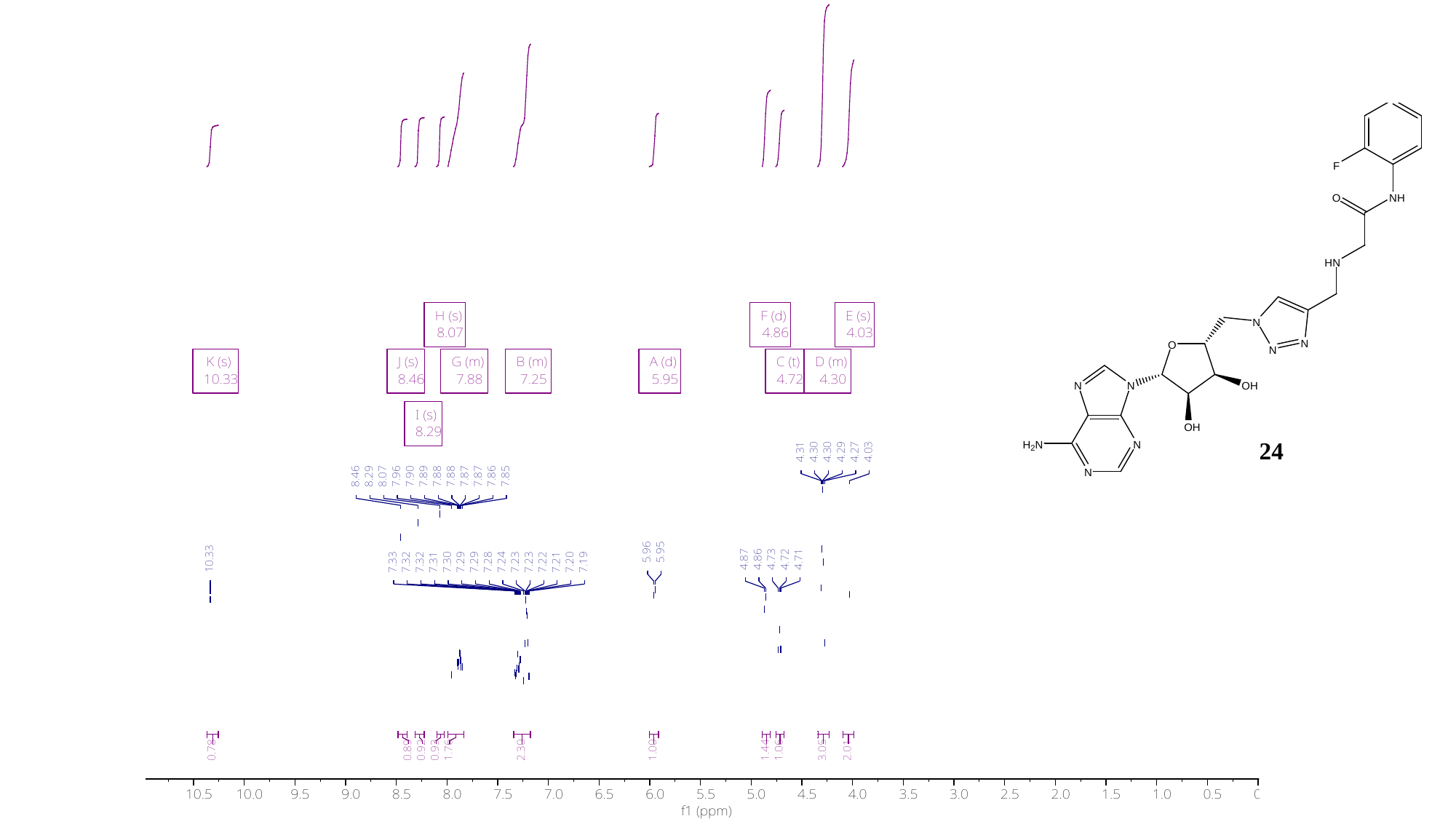

24

## Slide 71
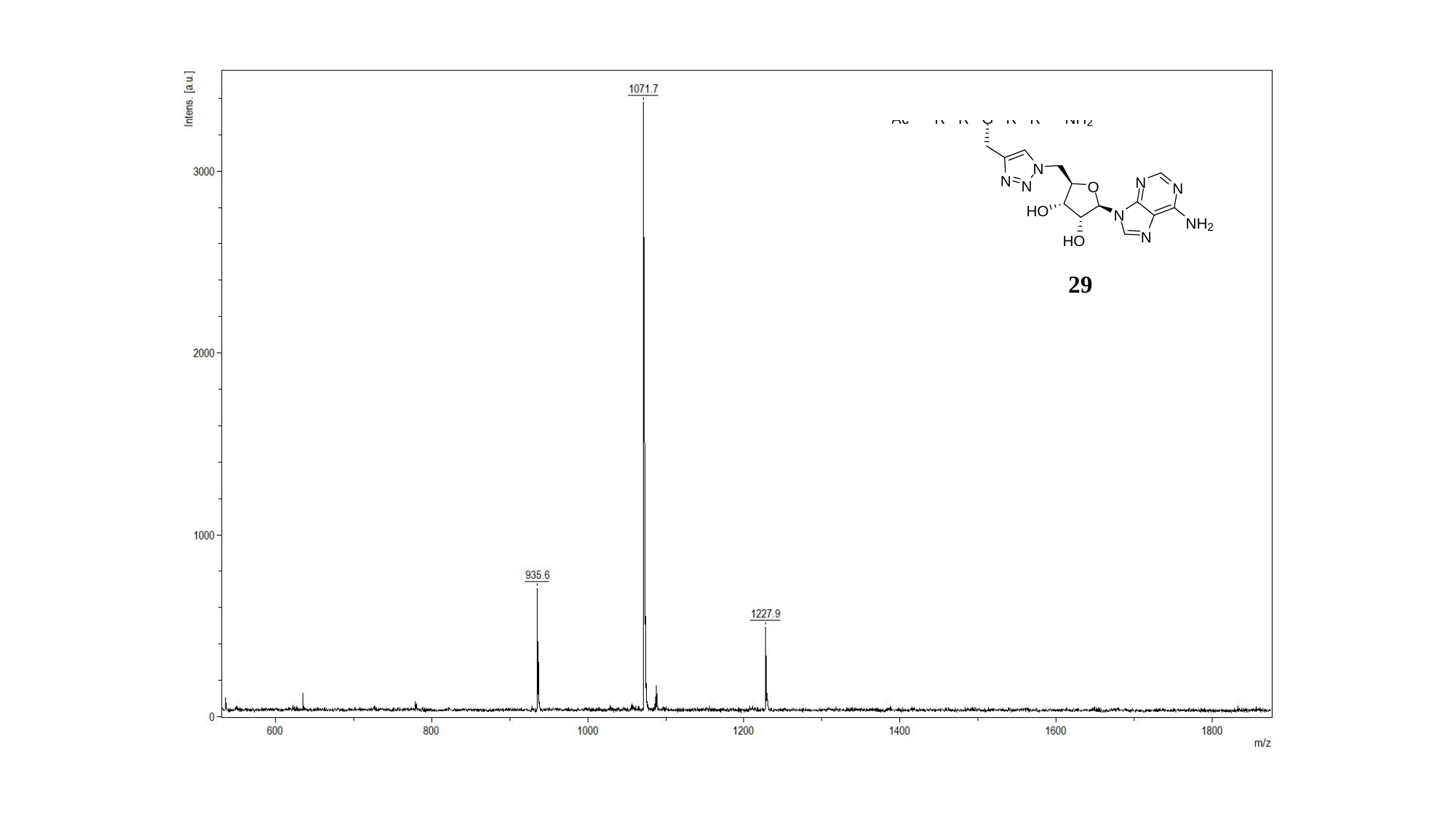

29

## Slide 72
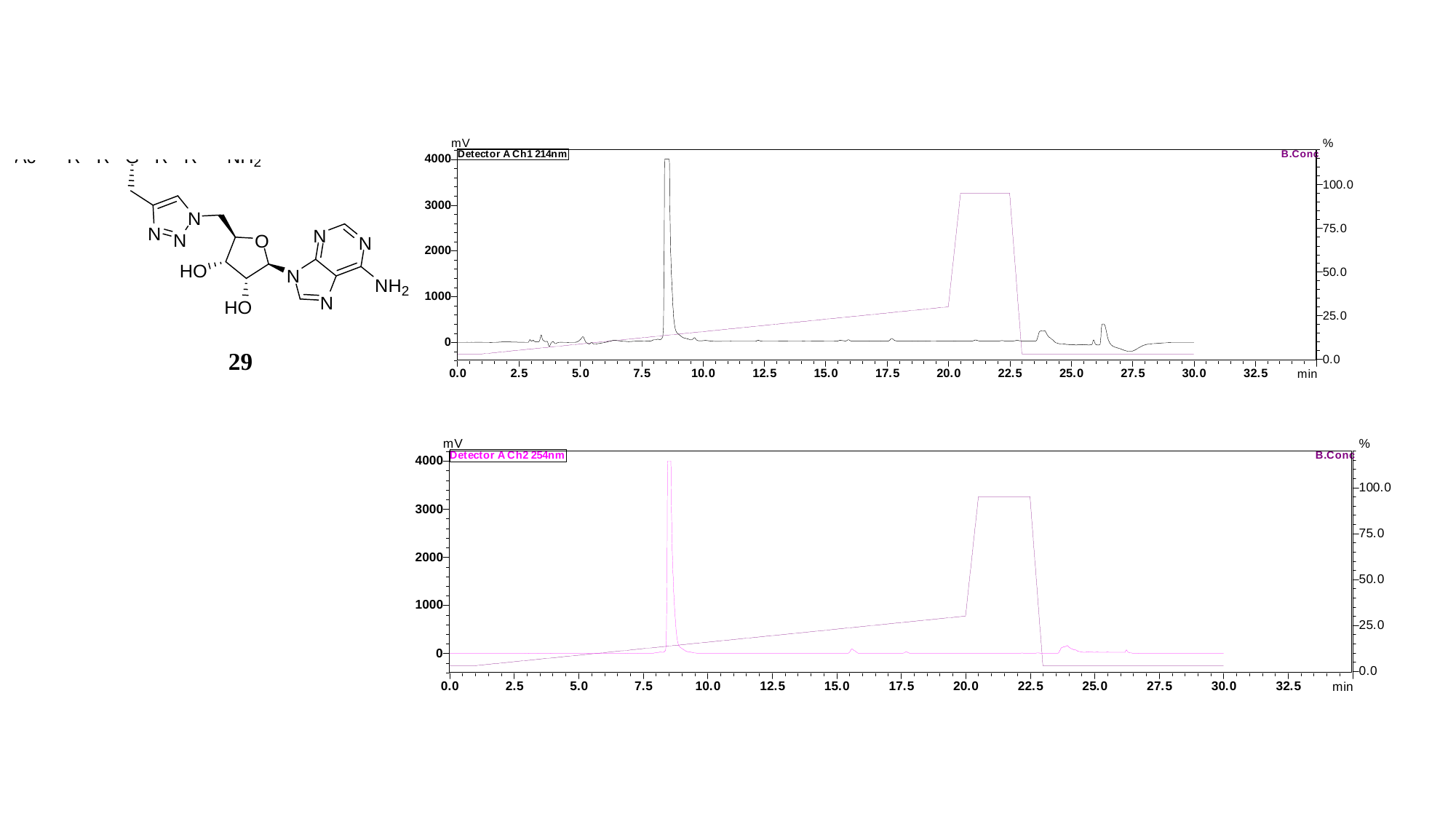

29

## Slide 73
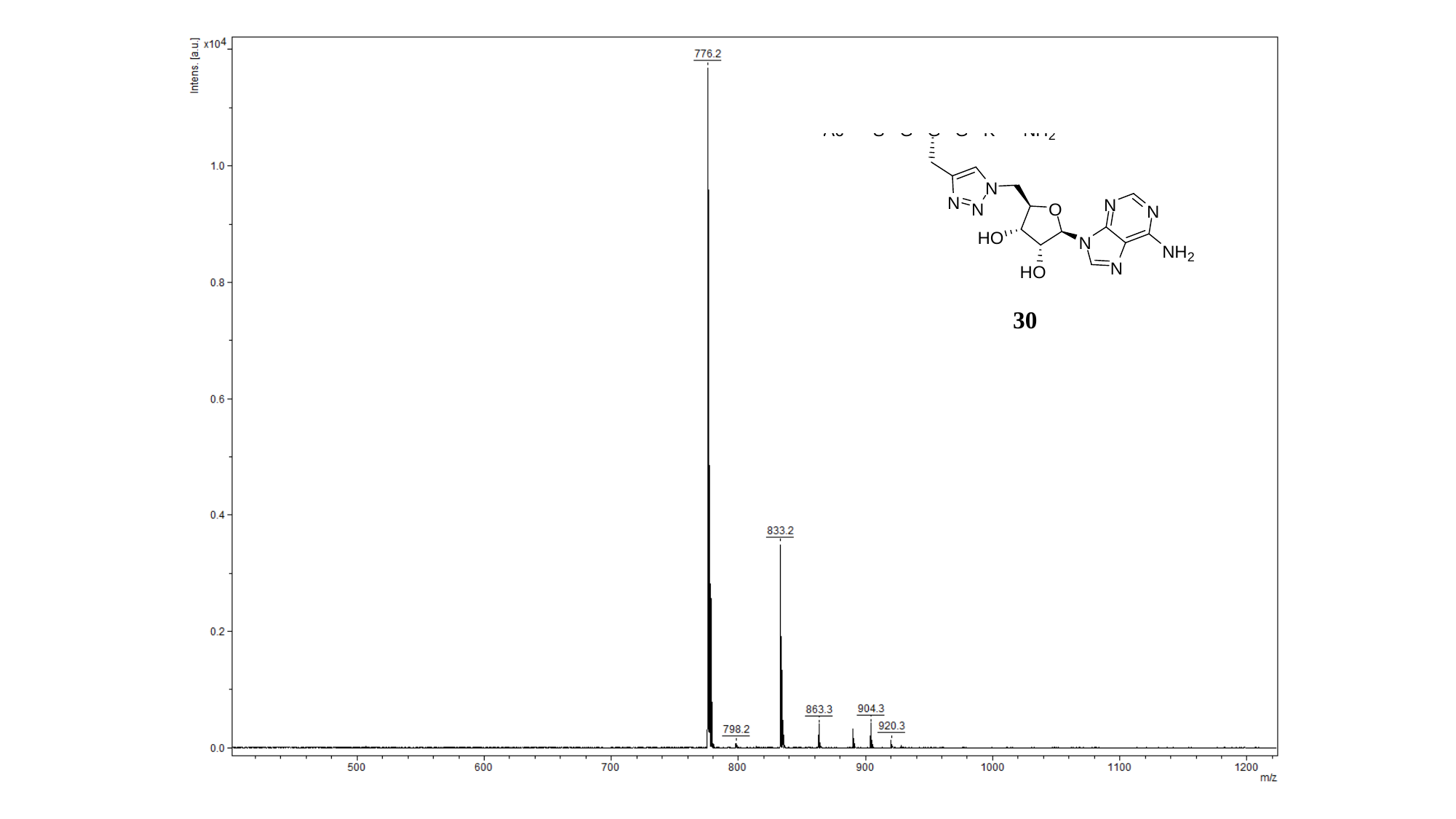

30

## Slide 74
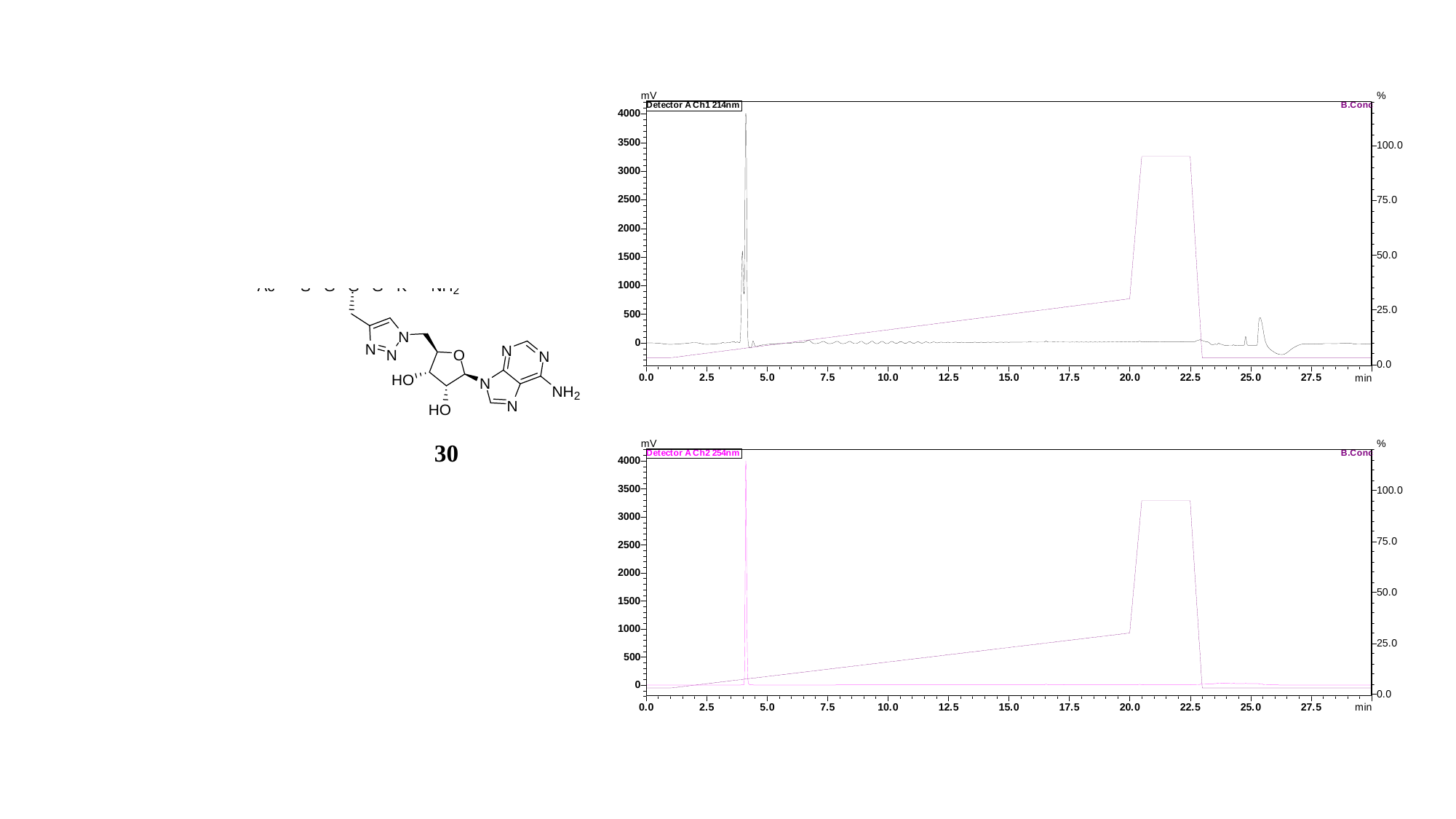

30
